# The Potential of ChatGPT as a Self-Diagnostic Tool in Common Orthopedic Diseases: Exploratory Study

**DOI:** 10.2196/47621

**Published:** 2023-09-15

**Authors:** Tomoyuki Kuroiwa, Aida Sarcon, Takuya Ibara, Eriku Yamada, Akiko Yamamoto, Kazuya Tsukamoto, Koji Fujita

**Affiliations:** 1 Department of Orthopaedic and Spinal Surgery Graduate School of Medical and Dental Sciences Tokyo Medical and Dental University Tokyo Japan; 2 Division of Orthopedic Surgery Research Mayo Clinic Rochester, MN United States; 3 Department of Surgery Mayo Clinic Rochester, MN United States; 4 Department of Functional Joint Anatomy Graduate School of Medical and Dental Sciences Tokyo Medical and Dental University Tokyo Japan; 5 Division of Medical Design Innovations Open Innovation Center, Institute of Research Innovation Tokyo Medical and Dental University Tokyo Japan

**Keywords:** ChatGPT, generative pretrained transformer, natural language processing, artificial intelligence, chatbot, diagnosis, self-diagnosis, accuracy, precision, language model, orthopedic disease, AI model, health information

## Abstract

**Background:**

Artificial intelligence (AI) has gained tremendous popularity recently, especially the use of natural language processing (NLP). ChatGPT is a state-of-the-art chatbot capable of creating natural conversations using NLP. The use of AI in medicine can have a tremendous impact on health care delivery. Although some studies have evaluated ChatGPT’s accuracy in self-diagnosis, there is no research regarding its precision and the degree to which it recommends medical consultations.

**Objective:**

The aim of this study was to evaluate ChatGPT’s ability to accurately and precisely self-diagnose common orthopedic diseases, as well as the degree of recommendation it provides for medical consultations.

**Methods:**

Over a 5-day course, each of the study authors submitted the same questions to ChatGPT. The conditions evaluated were carpal tunnel syndrome (CTS), cervical myelopathy (CM), lumbar spinal stenosis (LSS), knee osteoarthritis (KOA), and hip osteoarthritis (HOA). Answers were categorized as either correct, partially correct, incorrect, or a differential diagnosis. The percentage of correct answers and reproducibility were calculated. The reproducibility between days and raters were calculated using the Fleiss κ coefficient. Answers that recommended that the patient seek medical attention were recategorized according to the strength of the recommendation as defined by the study.

**Results:**

The ratios of correct answers were 25/25, 1/25, 24/25, 16/25, and 17/25 for CTS, CM, LSS, KOA, and HOA, respectively. The ratios of incorrect answers were 23/25 for CM and 0/25 for all other conditions. The reproducibility between days was 1.0, 0.15, 0.7, 0.6, and 0.6 for CTS, CM, LSS, KOA, and HOA, respectively. The reproducibility between raters was 1.0, 0.1, 0.64, –0.12, and 0.04 for CTS, CM, LSS, KOA, and HOA, respectively. Among the answers recommending medical attention, the phrases “essential,” “recommended,” “best,” and “important” were used. Specifically, “essential” occurred in 4 out of 125, “recommended” in 12 out of 125, “best” in 6 out of 125, and “important” in 94 out of 125 answers. Additionally, 7 out of the 125 answers did not include a recommendation to seek medical attention.

**Conclusions:**

The accuracy and reproducibility of ChatGPT to self-diagnose five common orthopedic conditions were inconsistent. The accuracy could potentially be improved by adding symptoms that could easily identify a specific location. Only a few answers were accompanied by a strong recommendation to seek medical attention according to our study standards. Although ChatGPT could serve as a potential first step in accessing care, we found variability in accurate self-diagnosis. Given the risk of harm with self-diagnosis without medical follow-up, it would be prudent for an NLP to include clear language alerting patients to seek expert medical opinions. We hope to shed further light on the use of AI in a future clinical study.

## Introduction

Recently, the field of artificial intelligence (AI) has made remarkable progress. The applications of AI in health care have also gained attention [1-5]. One of the most popular forms of AI involves using a natural language processing (NLP) system. In medicine, researchers have used NLP to extract unstructured data from medical records, followed by organization of the output [6-9]. Some have advocated for the use of an NLP as a prognostic or diagnostic tool [10-12]; however, further investigation is warranted. ChatGPT (OpenAI, San Francisco, CA, USA) was released in November 2022. ChatGPT is a sophisticated chatbot that uses an NLP model capable of both supervised and forced learning; it can understand the context of a sentence from only a few words. ChatGPT is also thought to possess the ability to translate languages and analyze customer experience if implemented as a survey [13]. Hence, its popularity has been growing rapidly [14]. Despite not being explicitly designed for health care, ChatGPT has also been increasingly used in health care contexts [3,15]. ChatGPT can be helpful in aiding health care providers in formulating differential diagnoses or assisting patients in self-diagnosing conditions before seeking medical attention. Nonetheless, it is still unclear whether digital self-diagnostic tools truly provide health benefits to patients, and multiple studies have raised concerns about their accuracy in triage and diagnosis [16-20]. If we leap into the realm of AI and its health care applications, we must first understand whether ChatGPT can accurately and precisely assist with self-diagnosis to reduce the risk of error, which would cause harm to the patient. The clinical significance of this application of ChatGPT is that patients would have access to a readily available platform to diagnose a condition correctly and later seek medical attention for management. However, few studies have evaluated the accuracy of ChatGPT’s ability to support self-diagnosis [21,22].

In addition to accuracy, it is equally important to evaluate precision, since it is challenging to rely on a self-diagnostic tool that provides inconsistent answers across different days and users. Additionally, an AI chatbot is not a substitute for medical care and should appropriately recommend seeking medical consultation after self-diagnosis. However, there is no research evaluating both the precision of ChatGPT’s responses and the degree to which it recommends medical attention.

Therefore, the purpose of this study was to assess the accuracy and precision of ChatGPT in self-diagnosis and to assess the degree of medical provider recommendation in its answers. We evaluated five common orthopedic symptoms/diseases since orthopedic complaints are very common in practice as they comprise up to 26% of the reasons why patients seek care [23]. For each of the diseases, we submitted a few characteristic symptoms to ChatGPT, and then we evaluated the accuracy (percentage of correct responses) and precision of the chatbot’s responses.

## Methods

### Ethical Considerations

Ethical review was not required since our research uses neither humans, animals, nor any of their information.

### Study Design

Over a 5-day period (February 20 to 24, 2023, between the hours of 12 AM and 3 PM), the study authors (TI, EY, AY, KT, and KF) submitted the same questions to ChatGPT (GPT version 3.5) (see Multimedia Appendix 1 for an example). Each question was submitted daily to evaluate the variation in responses. At the end of the study period, all answers generated by the chatbot were recorded and sent to one study author (TK) for analysis. Additionally, each author who questioned ChatGPT provided the details of the operating system (OS) and browser software they used when conducting this experiment.

### Diseases and Questions

We evaluated five common orthopedic diseases: carpal tunnel syndrome (CTS), cervical myelopathy (CM), lumbar spinal stenosis (LSS), knee osteoarthritis (KOA), and hip osteoarthritis (HOA). These diseases were chosen as they were felt to contain a wide variety of symptoms from joint and lower back pain to neuropathy, which are typical reasons for seeking care [23]. To help standardize a uniform set of questions, five orthopedic surgeons and one physical therapist engaged in discussions with English-speaking surgeons to obtain an expert consensus on common symptoms and plain-language questions. We also refined each question by using the Mayo Clinic [24,25], Cleveland Clinic [26,27], and Johns Hopkins Medicine [28,29] websites. The initial questions are listed in Textbox 1.

To identify means of improving the accuracy of ChatGPT’s self-assessment, nine additional questions (Textbox 2) were included in the study over a 5-day period (April 30 to May 4, 2023). Questions 1a, 2a, 3a, 4a, and 5a were in addition to the original questions, which required ChatGPT to provide a primary diagnosis along with five potential differential diagnoses. Question 2b was designed for cases where subjective symptoms of the patient with CM were limited to the upper extremities. Questions 1c, 4c, and 5c were rephrased due to concerns that questions 4 and 5, unlike question 1c, began with “My knee” or “My hip,” which might have reduced the accuracy and precision of the answers.

**Initial questions to assess five common orthopedic diseases.**

1．I have tingling and pain in my fingers (especially at night). I also have difficulty picking up small objects. What is this disease?

2．I have numbness in my hands. I also have difficulty doing fine movements to handle small objects, such as buttoning a shirt. I have an unsteady walk (especially when going downstairs). What is this disease?

3．I have pain in my lower back. I also have numbness and pain in my buttocks and calves. The pain increases when I have been walking for a while but improves when I lean slightly forward. What is this disease?

4．My knee is swollen and hurts when I walk. When bending my knee, I feel stiff and hear cracking. What is this disease?

5．My hip hurts when I walk. When moving my hip, I feel stiff and hear cracking. What is this disease?

**Refinement of questions to improve the accuracy of assessment.**

Q1a. I have tingling and pain in my fingers (especially at night). I also have difficulty picking up small objects. What is this disease? Can you give me a primary diagnosis and a list of five potential differential diagnoses?

Q1c. My fingers tingle and hurt (especially at night). I also have difficulty picking up small objects. What is this disease?

Q2a. I have numbness in my hands. I also have difficulty doing fine movements to handle small objects, such as buttoning a shirt. I have an unsteady walk (especially when going downstairs). What is this disease? Can you give me a primary diagnosis and a list of five potential differential diagnoses?

Q2b. I have numbness in my hands. I also have difficulty doing fine movements to handle small objects, such as buttoning a shirt. What is this disease?

Q3a. I have pain in my lower back. I also have numbness and pain in my buttocks and calves. The pain increases when I have been walking for a while but improves when I lean slightly forward. What is this disease? Can you give me a primary diagnosis and a list of five potential differential diagnoses?

Q4a. My knee is swollen and hurts when I walk. When bending my knee, I feel stiff and hear cracking. What is this disease? Can you give me a primary diagnosis and a list of five potential differential diagnoses?

Q4c. I have knee swelling and pain when I walk. When bending my knee, I feel stiff and hear cracking. What is this disease?

Q5a. My hip hurts when I walk. When moving my hip, I feel stiff and hear cracking. What is this disease? Can you give me a primary diagnosis and a list of five potential differential diagnoses?

Q5c. I have hip swelling and pain when I walk. When moving my hip, I feel stiff and hear cracking. What is this disease?

### Accuracy Assessment

**Figure 1:**
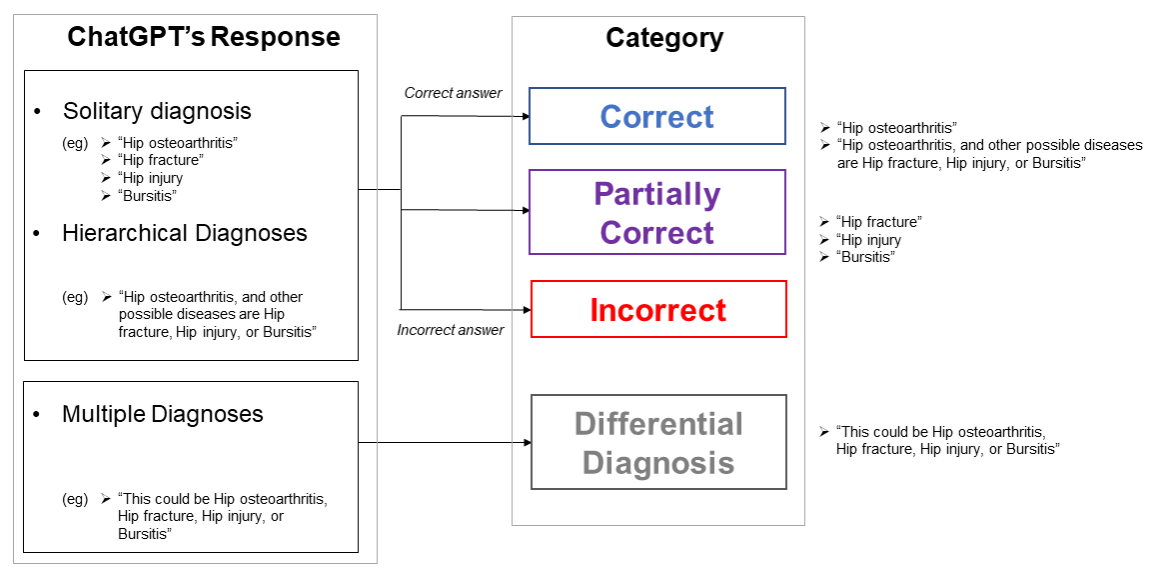
Accuracy assessment as defined by the study. After submitting the study questions to ChatGPT, the responses generated were either categorized as “solitary diagnosis,” “hierarchical diagnosis,” or “multiple diagnoses.” The correctness of the response was evaluated, except for the “multiple diagnoses” as it was considered its own category.

One of the study authors (TK), who did not pose questions to ChatGPT, evaluated the responses of ChatGPT (see Multimedia Appendix 1). The responses were categorized as shown in Figure 1. Briefly, they were either (1) one solitary diagnosis, (2) hierarchical diagnoses with other potential causes, and (3) multiple diagnoses. “Solitary diagnosis” encompassed cases where only one possible diagnosis was raised in the response. “Hierarchical diagnoses” involved cases where a single most likely diagnosis was provided in the response, followed by several other possible diagnoses. “Multiple diagnoses” involved cases where multiple possible diagnoses were presented without hierarchy in the response. If an answer included one solitary answer or hierarchical diagnoses, it was then evaluated for correctness. If a solitary diagnosis or the top diagnosis in the hierarchical diagnoses was correct, the answer was considered correct; if the correct diagnosis was included among the other possible diagnoses in the hierarchical diagnoses, it was considered partially correct. In the case of multiple diagnoses, the response was categorized as a differential diagnosis. Lastly, if neither of the prior phrases occurred in the response, it was categorized as incorrect.

### Precision Assessment

**Figure 2:**
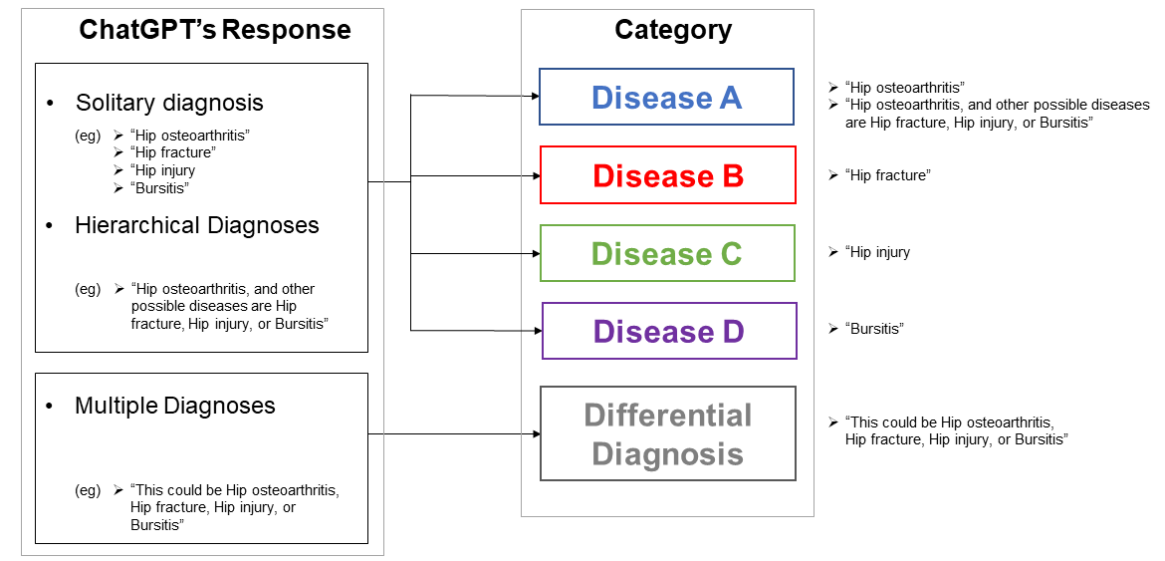
Precision assessment as defined by the study. Similar to the format used for accuracy assessment shown in Figure 1, ChatGPT either reported a “solitary diagnosis,” “hierarchical diagnosis,” or “multiple diagnoses.” For either a “solitary diagnosis” or “hierarchical diagnosis," the most probable diagnosis was categorized into the reported disease (ie, Disease A, B, C, D). Multiple diagnoses comprised a separate category. The responses were evaluated daily.

The precision assessment is shown in Figure 2. To assess the variability of responses, we evaluated the precision of the chatbot’s ability to diagnose each disease. The same three responses were seen as described above. We evaluated the number of times a solitary disease or a differential diagnosis was answered daily.

Additionally, the incorrect answer ratio within answers that presented solitary or hierarchical diagnoses was calculated separately. Note that differential diagnoses were excluded from the denominator.

### Recommendations

To evaluate the extent to which ChatGPT recommended seeking care, we searched for words that included the terms “medical,” “health care,” “doctor,” or similar terminology. Subcategories were analyzed by the percentage of each phrase reported. We evaluated the strength of each phrase. We defined a strong recommendation when the phrases included the word “essential” and/or “recommendation”; other phrases were considered to indicate a weaker recommendation.

Furthermore, the percentage of the number of words in an answer that was used to recommend seeking care was calculated using the following equation: number of words used to recommend seeking care/total number of words.

### Statistical Analysis

The precision and accuracy were calculated separately for each disease.

Accuracy was assessed using the “correct answer ratio,” which represents the average percentage of correct answers over the 5-day period. This value was obtained by using the average of the values reported by each rater. Similarly, the “error answer ratio” was defined as the average percentage of incorrect answers observed during the 5-day period.

For precision evaluation, the reproducibility between days and raters was evaluated separately. The number of raters was determined to be five, which was equal to the number of questions according to a previous study, which stated that the number of raters in a study assessing reliability between raters should be the same as the number of subjects [30]. For accuracy, the Fleiss κ coefficient between the categorical variables of the five answers in one rater was calculated and the median of the five values in five raters was regarded as the reproducibility between days [31]. For precision, the Fleiss κ coefficient between the answers on the same day was calculated and the mean over the 5 days served as the reproducibility between raters [31]. Fleiss κ coefficients were evaluated as follows: < 0, poor; 0.01-0.20, slight; 0.21-0.40, fair; 0.41-0.60, moderate; 0.61-0.80, substantial; and 0.81-1.00, almost perfect [32].

## Results

### Summary of Answers to the Questions

The summary of answers to the initial questions are presented in Table 1 and the full text of the answers is shown in Multimedia Appendix 2. In response to the question regarding CTS, ChatGPT diagnosed CTS in all answers. In response to the question regarding CM, ChatGPT either diagnosed peripheral neuropathy, multiple sclerosis, a neurological disorder, or presented differential diagnoses. Regarding the question about LSS, ChatGPT diagnosed either LSS or sciatica, or presented differential diagnoses. Regarding the questions about KOA and HOA, ChatGPT diagnosed KOA and HOA, respectively, or presented differential diagnoses.

The OS and browser software used by each rater when using ChatGPT are presented in Table 2.

**Table 1:** Diagnoses provided by ChatGPT in response to questions categorized by rater and day.

Question		Day 1	Day 2	Day 3	Day 4	Day 5
**I have tingling and pain in my fingers (especially at night). I also have difficulty picking up small objects. What is this disease?**						
	Rater 1	CTS^a^	CTS	CTS	CTS	CTS
	Rater 2	CTS	CTS	CTS	CTS	CTS
	Rater 3	CTS	CTS	CTS	CTS	CTS
	Rater 4	CTS	CTS	CTS	CTS	CTS
	Rater 5	CTS	CTS	CTS	CTS	CTS
**I have numbness in my hands. I also have difficulty doing fine movements to handle small objects, such as buttoning a shirt. I have an unsteady walk (especially when going downstairs). What is this disease?**						
	Rater 1	PN^b^	DD^c^	MS^d^	MS	MS
	Rater 2	DD	MS	MS	DD	PN
	Rater 3	MS	PN	PN	DD	MS
	Rater 4	PN	PN	PN	DD	ND^e^
	Rater 5	PN	PN	PN	PN	PN
**I have pain in my lower back. I also have numbness and pain in my buttocks and calves. The pain increases when I have been walking for a while but improves when I lean slightly forward. What is this disease?**						
	Rater 1	LSS^f^	LSS	LSS	LSS	LSS
	Rater 2	LSS	SC^g^	LSS	LSS	SC
	Rater 3	LSS	LSS	LSS	LSS	LSS
	Rater 4	DD	LSS	LSS	LSS	LSS
	Rater 5	LSS	LSS	LSS	LSS	LSS
**My knee is swollen and hurts when I walk. When bending my knee, I feel stiff and hear cracking. What is this disease?**						
	Rater 1	KOA^h^	KOA	KOA	KOA	KOA
	Rater 2	KOA	DD	DD	KOA	DD
	Rater 3	KOA	KOA	KOA	DD	KOA
	Rater 4	DD	DD	DD	DD	DD
	Rater 5	KOA	KOA	KOA	KOA	KOA
**My hip hurts when I walk. When moving my hip, I feel stiff and hear cracking. What is this disease?**						
	Rater 1	HOA^i^	HOA	HOA	HOA	HOA
	Rater 2	DD	HOA	HOA	DD	HOA
	Rater 3	HOA	HOA	HOA	DD	HOA
	Rater 4	DD	DD	DD	DD	DD
	Rater 5	HOA	HOA	HOA	HOA	HOA

^a^CTS: carpal tunnel syndrome.

^b^PN: peripheral neuropathy.

^c^DD: differential diagnosis; categorized when ChatGPT provided a differential diagnosis with no hierarchy.

^d^MS: multiple sclerosis.

^e^ND: neurological disorder; judged as a correct answer because, although it is not the disease that was assumed, it is not an error.

^f^LSS: lumber spinal stenosis.

^g^SC: sciatica; judged as a correct answer because, although it is not the disease that was assumed, it is not an error.

^h^KOA: knee osteoarthritis.

^i^HOA: hip osteoarthritis.

**Table 2:** Operating system and browser software used by each rater.

Rater	Operating system	Browser software
1	Windows 10	Google Chrome
2	Windows 11	Google Chrome
3	iOS 15.5	Safari
4	Mac Monterey 12.1	Google Chrome
5	Mac Monterey 12.1	Safari

### Accuracy Assessment

**Figure 3:**
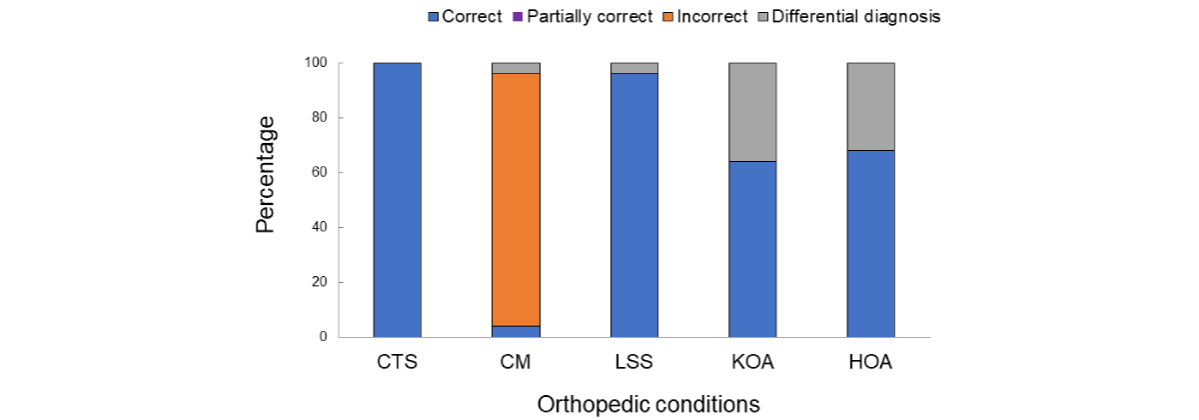
Correct answer ratio of each of the tested orthopedic conditions. CM had the highest incorrect answer choice and CTS had the highest percent correct. CM: cervical myelopathy; CTS: carpal tunnel syndrome; HOA: hip osteoarthritis; KOA: knee osteoarthritis; LSS: lumbar spinal stenosis.

The correct answer ratios varied for each disease (Figure 3). The ratios were 25/25 (100%) for CTS, 1/25 (4%) for CM, 24/25 (96%) for LSS, 16/25 (64%) for KOA, and 17/25 (68%) for HOA. Only CM had a high error answer ratio (23/25, 92%), whereas the error ratio was 0/25 (0%) for the other conditions.

The error answer ratio within answers that presented solitary diagnoses was 93% (16/17) for CM only and 0% for the others (0/18 for CTS, 0/20 for LSS, 0/7 for KOA, and 0/7 for HOA). The error answer ratio within answers that presented hierarchical diagnoses was 100% (7/7) for CM only and 0% for the others (0/7 for CTS, 0/4, 0/9 for KOA, and 0/9 for HOA).

**Figure 4:**
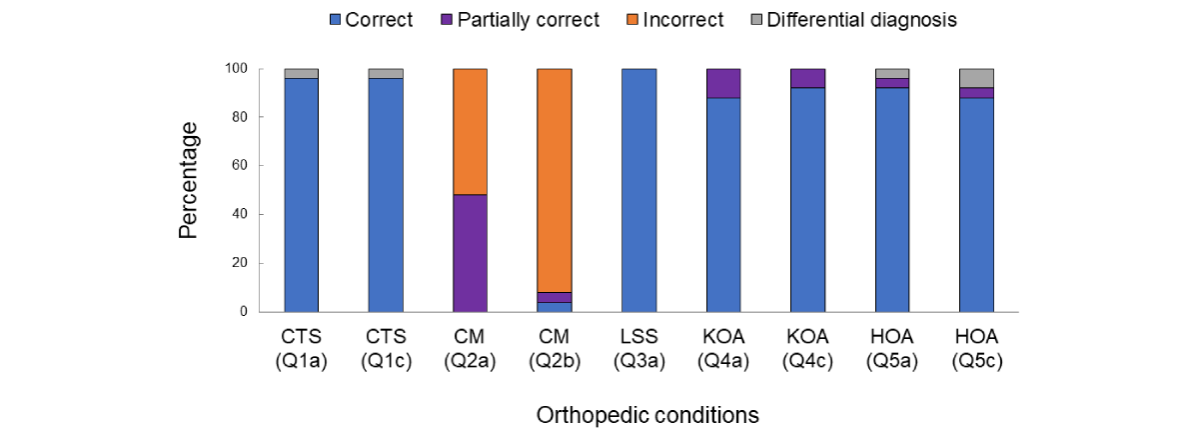
Correct answer ratio of the answers to the additional questions (see Textbox 2). CM: cervical myelopathy; CTS: carpal tunnel syndrome; HOA: hip osteoarthritis; KOA: knee osteoarthritis; LSS: lumbar spinal stenosis.

The full text of the answers to the additional questions is shown in Multimedia Appendix 3. The correct answer ratios for the additional questions (Textbox 2) varied for each disease (Figure 4): 24/25 (96%) for Q1a (CTS), 24/25 (96%) for Q1c (CTS), 0/25 (0%) for Q2a (CM), 1/25 (4%) for Q2b (CM), 25/25 (100%) for Q3a (LSS), 22/25 (88%) for Q4a (KOA), 23/25 (92%) for Q4c (KOA), 23/25 (92%) for Q5a (HOA), and 22/25 (88%) for Q5c (HOA). Only Q2a (CM) and Q2b (CM) received incorrect answers (13/25, 52% and 23/25, 92%, respectively) and other questions received no incorrect answers. In the answers to Q2b, CTS, which was not presented in the answer for the original CM question (Question 2), appeared with a rate of 80%.

Except for the answers to Q2a (CM) and Q2b (CM), all other answers showed high percentages of correct answer ratios. Approximately half of the answers to Q2a (CM) were partially correct.

### Precision Assessment

**Figure 5:**
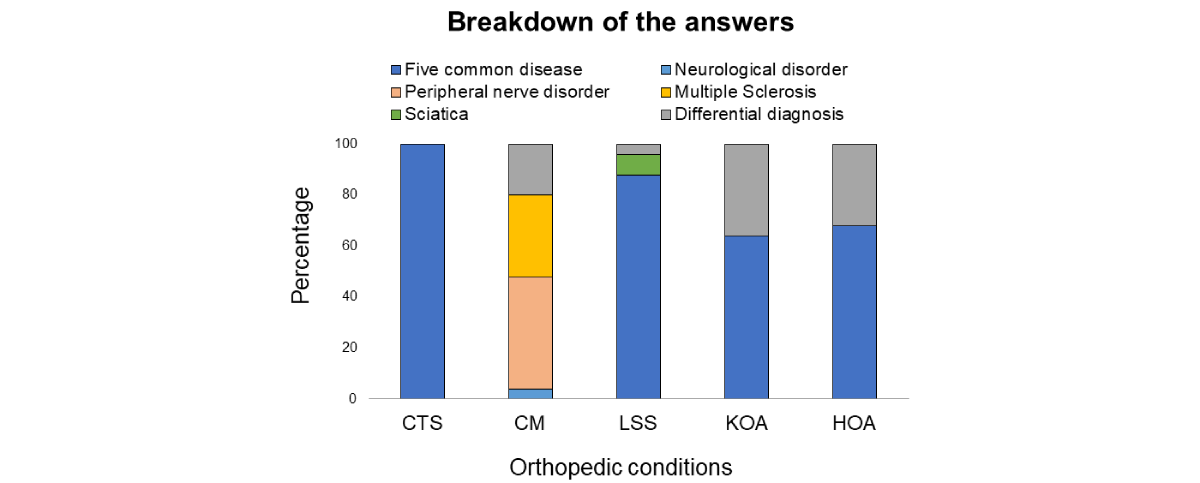
Precision assessment. The ratio of the presented responses by ChatGPT are shown. A reproducibility coefficient of 1.00 was defined as perfect precision. CM: cervical myelopathy; CTS: carpal tunnel syndrome; HOA: hip osteoarthritis; KOA: knee osteoarthritis; LSS: lumbar spinal stenosis.

Figure 5 shows the ratio of presented diseases and differential diagnoses among the answers. Reproducibility between days was 1.0, 0.15, 0.7, 0.6, and 0.6 for CTS, CM, LSS, KOA, and HOA, respectively. Reproducibility between the raters was 1.0, 0.1, 0.64, –0.12, and 0.04 for CTS, CM, LSS, KOA, and HOA, respectively. Daily and per-rater Fleiss κ and P values are listed in Multimedia Appendix 4.

### Recommendations

Table 3 shows the results on recommendations. The following key phrases were found: “essential,” “recommended,” “best,” and “important.” Many of the answers included only the word “important,” whereas only a few answers used strong words such as “essential” and “recommended.” Additionally, some answers did not provide any recommendations.

Overall, 16 out of 125 (12.8%) answers contained a word count percentage of 20% or more, indicating a recommendation for medical consultation, whereas 71 out of 125 (56.8%) answers had a percentage between 10% and 20%, 31 out of 125 (24.8%) of all answers had a percentage between 0% and 10%, and 7 out of 125 (5.6%) answers did not include any of these words.

**Table 3:** Phrases used to recommend seeking medical care (N=125).

Phrase	Frequency of use, n (%)
Essential	4 (3.2)
Recommend	12 (9.6)
Best	8 (6.4)
Important	94 (75.2)
None	7 (5.6)

## Discussion

### Principal Findings

This is the first study to evaluate ChatGPT’s ability to self-diagnose. Over a 5-day period, we submitted common symptoms to ChatGPT and evaluated its response for accuracy and precision. Generally, ChatGPT had the ability to generate high correct answer ratios, with the exception of the self-diagnosis of CM. Reproducibility was variable and disease-dependent. These results suggest that ChatGPT is inconsistent in both accuracy and precision to self-diagnose in its current form. By having ChatGPT present the five possible differential diagnoses, the ratio of correct answers for the questions on KOA and HOA was increased and the error answer ratio for the question on CM was decreased. Additionally, avoiding starting the question with “My knee” or “My hip” further improved the ratio of correct answers for KOA and HOA.

### Comparison With Previous Studies

Hirosawa et al [21] used ChatGPT to formulate a differential diagnosis. They found a 53.3% correct answer ratio. In our study, the correct answer ratio was similar in range (66.4%). However, there are several key differences between our studies. First, we evaluated orthopedic conditions, whereas Hirosawa et al [21] focused on systemic symptoms as pertinent to diseases seen by an internist. Since systemic diseases are not site-specific (ie, fever and rash seen with lupus), this could potentially explain their lower accuracy score. Their submissions also included objective findings such as physical exam and vital signs. We deliberately omitted such findings to simulate a natural setting in which a patient would use ChatGPT for self-diagnosis. This promotes the generalizability of the questions from a patient’s perspective. However, a study that evaluates the inclusion of objective findings and differences in accuracy/precision would be helpful in the future. Johnson et al [22] conducted an extensive inquiry with ChatGPT posing numerous medical questions and showed that the median accuracy of answers was fairly high. One might assume that their results demonstrated relatively higher accuracy compared to that obtained in our study and in that of Hirosawa et al [21] because the questions were more medically detailed. However, the mean accuracy was slightly lower than its median, and the authors discussed that this difference reflected multiple surprisingly incorrect answers provided by ChatGPT. Since we also found significant variation in accuracy among answers in our study, the discussion of Hirosawa et al [21] aligns with and supports our results.

### Accuracy Assessment

CTS (100%) and LSS (96%) had the highest correct answer ratios, which were much lower for KOA (64%) and HOA (68%). One potential cause for this difference is that both KOA and HOA did not include disease-specific symptoms despite typical symptoms provided to ChatGPT. This suggests that ChatGPT was unable to narrow down the answers. Interestingly, the error answer ratios were 0% in all four diseases. Of the diseases, CM had the lowest correct answer ratio at only 4%. Given the symptoms, ChatGPT generated several potential answers, which included a neurological disorder, peripheral nerve disorder, and multiple sclerosis. Unfortunately, CM was not identified. One potential reason for this could be attributed to the multifocal symptoms of CM (involving both the hands and feet), unlike the other conditions that may be more regional (ie, CTS). This suggests that ChatGPT is incapable of localizing a disease that is multifocal. Another potential reason is that the site of the disease and the site of symptom manifestation are not always the same in cases of CM. In this study, the question regarding CM did not include any symptoms specific to the neck. Alternatively, the question concerning LSS involved lumbar pain symptoms. This disparity may have caused the variation in the ratios of correct answers observed between these two conditions.

The low correct answer ratio in our study would suggest a risk of misdiagnosis and potential harm to the patient if this NLP tool is used in its current form. However, ChatGPT is a fine-tuned version of a chatbot, in which supervised and forced learning have been added to version GPT-3; thus, if ChatGPT had been educated on specific medical terms during this additional learning, a far higher degree of accuracy could have been achieved by incorporating those terms into our questions. Otherwise, this could have been overcome by including more site-specific symptoms when submitting the questions. We plan to conduct additional study to determine which question formats/words will increase the accuracy of self-diagnostic support provided by ChatGPT.

Although we asked simple and concise questions in this study, patients may ask more complex and difficult questions. It has been suggested that ChatGPT lacks “factual correctness” [33] and may provide inaccurate information, especially when tasked to provide a specific answer to an esoteric question [34]. To achieve a higher ratio of correct diagnoses for complex diseases in the context of self-diagnosis supported by ChatGPT, the questions may need to be stratified in a similar manner to that of an actual medical interview. Unfortunately, as seen in Multimedia Appendixes 2 and 3, at least the 3.5 version of ChatGPT did not attempt stratification (ie, ask clarifying questions back to the user) to increase the accuracy of the estimated diagnosis. However, the 4.0 version may return more in-depth questions. Otherwise, it is recommended that when developing an AI chat system specialized for medical self-diagnosis, it would be beneficial to incorporate a system that confidently asks follow-up questions to improve the accuracy of estimated diagnoses. Additionally, there is another notable concern that not only general users could be misinformed by ChatGPT, but even surgeons and physicians could pick up fraudulent papers generated by ChatGPT when seeking standardized medical responses [35-37]. This highlights the need for constant oversight of AI systems both in terms of design and usage. It is essential to involve government regulations and restrictions as well as conscientiousness from AI designers and the authors of the papers [38].

### Precision Assessment

Reproducibility varied and ranged from “poor” to “almost perfect,” even though we entered the same questions every time. The cause of this variability was unclear since the submissions were standardized at a fixed time and replicated among the raters. While the reproducibility between days exhibited moderate agreement for both KOA and HOA, the reproducibility between raters exhibited poor and slight agreement for KOA and HOA, respectively. The variability in responses may be a deliberate feature of ChatGPT since it mostly functions as a chatbot for social purposes. In this platform, it may be acceptable to have variable answers. However, if we are to apply this algorithm to health care, this variability may not be acceptable as it increases the risk of diagnostic error as made evident in the results. In the current form, ChatGPT has low reliability in self-diagnosing five common orthopedic conditions. It is also possible that ChatGPT may improve its reliability through learning, although this warrants further investigation. We could not detect any trends that would have caused differences in answers depending on the OS and browser software used. However, these factors might have decreased the reproducibility between raters.

### Recommendation for Medical Consultation

Nearly 5.6% of the generated answers omitted any recommendation to seek care. Since ChatGPT is not a substitute for medical advice, it would be prudent for the chatbot to counsel the patient to seek medical attention for diagnostic validation and management. Without this, the patient is left without guidance on the appropriate next steps. Some may think that this language is often written by a software or program to avoid medical liability should an error occur [17]. Since ChatGPT has inconsistent diagnostic capability, one would consider this a necessary feature should this be applied to health care. Although 79.6% of the answers recommended medical consultation for more than 10% of the total words, only 12.8% of the answers included a strong recommendation as set by the study standards with phrasing including either the term “essential” or “recommended.” The other phrases could be interpreted as rather vague since they indirectly recommend seeking care. Without direct language, it is possible that the patient is left confused after self-diagnosis, or worse, experience harm from a misdiagnosis. In fact, ChatGPT explicitly provides a disclaimer regarding these potential harms. Since it is not exclusively designed as a self-diagnostic tool for medical support, the inclusion of the disclaimer is understandably necessary. However, instead of solely focusing on limiting the use of AI chatbots for health care purposes to reduce the potential risk to users, several papers advocate that the following would be effective: (1) understanding and presenting the issues associated with the use of AI chatbots for health care purposes; (2) improving the technology and adapting it to appropriate health care applications; and (3) advocating for shared accountability and fostering collaboration among developers, subject matter experts, and human factors researchers [3,15,39]. Our study aligns with these recommendations as well.

### Additional Questions

The addition of the requirement to present the primary diagnosis and five potential differential diagnoses to the questions increased the ratios of correct answers for the questions on KOA and HOA (Q4a and Q5a in Textbox 2). This might have resulted from the higher frequency of knee and hip osteoarthritis, which was more likely to be selected as the primary diagnosis. Interestingly, CM was included within the potential differential diagnosis in approximately half of the answers to Q2a, reducing the error answer ratio to 52% because the percentage of partially correct answers increased. This would be a useful way to reduce the potential harm due to a misdiagnosis by ChatGPT.

Q2b, designed for CM with only upper-extremity symptoms, presented the same percentages of correct and incorrect answers as the original CM question (Question 2). However, 80% of those answers showed CTS, which was not diagnosed based on the original question. This may offer further evidence of the large influence of a site-specific factor on the diagnoses provided by ChatGPT.

The correct answer ratios increased for Q4c and Q5c, which were the questions modified to avoid phrases beginning with “My knee” or “My hip.” These results suggest that it may be better not to begin questions with phrases such as “My knee” when asking ChatGPT for a self-diagnosis.

As mentioned above, this study found that modifying the way the questions are presented and incorporating additional requirements can affect the accuracy of ChatGPT’s answers. A review of online symptom checkers found that incorporating regional or seasonal data along with personal data improved their accuracy ratio [18]. Incorporating such data in the questions posed to ChatGPT for self-diagnosis could lead to more accurate answers. Furthermore, a study recommended that self-diagnostic applications display the implicit diagnosis result with a percentage and present the rationale behind the diagnosis result [40]. At this time, adding these suggestions to the question posed to ChatGPT may yield more useful answers.

### Limitations

This study has several limitations. First, despite attempts to create questions that may simulate a patient’s question, they were not patient-derived questions. However, since this was a proof-of-concept study, it was felt that the questions would be sufficient to at least evaluate the accuracy and precision of the algorithm. We hope to address this limitation in future study since we will have patients submit their own questions. Second, we only tested five orthopedic diseases and thus this study may not represent the multitude of other orthopedic complaints. However, we felt that since these diseases are common, they warranted evaluation. Third, we did not compare our results using ChatGPT with those provided by other chatbots or publicly available data on medical conditions. Other chatbots may present better/worse results, and the easily accessible data do not always offer better support for self-diagnoses compared to that offered by chatbots. We plan to compare the difference between different chatbots in the future. Fourth, the OS and browser software used should have been consistent to eliminate their potential impact on the results. Fifth, it is possible that ChatGPT was trained using the six websites we referenced [24-29]. However, the significance of our study was not compromised and this was unrelated to the problem of reproducibility. Finally, a GPT-4 version of ChatGPT was released just after we conducted our experiment, which may provide more accurate answers. We plan to use this most recent version in our next study.

### Conclusion

This is the first study to evaluate ChatGPT’s ability to accurately and precisely self-diagnose five common orthopedic conditions. We found that ChatGPT was inconsistent with respect to self-diagnosis. Of the five diseases, CM had the lowest percent correct ratio, likely due to its multifocal symptoms, which suggests that ChatGPT is incapable of localizing symptoms for such widespread diseases. Given the risk of error and potential harm from misdiagnosis, it is important for any diagnostic tool to direct guidance to seek medical care for confirmation of a disease. A future study with more disease conditions and patient-derived questions can help shed light on the role of NLP as a diagnostic tool.

## Appendix

Multimedia Appendix 1 Screenshot of how to ask a question to ChatGPT (example). CTS: carpal tunnel syndrome.

Multimedia Appendix 2 Full text of the answers to the five questions for 5 days obtained by the study authors.

Multimedia Appendix 3 Full text of the answers to the nine additional questions for 5 days obtained by the study authors.

Multimedia Appendix 4 Daily and per-rater Fleiss κ and *P* values in the precision assessment.

## Abbreviations

AI: artificial intelligence
CM: cervical myelopathy
CTS: carpal tunnel syndrome
HOA: hip osteoarthritis
KOA: knee osteoarthritis
LSS: lumbar spinal stenosis
NLP: natural language processing
OS: operating system

## Data Availability

All data generated by ChatGPT during this study are included in the Multimedia Appendix files.
