# Supplementary figures and images for: The Potential of ChatGPT as a Self-Diagnostic Tool in Common Orthopedic Diseases: Exploratory Study
Source: J Med Internet Res. 2023 Sep 15;25:e47621. doi: 10.2196/47621 (PMC10541638; doi:10.2196/47621)

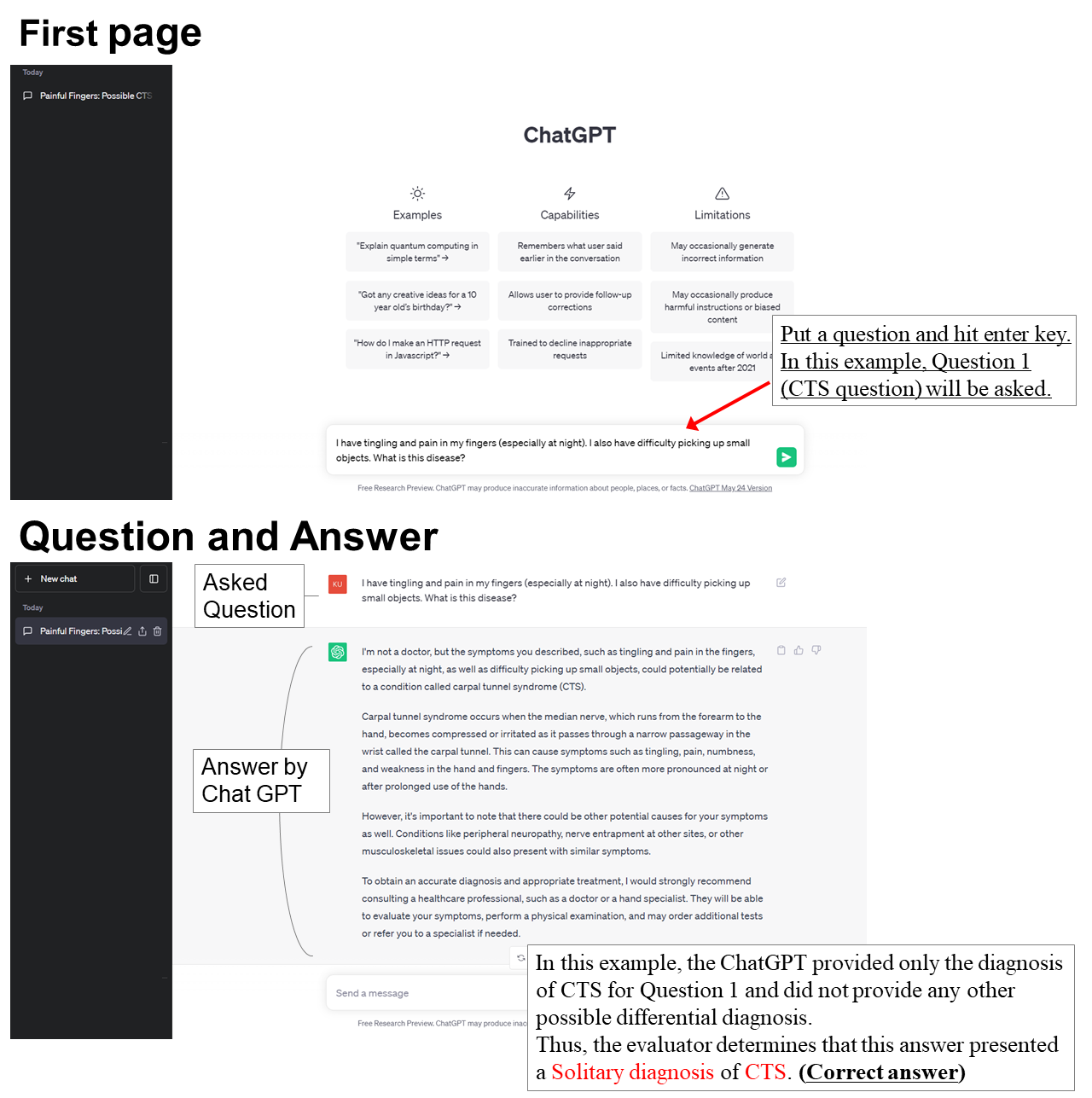

Supplement: Multimedia Appendix 1 [file jmir_v25i1e47621_app1.png]
