# Supplementary material for: The Potential of ChatGPT as a Self-Diagnostic Tool in Common Orthopedic Diseases: Exploratory Study
Source: J Med Internet Res. 2023 Sep 15;25:e47621. doi: 10.2196/47621 (PMC10541638; doi:10.2196/47621)
Supplement: Multimedia Appendix 2 [file jmir_v25i1e47621_app2.doc]

**Author, KF**

**Day 1**

A1.

The symptoms you described could be caused by several different conditions, but one possibility is carpal tunnel syndrome. This is a common condition where the median nerve, which runs through a narrow passage in the wrist called the carpal tunnel, becomes compressed or pinched.

The symptoms of carpal tunnel syndrome often include tingling or numbness in the fingers (especially the thumb, index, and middle fingers), pain or discomfort that may radiate up the arm, weakness in the hand, and difficulty gripping or holding onto objects. Symptoms are usually worse at night or first thing in the morning.

If you are experiencing these symptoms, it is recommended that you consult with a healthcare provider for a proper diagnosis and treatment plan. Treatment for carpal tunnel syndrome may include splinting, physical therapy, medications, or in some cases, surgery.

A2.

The symptoms you described could be caused by several different conditions, but one possibility is peripheral neuropathy. This is a condition that affects the peripheral nerves, which are the nerves that carry information from the brain and spinal cord to the rest of the body.

Peripheral neuropathy can cause numbness or tingling in the hands and feet, as well as weakness or difficulty with fine movements. It can also cause problems with balance and coordination, which could explain the unsteady walk, especially when going downstairs.

Peripheral neuropathy can be caused by a variety of factors, including diabetes, autoimmune diseases, vitamin deficiencies, infections, and certain medications or toxins. It is important to see a healthcare provider for a proper diagnosis and treatment plan.

Treatment for peripheral neuropathy may involve addressing the underlying cause, if possible, as well as medications to manage symptoms and physical therapy to improve strength and coordination.

A3.

The symptoms you described could be caused by a condition called spinal stenosis. Spinal stenosis is a narrowing of the spinal canal, which can put pressure on the spinal cord and nerves that run through it.

The pain in the lower back, as well as the numbness and pain in the buttocks and calves, are all common symptoms of spinal stenosis. The pain may worsen when walking or standing for a period of time because this can increase the pressure on the spinal nerves. Leaning slightly forward can help to alleviate the pain by opening up the spinal canal and relieving some of the pressure on the nerves.

Spinal stenosis can be caused by a variety of factors, including aging, herniated discs, osteoarthritis, or other spinal conditions. It is important to see a healthcare provider for a proper diagnosis and treatment plan.

Treatment for spinal stenosis may include physical therapy to improve strength and flexibility, medications to manage pain and inflammation, and in some cases, surgery to relieve pressure on the nerves.

A4.

The symptoms you described could be caused by a few different conditions, but one possibility is osteoarthritis of the knee. Osteoarthritis is a degenerative joint disease that occurs when the cartilage that cushions the joints breaks down over time, causing pain, swelling, stiffness, and limited mobility.

The swelling and pain you are experiencing, especially when walking, can be a common symptom of osteoarthritis in the knee. The stiffness and cracking sounds when bending the knee can also be characteristic of this condition.

Other possible causes of knee pain and stiffness include injuries, such as a sprain or strain, or inflammation, such as tendinitis or bursitis.

It is important to see a healthcare provider for a proper diagnosis and treatment plan. Treatment for osteoarthritis of the knee may include medications to manage pain and inflammation, physical therapy to improve strength and flexibility, and in some cases, surgery to repair or replace the affected joint.

A5.

The symptoms you described could be caused by a few different conditions, but one possibility is osteoarthritis of the hip. Osteoarthritis is a degenerative joint disease that occurs when the cartilage that cushions the joints breaks down over time, causing pain, swelling, stiffness, and limited mobility.

The pain you are experiencing, especially when walking, can be a common symptom of osteoarthritis in the hip. The stiffness and cracking sounds when moving the hip can also be characteristic of this condition.

Other possible causes of hip pain and stiffness include injuries, such as a strain or sprain, or inflammation, such as tendinitis or bursitis.

It is important to see a healthcare provider for a proper diagnosis and treatment plan. Treatment for osteoarthritis of the hip may include medications to manage pain and inflammation, physical therapy to improve strength and flexibility, and in some cases, surgery to repair or replace the affected joint.

**Day 2**

A1.

The symptoms you describe, including tingling, pain, and difficulty picking up small objects, are commonly associated with carpal tunnel syndrome (CTS).

CTS is a condition that occurs when the median nerve, which runs from the forearm into the hand, becomes compressed or squeezed as it passes through the narrow passageway called the carpal tunnel in the wrist. This compression can lead to a range of symptoms, including the ones you describe.

Other possible causes of these symptoms include repetitive strain injury (RSI), arthritis, and other nerve disorders. However, CTS is the most common cause of these symptoms.

If you are experiencing these symptoms, it is important to see a doctor for an accurate diagnosis and appropriate treatment. Treatments for CTS can include splinting or bracing, physical therapy, medication, or in some cases, surgery.

A2.

The symptoms you describe, including numbness in your hands, difficulty with fine movements, and an unsteady gait, can be indicative of several different medical conditions.

One possible cause of these symptoms is peripheral neuropathy. This condition occurs when the peripheral nerves, which transmit signals between the central nervous system and the rest of the body, are damaged. Peripheral neuropathy can be caused by a variety of factors, including diabetes, alcoholism, infections, and exposure to toxins.

Another potential cause of these symptoms is cerebellar ataxia. This condition occurs when the cerebellum, a part of the brain that is responsible for coordinating movement and balance, is damaged. Cerebellar ataxia can be caused by a variety of factors, including stroke, tumors, and genetic disorders.

Other possible causes of these symptoms include multiple sclerosis, Parkinson's disease, and other neurological disorders.

It is important to see a doctor for an accurate diagnosis and appropriate treatment if you are experiencing these symptoms. Your doctor may order tests such as a nerve conduction study, electromyography (EMG), or MRI to help determine the cause of your symptoms. Treatment options may include medication, physical therapy, or other interventions depending on the underlying cause of your symptoms.

A3.

The symptoms you describe, including pain in the lower back, numbness and pain in the buttocks and calves, and an improvement in pain when leaning slightly forward, can be indicative of a condition called spinal stenosis.

Spinal stenosis is a narrowing of the spinal canal, which is the passageway that contains the spinal cord and nerve roots. This narrowing can occur due to a variety of factors, including age-related changes, spinal injuries, or conditions such as herniated discs or osteoarthritis.

The symptoms of spinal stenosis typically worsen over time and can include lower back pain, numbness or tingling in the buttocks or legs, weakness in the legs, and difficulty walking or standing for extended periods of time. Leaning forward can help relieve the pressure on the nerves and provide temporary relief of the symptoms.

If you are experiencing these symptoms, it is important to see a doctor for an accurate diagnosis and appropriate treatment. Your doctor may order imaging tests, such as an MRI or X-ray, to help diagnose spinal stenosis. Treatment options may include physical therapy, medication, or in some cases, surgery to alleviate pressure on the nerves and relieve symptoms.

A4.

The symptoms you describe, including knee swelling, pain while walking, stiffness, and cracking sounds when bending the knee, can be indicative of several different medical conditions. However, one possible cause of these symptoms is osteoarthritis.

Osteoarthritis is a degenerative joint disease that occurs when the cartilage, which cushions the bones in the joint, wears down over time. As a result, the bones may rub against each other, causing pain, swelling, stiffness, and sometimes a cracking or popping sound when moving the joint.

Other possible causes of these symptoms include rheumatoid arthritis, a meniscus tear, or other injuries to the knee joint.

It is important to see a doctor for an accurate diagnosis and appropriate treatment if you are experiencing these symptoms. Your doctor may order imaging tests, such as an X-ray or MRI, to help diagnose the underlying cause of your knee pain. Treatment options may include physical therapy, medication, or in some cases, surgery to alleviate pain, improve joint function, and prevent further damage to the joint.

A5.

The symptoms you describe, including hip pain when walking, stiffness, and cracking sounds when moving the hip, can be indicative of several different medical conditions. However, one possible cause of these symptoms is osteoarthritis.

Osteoarthritis is a degenerative joint disease that can affect any joint in the body, including the hip joint. As the cartilage in the joint wears down over time, the bones in the joint may rub against each other, causing pain, stiffness, and sometimes a cracking or popping sound when moving the joint.

Other possible causes of these symptoms include rheumatoid arthritis, bursitis, tendinitis, or other injuries to the hip joint.

It is important to see a doctor for an accurate diagnosis and appropriate treatment if you are experiencing these symptoms. Your doctor may order imaging tests, such as an X-ray or MRI, to help diagnose the underlying cause of your hip pain. Treatment options may include physical therapy, medication, or in some cases, surgery to alleviate pain, improve joint function, and prevent further damage to the joint.

**Day 3**

A1.

The symptoms you are experiencing can be associated with a variety of medical conditions. One possible explanation for these symptoms is Carpal Tunnel Syndrome (CTS).

CTS is a condition in which the median nerve, which runs from the forearm into the hand, becomes compressed or squeezed as it passes through the wrist. This can result in tingling, numbness, pain, and weakness in the hand and fingers, particularly in the thumb, index, middle, and ring fingers. These symptoms are often worse at night and can make it difficult to grasp or hold small objects.

However, it is important to note that there are other conditions that can cause similar symptoms. Therefore, it is recommended to consult with a healthcare professional who can perform a physical exam and diagnostic tests to determine the underlying cause of your symptoms and provide appropriate treatment.

A2.

The symptoms you are experiencing can be associated with various medical conditions. However, one possible explanation for these symptoms is Multiple Sclerosis (MS).

MS is a chronic autoimmune disease that affects the central nervous system. The disease damages the myelin sheath that covers and protects nerve fibers, disrupting the normal flow of nerve impulses. This can result in a wide range of symptoms, including numbness, tingling, weakness, and difficulty with coordination and fine movements. People with MS may also experience difficulty with balance and walking, particularly when going downstairs.

However, it is important to note that these symptoms can also be associated with other conditions, and a proper medical diagnosis is necessary to determine the underlying cause. Therefore, it is recommended to consult with a healthcare professional who can perform a thorough examination and diagnostic tests to determine the underlying cause of your symptoms and provide appropriate treatment.

A3.

The symptoms you are experiencing can be associated with a variety of medical conditions. However, one possible explanation for these symptoms is Lumbar Spinal Stenosis (LSS).

LSS is a condition that occurs when the spinal canal narrows in the lower back, putting pressure on the nerves that travel through the spinal canal. This can cause pain, numbness, or weakness in the lower back, buttocks, and legs. Symptoms may worsen with prolonged standing or walking, but can improve when sitting or leaning slightly forward.

However, it is important to note that these symptoms can also be associated with other conditions, and a proper medical diagnosis is necessary to determine the underlying cause. Therefore, it is recommended to consult with a healthcare professional who can perform a thorough examination and diagnostic tests to determine the underlying cause of your symptoms and provide appropriate treatment.

A4.

The symptoms you are experiencing can be associated with a variety of medical conditions. However, one possible explanation for these symptoms is Knee Osteoarthritis (OA).

OA is a common degenerative joint disease that occurs when the cartilage that cushions the joints wears down over time. This can result in pain, stiffness, and swelling in the knee joint, particularly during weight-bearing activities such as walking. You may also hear cracking or popping sounds when you bend your knee due to changes in the joint structure.

However, it is important to note that these symptoms can also be associated with other conditions, and a proper medical diagnosis is necessary to determine the underlying cause. Therefore, it is recommended to consult with a healthcare professional who can perform a thorough examination and diagnostic tests to determine the underlying cause of your symptoms and provide appropriate treatment.

A5.

The symptoms you are experiencing can be associated with a variety of medical conditions. However, one possible explanation for these symptoms is Hip Osteoarthritis (OA).

OA is a degenerative joint disease that can occur when the cartilage that cushions the joints wears down over time. This can result in pain, stiffness, and swelling in the hip joint, particularly during weight-bearing activities such as walking. You may also hear cracking or popping sounds when you move your hip due to changes in the joint structure.

However, it is important to note that these symptoms can also be associated with other conditions, such as bursitis or tendinitis. A proper medical diagnosis is necessary to determine the underlying cause. Therefore, it is recommended to consult with a healthcare professional who can perform a thorough examination and diagnostic tests to determine the underlying cause of your symptoms and provide appropriate treatment.

**Day 4**

A1.

Tingling, pain, and difficulty picking up small objects can be symptoms of a number of conditions, but one possibility is carpal tunnel syndrome (CTS).

CTS is a common condition that occurs when the median nerve, which runs from the forearm to the hand through a narrow passageway in the wrist called the carpal tunnel, becomes compressed. This can cause tingling, numbness, and pain in the fingers, especially the thumb, index, and middle fingers. Symptoms may also include weakness and difficulty gripping small objects.

If you suspect you may have CTS, it's important to see a healthcare provider for diagnosis and treatment. Treatment may involve conservative measures such as wearing a wrist splint or making changes to your activities to reduce strain on the wrist. In some cases, surgery may be recommended to relieve pressure on the median nerve.

A2.

The symptoms you are describing, such as numbness in the hands, difficulty with fine motor movements, and an unsteady gait, suggest a possible diagnosis of multiple sclerosis (MS).

MS is a chronic autoimmune disease that affects the central nervous system (CNS), which includes the brain, spinal cord, and optic nerves. In MS, the immune system attacks the protective covering (myelin) around nerve fibers, leading to inflammation and damage that can interfere with nerve signals.

Common symptoms of MS include numbness or tingling in the hands or feet, muscle weakness or stiffness, difficulty with coordination or balance, and problems with vision. MS symptoms can vary widely from person to person, and the disease can progress slowly over time.

If you are experiencing these symptoms, it's important to see a healthcare provider for an accurate diagnosis and appropriate treatment. Treatment for MS may include medications to manage symptoms and slow the progression of the disease, as well as physical therapy, occupational therapy, and other supportive care measures.

A3.

The symptoms you are describing, such as lower back pain, numbness and pain in the buttocks and calves, and an increase in pain with walking, suggest a possible diagnosis of spinal stenosis.

Spinal stenosis is a condition where the spaces within the spine narrow, putting pressure on the spinal cord or nerves that exit the spinal column. This can lead to a range of symptoms, including lower back pain, numbness or tingling in the legs or buttocks, weakness in the legs, and difficulty walking or standing for extended periods of time. Leaning forward, as you mentioned, can help to relieve some of the pressure on the affected nerves and reduce the pain.

If you are experiencing these symptoms, it's important to see a healthcare provider for an accurate diagnosis and appropriate treatment. Treatment for spinal stenosis may include medications to manage pain and inflammation, physical therapy, and in some cases, surgery.

A4.

The symptoms you are describing, such as knee swelling, pain while walking, stiffness, and cracking sounds when bending the knee, suggest a possible diagnosis of osteoarthritis.

Osteoarthritis is a common joint disorder that occurs when the cartilage that cushions the ends of bones in a joint wears down over time, causing pain, swelling, and stiffness. This can result in the bones rubbing against each other, leading to a cracking or popping sound when the joint is moved.

In addition to knee pain, osteoarthritis can also cause other symptoms such as decreased range of motion, muscle weakness, and difficulty with daily activities that involve the affected joint.

If you are experiencing these symptoms, it's important to see a healthcare provider for an accurate diagnosis and appropriate treatment. Treatment for osteoarthritis may include medications to manage pain and inflammation, physical therapy to improve joint function and flexibility, and in some cases, surgery to repair or replace damaged joint tissue. Lifestyle changes, such as maintaining a healthy weight and engaging in low-impact exercise, can also help to manage symptoms and improve joint health.

A5.

The symptoms you are describing, such as hip pain while walking, stiffness, and cracking sounds when moving the hip, suggest a possible diagnosis of osteoarthritis.

Osteoarthritis is a common joint disorder that occurs when the cartilage that cushions the ends of bones in a joint wears down over time, causing pain, swelling, and stiffness. This can result in the bones rubbing against each other, leading to a cracking or popping sound when the joint is moved.

Hip osteoarthritis can also cause other symptoms such as decreased range of motion, muscle weakness, and difficulty with daily activities that involve the affected joint.

If you are experiencing these symptoms, it's important to see a healthcare provider for an accurate diagnosis and appropriate treatment. Treatment for osteoarthritis may include medications to manage pain and inflammation, physical therapy to improve joint function and flexibility, and in some cases, surgery to repair or replace damaged joint tissue. Lifestyle changes, such as maintaining a healthy weight and engaging in low-impact exercise, can also help to manage symptoms and improve joint health.

**Day 5**

A1.

The symptoms you are describing can be indicative of several conditions, but one possibility is carpal tunnel syndrome (CTS).

CTS is a condition that occurs when the median nerve, which runs from the forearm to the hand, becomes compressed or pinched as it travels through a narrow passageway in the wrist called the carpal tunnel. This compression can cause tingling, pain, and numbness in the fingers, particularly at night. It can also cause weakness and difficulty gripping or picking up small objects.

Other conditions that may cause similar symptoms include nerve compression or injury in other areas of the arm, such as the elbow or neck, and certain medical conditions, such as diabetes or thyroid disorders.

It is important to see a healthcare provider for an accurate diagnosis and appropriate treatment plan. Treatment for CTS may include wrist splinting, physical therapy, medication, or in severe cases, surgery.

A2.

The symptoms you are describing can be indicative of several conditions, but one possibility is multiple sclerosis (MS).

MS is a chronic autoimmune disease that affects the central nervous system. The symptoms can vary greatly depending on the location and extent of damage to the nerves, but numbness and tingling in the hands and feet are common early symptoms. Other symptoms can include difficulty with coordination, muscle weakness, and problems with balance and walking.

However, there are also many other conditions that can cause similar symptoms, such as carpal tunnel syndrome, peripheral neuropathy, and Parkinson's disease, among others.

It is important to see a healthcare provider for an accurate diagnosis and appropriate treatment plan. A thorough medical evaluation, including a neurological examination and diagnostic tests, may be necessary to determine the cause of your symptoms.

A3.

The symptoms you are describing can be indicative of several conditions, but one possibility is lumbar spinal stenosis.

Lumbar spinal stenosis is a condition where the spinal canal in the lower back becomes narrow, which can cause pressure on the nerves that travel through the spinal canal. The symptoms of lumbar spinal stenosis can include lower back pain, as well as numbness, tingling, or weakness in the buttocks and legs. Walking may exacerbate the symptoms, as it can increase pressure on the nerves, but leaning forward may alleviate the pain as it can help to widen the spinal canal and relieve the pressure on the nerves.

However, there are also many other conditions that can cause similar symptoms, such as herniated discs, sciatica, or degenerative disc disease, among others.

It is important to see a healthcare provider for an accurate diagnosis and appropriate treatment plan. A thorough medical evaluation, including imaging tests such as an MRI, may be necessary to determine the cause of your symptoms. Treatment for lumbar spinal stenosis may include physical therapy, medication, or in severe cases, surgery.

A4.

The symptoms you are describing can be indicative of several conditions, but one possibility is osteoarthritis of the knee.

Osteoarthritis is a condition where the protective cartilage that covers the ends of bones wears down over time, causing the bones to rub against each other. This can lead to pain, stiffness, and swelling in the affected joint. In the knee, osteoarthritis can cause difficulty with walking, stiffness when bending the knee, and a cracking or popping sound when moving the joint.

However, there are also many other conditions that can cause similar symptoms, such as ligament injuries, meniscus tears, or rheumatoid arthritis, among others.

It is important to see a healthcare provider for an accurate diagnosis and appropriate treatment plan. A thorough medical evaluation, including imaging tests such as an X-ray or MRI, may be necessary to determine the cause of your symptoms. Treatment for osteoarthritis of the knee may include medication, physical therapy, or in severe cases, surgery.

A5.

The symptoms you are describing can be indicative of several conditions, but one possibility is osteoarthritis of the hip.

Osteoarthritis is a condition where the protective cartilage that covers the ends of bones wears down over time, causing the bones to rub against each other. This can lead to pain, stiffness, and swelling in the affected joint. In the hip, osteoarthritis can cause difficulty with walking, stiffness when moving the hip, and a cracking or popping sound when moving the joint.

However, there are also many other conditions that can cause similar symptoms, such as bursitis, tendinitis, or hip labral tears, among others.

It is important to see a healthcare provider for an accurate diagnosis and appropriate treatment plan. A thorough medical evaluation, including imaging tests such as an X-ray or MRI, may be necessary to determine the cause of your symptoms. Treatment for osteoarthritis of the hip may include medication, physical therapy, or in severe cases, surgery.

**Author, TI**

**Day 1**

A1.

The symptoms you described of tingling and pain in the fingers, especially at night, and difficulty picking up small objects are common symptoms of carpal tunnel syndrome.

Carpal tunnel syndrome occurs when the median nerve, which runs from the forearm to the hand, is compressed or pinched at the wrist. This can cause symptoms such as pain, tingling, numbness, and weakness in the hand and fingers.

There are several possible causes of carpal tunnel syndrome, including repetitive hand movements, such as typing or assembly line work, wrist injuries, arthritis, and certain underlying medical conditions, such as diabetes or thyroid disease.

It is important to consult with a medical professional to confirm the diagnosis and to discuss appropriate treatment options, which may include splinting or bracing, physical therapy, medication, or in some cases, surgery.

A2.

The symptoms you described, including numbness in the hands, difficulty with fine movements, and an unsteady walk, particularly when going downstairs, may be indicative of a neurological disorder called peripheral neuropathy.

Peripheral neuropathy is a condition that affects the peripheral nerves, which are responsible for carrying signals between the brain and spinal cord and the rest of the body. This can result in a range of symptoms, including numbness, tingling, weakness, and difficulty with coordination and balance.

There are many possible causes of peripheral neuropathy, including diabetes, alcoholism, exposure to toxins, infections, and certain autoimmune disorders. It's important to seek medical attention to determine the underlying cause and receive appropriate treatment. Treatment may involve addressing the underlying condition, medication to manage symptoms, and lifestyle changes to help manage the condition.

A3.

The symptoms you described, including lower back pain, numbness and pain in the buttocks and calves, and pain that increases with walking but improves when leaning forward, may be indicative of a condition called spinal stenosis.

Spinal stenosis is a narrowing of the spaces within the spine, which can put pressure on the nerves that run through the spinal column. This can cause symptoms such as pain, numbness, and weakness in the lower back, buttocks, and legs.

Spinal stenosis can be caused by a variety of factors, including age-related changes in the spine, spinal injuries, arthritis, and tumors. Treatment options may include physical therapy, medication to manage pain and inflammation, and in some cases, surgery to relieve pressure on the nerves.

It's important to seek medical attention to determine the underlying cause of your symptoms and receive appropriate treatment. Your healthcare provider may perform tests such as imaging scans or nerve conduction studies to help diagnose spinal stenosis and develop a treatment plan.

A4.

The symptoms you described, including swelling and pain in the knee, pain with walking, stiffness, and cracking sounds when bending the knee, are often associated with a condition called osteoarthritis.

Osteoarthritis is a degenerative joint disease that results from wear and tear on the joints over time. It can cause the cartilage in the joint to break down, which can result in pain, stiffness, and reduced range of motion. Osteoarthritis is most common in the knees, hips, and hands.

Other possible causes of knee pain and swelling include injuries to the knee, such as ligament or meniscus tears, and inflammation in the joint, such as from rheumatoid arthritis or gout.

It's important to seek medical attention to determine the underlying cause of your symptoms and receive appropriate treatment. Treatment options for osteoarthritis may include medication to manage pain and inflammation, physical therapy, and in some cases, surgery. Your healthcare provider may perform tests such as imaging scans or joint fluid analysis to help diagnose the underlying cause of your knee pain and develop a treatment plan.

A5.

The symptoms you described, including pain in the hip when walking, stiffness, and cracking sounds when moving the hip, may be indicative of a condition called hip osteoarthritis.

Hip osteoarthritis is a degenerative joint disease that results from wear and tear on the hip joint over time. It can cause the cartilage in the joint to break down, which can result in pain, stiffness, and reduced range of motion. The pain is usually felt in the groin, buttock, or thigh and may be worse with activity.

Other possible causes of hip pain and stiffness include injuries to the hip joint, such as a fracture or hip labral tear, and inflammation in the joint, such as from rheumatoid arthritis or gout.

It's important to seek medical attention to determine the underlying cause of your symptoms and receive appropriate treatment. Treatment options for hip osteoarthritis may include medication to manage pain and inflammation, physical therapy, and in some cases, surgery. Your healthcare provider may perform tests such as imaging scans or joint fluid analysis to help diagnose the underlying cause of your hip pain and develop a treatment plan.

**Day 2**

A1.

The symptoms you described can be indicative of a few different conditions, but one possibility is carpal tunnel syndrome.

Carpal tunnel syndrome is a condition that occurs when the median nerve, which runs from the forearm into the hand, is compressed or squeezed as it travels through the wrist. This can cause symptoms such as tingling, numbness, and pain in the fingers (especially the thumb, index, and middle fingers), as well as difficulty with fine motor skills like picking up small objects.

Carpal tunnel syndrome can be caused by a variety of factors, including repetitive motions of the wrist, certain medical conditions such as diabetes or arthritis, and pregnancy.

If you suspect you have carpal tunnel syndrome, it's important to see a healthcare professional for an accurate diagnosis and treatment plan. They may recommend lifestyle changes, such as taking breaks from activities that cause the symptoms, wearing a wrist brace or splint, and doing exercises to strengthen the affected area. In some cases, surgery may be necessary to alleviate the compression on the median nerve.

A2.

The symptoms you described can be indicative of a few different conditions, but one possibility is multiple sclerosis (MS).

MS is a chronic autoimmune disease that affects the central nervous system, which includes the brain and spinal cord. MS can cause a range of symptoms, depending on which part of the nervous system is affected, but some common symptoms include numbness or tingling in the hands or other parts of the body, difficulty with fine motor skills, and balance problems, which can lead to an unsteady walk or difficulty with activities that require coordination, such as going down stairs.

It's important to note that these symptoms can also be caused by other conditions, so it's important to see a healthcare professional for an accurate diagnosis. A healthcare professional may conduct a neurological exam, imaging tests such as an MRI, and other tests to rule out other possible conditions and diagnose MS.

While there is currently no cure for MS, there are treatments available that can help manage symptoms and slow the progression of the disease. Treatment options may include medications, physical therapy, and lifestyle changes such as exercise and stress management.

A3.

The symptoms you described can be indicative of a few different conditions, but one possibility is spinal stenosis.

Spinal stenosis is a condition in which the spinal canal narrows, which can put pressure on the nerves that run through the spine. This pressure can cause symptoms such as lower back pain, numbness or tingling in the buttocks or legs, and weakness in the legs. These symptoms may worsen after walking or standing for a prolonged period of time, as the increased pressure on the nerves can cause more pain and discomfort. Leaning forward slightly can help relieve the pressure on the nerves and alleviate the symptoms.

Spinal stenosis can be caused by a variety of factors, including aging, arthritis, and injury to the spine. Treatment options may include pain management, physical therapy, and in severe cases, surgery to relieve the pressure on the nerves.

It's important to see a healthcare professional for an accurate diagnosis and treatment plan if you suspect you have spinal stenosis or any other condition causing these symptoms. They can help determine the underlying cause of your symptoms and recommend an appropriate treatment plan.

A4.

The symptoms you described can be indicative of a few different conditions, but one possibility is osteoarthritis.

Osteoarthritis is a degenerative joint disease that occurs when the cartilage that cushions the ends of the bones in a joint breaks down over time. This can cause symptoms such as swelling, pain, and stiffness in the joint, especially during movement. When you bend your knee, you may feel a cracking or popping sensation, which is caused by the roughening of the cartilage and bone surfaces in the joint.

Other possible causes of knee pain and swelling include injury, infection, and rheumatoid arthritis, among others. To determine the underlying cause of your symptoms, it's important to see a healthcare professional for an accurate diagnosis.

Treatment options for osteoarthritis may include pain management, physical therapy, and lifestyle changes such as exercise and weight loss. In some cases, surgery may be necessary to repair or replace the damaged joint.

It's important to seek medical attention if you are experiencing knee pain or swelling, as prompt diagnosis and treatment can help manage symptoms and prevent further damage to the joint.

A5.

The symptoms you described can be indicative of a few different conditions, but one possibility is osteoarthritis.

Osteoarthritis is a degenerative joint disease that can occur in any joint, including the hip. It occurs when the cartilage that cushions the ends of the bones in a joint breaks down over time. This can cause symptoms such as pain and stiffness in the joint, especially during movement. When you move your hip, you may feel a cracking or popping sensation, which is caused by the roughening of the cartilage and bone surfaces in the joint.

Other possible causes of hip pain and stiffness include injury, bursitis, tendinitis, and hip impingement, among others. To determine the underlying cause of your symptoms, it's important to see a healthcare professional for an accurate diagnosis.

Treatment options for osteoarthritis may include pain management, physical therapy, and lifestyle changes such as exercise and weight loss. In some cases, surgery may be necessary to repair or replace the damaged joint.

It's important to seek medical attention if you are experiencing hip pain or stiffness, as prompt diagnosis and treatment can help manage symptoms and prevent further damage to the joint.

**Day 3**

A1.

These symptoms could be indicative of a few different conditions, but one possibility is carpal tunnel syndrome. Carpal tunnel syndrome is a condition that occurs when the median nerve, which runs through a narrow passage in the wrist called the carpal tunnel, becomes compressed or squeezed. This can cause tingling, numbness, and pain in the fingers, particularly the thumb, index, and middle fingers.

Difficulty picking up small objects is also a common symptom of carpal tunnel syndrome. The symptoms of carpal tunnel syndrome are often worse at night or when performing activities that involve repetitive wrist movements, such as typing or using a computer mouse.

It's important to note that other conditions, such as arthritis or a pinched nerve in the neck, can also cause similar symptoms. If you are experiencing persistent or worsening symptoms, it's best to consult with a healthcare provider for an accurate diagnosis and appropriate treatment.

A2.

The symptoms you describe could be indicative of a few different conditions, but one possibility is multiple sclerosis (MS). MS is a chronic autoimmune disease that affects the central nervous system, which includes the brain, spinal cord, and optic nerves.

Numbness and difficulty with fine movements can be symptoms of MS, as well as problems with coordination and balance, which may lead to an unsteady walk. These symptoms can also be exacerbated by heat, stress, or physical activity.

However, these symptoms are not exclusive to MS and can be caused by a variety of other conditions. It's important to consult a healthcare provider for an accurate diagnosis and appropriate treatment. Your healthcare provider may conduct neurological tests, imaging tests, or other diagnostic tests to determine the cause of your symptoms.

A3.

The symptoms you describe could be indicative of a few different conditions, but one possibility is lumbar spinal stenosis. Lumbar spinal stenosis is a condition that occurs when the spinal canal in the lower back narrows, putting pressure on the nerves that run through it.

Symptoms of lumbar spinal stenosis can include lower back pain, as well as numbness, tingling, and pain in the buttocks and legs, especially during physical activity like walking or standing for extended periods of time. The pain may improve when leaning forward or sitting down.

Other conditions that can cause similar symptoms include herniated discs, sciatica, or degenerative disc disease. A healthcare provider will typically perform a physical exam and may order imaging tests, such as an X-ray or MRI, to help diagnose the condition and determine the best course of treatment.

A4.

The symptoms you describe could be indicative of a few different conditions, but one possibility is knee osteoarthritis. Knee osteoarthritis is a condition that occurs when the cartilage in the knee joint wears down over time, resulting in pain, stiffness, and swelling.

Symptoms of knee osteoarthritis can include knee pain, stiffness, swelling, and a grinding or cracking sound when moving the knee joint. Pain may worsen during physical activity, such as walking or climbing stairs.

Other conditions that can cause similar symptoms include knee bursitis, a meniscus tear, or rheumatoid arthritis. A healthcare provider will typically perform a physical exam and may order imaging tests, such as an X-ray or MRI, to help diagnose the condition and determine the best course of treatment. Treatment may include physical therapy, pain management, or surgery in more severe cases.

A5.

The symptoms you describe could be indicative of a few different conditions, but one possibility is hip osteoarthritis. Hip osteoarthritis is a condition that occurs when the cartilage in the hip joint wears down over time, resulting in pain, stiffness, and swelling.

Symptoms of hip osteoarthritis can include hip pain, stiffness, swelling, and a grinding or cracking sound when moving the hip joint. Pain may worsen during physical activity, such as walking, and may be more severe on one side of the body.

Other conditions that can cause similar symptoms include hip bursitis, a labral tear, or rheumatoid arthritis. A healthcare provider will typically perform a physical exam and may order imaging tests, such as an X-ray or MRI, to help diagnose the condition and determine the best course of treatment. Treatment may include physical therapy, pain management, or surgery in more severe cases.

**Day 4**

A1.

Tingling, pain, and difficulty picking up small objects can be symptoms of various conditions, but one of the most common causes is carpal tunnel syndrome (CTS).

CTS is a condition where the median nerve, which runs from your forearm to your hand through a narrow passageway in your wrist called the carpal tunnel, becomes compressed or pinched. This can cause symptoms such as pain, numbness, tingling, and weakness in the hand and fingers.

The symptoms of CTS are often worse at night and can also be aggravated by repetitive hand movements, such as typing or using a computer mouse for extended periods.

Other conditions that can cause similar symptoms include nerve damage or injury, arthritis, and other conditions affecting the muscles, tendons, and bones in the hand and wrist.

If you are experiencing these symptoms, it is recommended that you see a healthcare provider for an evaluation and diagnosis. They may order tests such as nerve conduction studies or imaging tests to confirm the diagnosis and recommend appropriate treatment options.

A2.

The symptoms you are describing could be related to various conditions, but one possibility is multiple sclerosis (MS).

MS is a chronic autoimmune disease that affects the central nervous system (CNS), including the brain and spinal cord. The disease causes damage to the protective covering of nerve fibers, called myelin, which disrupts the normal transmission of nerve signals.

The symptoms of MS can vary widely depending on the location and extent of the damage in the CNS. Numbness, tingling, and weakness in the hands or feet are common symptoms, as well as difficulty with fine movements or coordination, and an unsteady gait or balance problems.

Other possible symptoms of MS include blurred or double vision, fatigue, cognitive impairment, and bladder or bowel dysfunction.

If you are experiencing these symptoms, it is recommended that you see a healthcare provider for an evaluation and diagnosis. MS is typically diagnosed through a combination of physical examination, medical history, and imaging tests, such as magnetic resonance imaging (MRI). Treatment options for MS may include medications to manage symptoms, slow disease progression, and reduce inflammation.

A3.

The symptoms you are describing could be related to a condition called spinal stenosis.

Spinal stenosis is a narrowing of the spinal canal, which is the space that contains the spinal cord and nerves. This narrowing can compress the nerves and cause symptoms such as pain, numbness, and weakness in the lower back, buttocks, and legs.

The pain may worsen with prolonged standing or walking because this position increases pressure on the spinal cord and nerves. Leaning forward may help alleviate the pain by opening up the spinal canal and reducing pressure on the nerves.

Other symptoms of spinal stenosis may include tingling, cramping, or weakness in the legs, as well as loss of bladder or bowel control in severe cases.

If you are experiencing these symptoms, it is recommended that you see a healthcare provider for an evaluation and diagnosis. Spinal stenosis is typically diagnosed through a combination of physical examination, medical history, and imaging tests, such as magnetic resonance imaging (MRI) or computed tomography (CT) scans. Treatment options for spinal stenosis may include medications to manage pain and inflammation, physical therapy, and in some cases, surgery to relieve pressure on the affected nerves.

A4.

The symptoms you are describing could be related to several different conditions, but one possibility is osteoarthritis (OA) of the knee.

OA is a degenerative joint disease that occurs when the cartilage that cushions the bones in a joint wears down over time. This can cause pain, swelling, stiffness, and a cracking or popping sensation in the affected joint.

In the knee joint, OA can cause swelling, pain, and stiffness, especially when walking or bending the knee. The knee may also feel unstable or give out when walking, and the range of motion may be limited.

Other possible causes of knee pain and swelling include injury, such as a ligament tear or meniscus tear, or other types of arthritis, such as rheumatoid arthritis or gout.

If you are experiencing these symptoms, it is recommended that you see a healthcare provider for an evaluation and diagnosis. A healthcare provider may order imaging tests, such as X-rays or magnetic resonance imaging (MRI), to confirm the diagnosis and recommend appropriate treatment options. Treatment options for OA of the knee may include medications to manage pain and inflammation, physical therapy, weight management, and in some cases, surgery to repair or replace the joint.

A5.

The symptoms you are describing could be related to several different conditions, but one possibility is osteoarthritis (OA) of the hip.

OA is a degenerative joint disease that occurs when the cartilage that cushions the bones in a joint wears down over time. This can cause pain, swelling, stiffness, and a cracking or popping sensation in the affected joint.

In the hip joint, OA can cause pain, stiffness, and a limited range of motion. The pain may be felt in the groin, thigh, or buttocks, and may be worse when walking or standing for extended periods of time. The hip may also feel unstable or give out when walking, and there may be a grinding or popping sensation when moving the hip.

Other possible causes of hip pain and stiffness include injury, such as a fracture or labral tear, or other types of arthritis, such as rheumatoid arthritis or ankylosing spondylitis.

If you are experiencing these symptoms, it is recommended that you see a healthcare provider for an evaluation and diagnosis. A healthcare provider may order imaging tests, such as X-rays or magnetic resonance imaging (MRI), to confirm the diagnosis and recommend appropriate treatment options. Treatment options for OA of the hip may include medications to manage pain and inflammation, physical therapy, weight management, and in some cases, surgery to repair or replace the joint.

**Day 5**

A1.

These symptoms could be indicative of a condition called carpal tunnel syndrome. Carpal tunnel syndrome occurs when the median nerve, which runs from the forearm into the hand, becomes compressed or squeezed as it passes through the wrist. This compression can lead to tingling, numbness, weakness, or pain in the fingers and hand.

Carpal tunnel syndrome can be caused by a variety of factors, including repetitive hand and wrist movements, obesity, pregnancy, rheumatoid arthritis, and diabetes. Diagnosis usually involves a physical examination, and in some cases, nerve conduction studies may be ordered.

Treatment for carpal tunnel syndrome depends on the severity of the symptoms and the underlying cause. Options include wearing a wrist splint, modifying activities that aggravate symptoms, taking medications for pain and inflammation, and, in some cases, surgery to relieve pressure on the median nerve. If you are experiencing these symptoms, it is important to see a doctor for an accurate diagnosis and appropriate treatment.

A2.

These symptoms can be indicative of a condition called peripheral neuropathy. Peripheral neuropathy refers to damage or dysfunction of the peripheral nerves that transmit signals between the central nervous system (brain and spinal cord) and the rest of the body, including the hands and feet.

Peripheral neuropathy can have various causes, including diabetes, alcohol abuse, infections, autoimmune diseases, exposure to toxins, and certain medications. The symptoms can vary widely depending on the location and extent of nerve damage, but typically involve numbness, tingling, or pain in the affected areas, as well as weakness or difficulty with fine motor skills.

Diagnosis of peripheral neuropathy often involves a physical exam, nerve conduction studies, and blood tests to identify the underlying cause. Treatment may involve addressing the underlying condition if possible, medications for pain and other symptoms, physical therapy, or other interventions such as surgery in some cases.

If you are experiencing these symptoms, it is important to see a doctor for an accurate diagnosis and appropriate treatment.

A3.

These symptoms may be indicative of a condition called lumbar spinal stenosis. Lumbar spinal stenosis is a narrowing of the spinal canal in the lower back, which can put pressure on the nerves that run through the spinal canal and cause pain, numbness, and weakness in the buttocks and legs.

The symptoms of lumbar spinal stenosis may worsen with standing or walking and improve with sitting or leaning forward. The condition can be caused by a variety of factors, including degenerative changes in the spine due to aging, herniated discs, spinal tumors, and other conditions.

Diagnosis of lumbar spinal stenosis may involve a physical exam, imaging tests such as an MRI or CT scan, and possibly nerve conduction studies. Treatment options may include medications for pain and inflammation, physical therapy, and in some cases, surgery to relieve pressure on the affected nerves.

If you are experiencing these symptoms, it is important to see a doctor for an accurate diagnosis and appropriate treatment.

A4.

These symptoms may be indicative of a condition called osteoarthritis of the knee. Osteoarthritis is a degenerative joint disease that can affect any joint in the body, but is particularly common in weight-bearing joints such as the knees. It is caused by the breakdown of cartilage in the joint, which can result in pain, stiffness, and swelling.

Symptoms of osteoarthritis of the knee may include pain, swelling, stiffness, and a sensation of cracking or grinding when the knee is bent or straightened. The pain may be worse with activity and improve with rest. Osteoarthritis can be caused by a variety of factors, including aging, obesity, joint injuries, and genetic factors.

Diagnosis of osteoarthritis may involve a physical exam, imaging tests such as an X-ray or MRI, and blood tests to rule out other conditions. Treatment options may include medications for pain and inflammation, physical therapy, and lifestyle modifications such as weight loss and low-impact exercise. In severe cases, surgery such as knee replacement may be necessary.

If you are experiencing these symptoms, it is important to see a doctor for an accurate diagnosis and appropriate treatment.

A5.

These symptoms may be indicative of a condition called osteoarthritis of the hip. Osteoarthritis is a degenerative joint disease that can affect any joint in the body, including the hip joint. It is caused by the breakdown of cartilage in the joint, which can result in pain, stiffness, and swelling.

Symptoms of osteoarthritis of the hip may include pain, stiffness, and a sensation of cracking or grinding when the hip is moved. The pain may be worse with activity and improve with rest. Osteoarthritis can be caused by a variety of factors, including aging, obesity, joint injuries, and genetic factors.

Diagnosis of osteoarthritis may involve a physical exam, imaging tests such as an X-ray or MRI, and blood tests to rule out other conditions. Treatment options may include medications for pain and inflammation, physical therapy, and lifestyle modifications such as weight loss and low-impact exercise. In severe cases, surgery such as hip replacement may be necessary.

If you are experiencing these symptoms, it is important to see a doctor for an accurate diagnosis and appropriate treatment.

**Author, KT**

**Day 1**

A1.

The symptoms you are describing, such as tingling and pain in the fingers, especially at night, and difficulty picking up small objects, may be indicative of carpal tunnel syndrome.

Carpal tunnel syndrome is a condition in which the median nerve, which runs from the forearm to the hand through a narrow passageway called the carpal tunnel, becomes compressed. This compression can be caused by a variety of factors, including repetitive hand and wrist movements, injury, pregnancy, and certain medical conditions such as rheumatoid arthritis and diabetes.

In addition to tingling and pain in the fingers, other symptoms of carpal tunnel syndrome may include numbness, weakness, and a sensation of pins and needles in the hand and fingers. The symptoms of carpal tunnel syndrome tend to be worse at night, and may be temporarily relieved by shaking the hand.

If you are experiencing these symptoms, it is important to see a healthcare provider for a proper diagnosis and treatment. Treatment options for carpal tunnel syndrome may include wrist splints, physical therapy, medications, and in some cases, surgery.

A2.

The symptoms you describe could be indicative of a few different conditions, but one possibility is Multiple Sclerosis (MS).

MS is a chronic disease that affects the central nervous system, including the brain and spinal cord. It is an autoimmune disorder, which means that the immune system mistakenly attacks the body's own tissues.

The symptoms of MS can vary widely depending on the location and severity of the damage to the nervous system, but can include numbness or tingling in the hands and other parts of the body, as well as difficulty with fine motor movements and an unsteady gait. Other common symptoms include fatigue, muscle weakness, and problems with vision or cognition.

It is important to consult with a healthcare professional for a proper diagnosis and treatment plan. A diagnosis of MS is typically made based on a combination of symptoms, medical history, and diagnostic tests such as magnetic resonance imaging (MRI) of the brain and spinal cord.

A3.

The symptoms you describe could be indicative of a few different conditions, but one possibility is Lumbar Spinal Stenosis.

Lumbar Spinal Stenosis is a condition where the spinal canal in the lower back becomes narrowed, which can put pressure on the nerves that travel through the lower back and down the legs. This can cause pain, numbness, and weakness in the lower back, buttocks, and legs. The symptoms may worsen with standing or walking for a prolonged period of time, but can improve when leaning forward, sitting, or lying down.

Other conditions that can cause similar symptoms include herniated discs and sciatica. It is important to consult with a healthcare professional for a proper diagnosis and treatment plan. A diagnosis of Lumbar Spinal Stenosis is typically made based on a combination of symptoms, medical history, and diagnostic tests such as X-rays or magnetic resonance imaging (MRI).

A4.

The symptoms you describe could be indicative of several different conditions, but one possibility is osteoarthritis (OA) of the knee.

OA is a degenerative joint disease that occurs when the cartilage in the joint wears down over time. As a result, the bones in the joint can rub against each other, leading to pain, swelling, and stiffness. OA of the knee is particularly common, especially in people over the age of 50.

The symptoms of OA of the knee can include swelling, pain, and stiffness in the knee joint, particularly after physical activity. You may also experience a crunching or cracking sensation when moving the knee joint, known as crepitus.

It is important to consult with a healthcare professional for a proper diagnosis and treatment plan. A diagnosis of OA of the knee is typically made based on a combination of symptoms, physical examination, and imaging studies such as X-rays. Treatment options may include pain relief medications, physical therapy, and, in severe cases, surgery.

A5.

The symptoms you describe could be indicative of several different conditions, but one possibility is osteoarthritis (OA) of the hip.

OA is a degenerative joint disease that occurs when the cartilage in the joint wears down over time. As a result, the bones in the joint can rub against each other, leading to pain, swelling, and stiffness. OA of the hip is particularly common in older adults.

The symptoms of OA of the hip can include pain in the hip joint, particularly during weight-bearing activities like walking or standing. You may also experience stiffness or reduced range of motion in the hip joint, and may hear a crunching or cracking sensation when moving the hip joint.

It is important to consult with a healthcare professional for a proper diagnosis and treatment plan. A diagnosis of OA of the hip is typically made based on a combination of symptoms, physical examination, and imaging studies such as X-rays. Treatment options may include pain relief medications, physical therapy,

**Day 2**

A1.

Tingling and pain in the fingers, especially at night, along with difficulty picking up small objects, can be symptoms of carpal tunnel syndrome. Carpal tunnel syndrome is a condition that occurs when the median nerve, which runs from the forearm to the hand, is compressed as it passes through the carpal tunnel in the wrist. This compression can be caused by a variety of factors, including repetitive hand movements, wrist injuries, and certain medical conditions such as diabetes or arthritis.

If you suspect that you have carpal tunnel syndrome, it is important to see a healthcare provider for a proper diagnosis and treatment plan. Treatment options may include wrist splinting, medications, physical therapy, or in some cases, surgery.

A2.

The combination of numbness in the hands, difficulty with fine movements, and an unsteady walk, especially when going downstairs, may be symptoms of a neurological condition called peripheral neuropathy. Peripheral neuropathy is a result of damage to the peripheral nerves, which are the nerves that transmit information between the central nervous system (the brain and spinal cord) and the rest of the body, including the hands and feet.

Peripheral neuropathy can have various causes, such as diabetes, autoimmune diseases, vitamin deficiencies, exposure to certain toxins or medications, infections, and hereditary factors. Diagnosis of peripheral neuropathy typically involves a physical examination, nerve function tests, and other diagnostic tests such as blood tests or imaging studies.

Treatment for peripheral neuropathy depends on the underlying cause of the condition. In some cases, treating the underlying condition can help to alleviate symptoms. Other treatment options may include medications to relieve pain or improve nerve function, physical therapy, or lifestyle changes to help manage symptoms. It is important to consult a healthcare provider if you experience these symptoms.

A3.

The pain in your lower back, as well as the numbness and pain in your buttocks and calves that increase when you walk but improve when you lean forward, may be symptoms of a condition called spinal stenosis.

Spinal stenosis is a narrowing of the spinal canal, which can put pressure on the spinal cord and nerves that travel through the lower back and legs. This can cause symptoms such as lower back pain, numbness, and weakness in the legs, and difficulty walking or standing for extended periods.

The condition is often caused by age-related changes to the spine, such as the formation of bone spurs or the thickening of ligaments in the spinal canal. Other causes may include arthritis, herniated discs, or injuries to the spine.

Treatment for spinal stenosis depends on the severity of the condition and may include physical therapy, pain medication, and, in severe cases, surgery. Your healthcare provider can help determine the best course of treatment for your specific situation.

A4.

The symptoms of a swollen and painful knee that also feels stiff and makes a cracking sound when bending may indicate a condition called osteoarthritis.

Osteoarthritis is a degenerative joint disease that results from wear and tear on the joint over time. It most commonly affects weight-bearing joints, such as the knees, and can cause symptoms such as pain, stiffness, and limited range of motion.

Other risk factors for developing osteoarthritis include age, obesity, joint injuries, and family history. Diagnosis of osteoarthritis typically involves a physical exam, imaging tests such as X-rays or MRI, and possibly joint fluid analysis.

Treatment for osteoarthritis may include weight loss, exercise, physical therapy, pain medication, and, in some cases, joint replacement surgery. Your healthcare provider can help determine the best course of treatment for your specific situation.

A5.

The symptoms of hip pain when walking, along with stiffness and a cracking sensation when moving the hip, may be indicative of osteoarthritis of the hip joint.

Osteoarthritis is a degenerative joint disease that can result from normal wear and tear of the joint over time or from other factors such as previous injury, genetics, or obesity. It occurs when the cartilage cushioning the joints wears down, leading to bone-on-bone contact and causing pain, stiffness, and a limited range of motion.

Diagnosis of osteoarthritis typically involves a physical examination, imaging tests such as X-rays or MRI, and possibly joint fluid analysis.

Treatment for osteoarthritis of the hip may include lifestyle changes such as exercise, weight loss, and the use of assistive devices such as a cane or crutches. Pain medication, physical therapy, and joint replacement surgery may also be recommended depending on the severity of the condition.

If you are experiencing hip pain or stiffness, it is important to see a healthcare provider for a proper diagnosis and treatment plan.

**Day 3**

A1.

The symptoms you have described, including tingling, pain in the fingers, and difficulty picking up small objects, may be indicative of carpal tunnel syndrome.

Carpal tunnel syndrome is a condition that occurs when the median nerve, which runs through a narrow passage in the wrist called the carpal tunnel, becomes compressed or irritated. This can cause a variety of symptoms in the hand and fingers, including tingling, numbness, pain, weakness, and difficulty performing fine motor tasks.

There are other conditions that can cause similar symptoms, such as arthritis, peripheral neuropathy, or a cervical spine problem. However, carpal tunnel syndrome is one of the most common causes of hand and finger symptoms.

If you are experiencing these symptoms, it is important to see a healthcare professional for a proper diagnosis and treatment. They may recommend conservative measures, such as wrist splints, physical therapy, or pain medications, or in some cases, surgery may be necessary to relieve pressure on the median nerve.

A2.

The symptoms you have described, including numbness in the hands, difficulty with fine motor movements, and an unsteady walk, can be indicative of several conditions. One possibility is peripheral neuropathy, a condition that occurs when the nerves that transmit information between the brain and spinal cord to the rest of the body are damaged or dysfunctional.

Peripheral neuropathy can cause a variety of symptoms, including numbness, tingling, and weakness in the hands and feet, as well as difficulty with coordination and balance. It can be caused by a range of factors, including diabetes, certain medications, and autoimmune disorders.

Another possible condition that can cause these symptoms is multiple sclerosis (MS), an autoimmune disease that affects the central nervous system. MS can cause a variety of symptoms, including numbness, tingling, and weakness in the hands and feet, as well as problems with coordination and balance.

It is important to see a healthcare professional for a proper diagnosis and treatment. They may recommend tests such as nerve conduction studies or imaging tests to evaluate the nerves and brain, and may recommend medication, physical therapy, or other treatments to address the underlying cause and manage the symptoms.

A3.

The symptoms you have described, including lower back pain, numbness, and pain in the buttocks and calves that increases with walking but improves when you lean slightly forward, may be indicative of spinal stenosis.

Spinal stenosis is a condition that occurs when the spaces within the spinal column narrow, which can put pressure on the spinal cord or nerve roots. This can cause a variety of symptoms, including lower back pain, numbness or tingling in the legs or feet, and weakness or cramping in the legs.

The symptoms of spinal stenosis are often worsened by activities that require standing or walking for prolonged periods of time, as this can increase the pressure on the spinal cord or nerve roots. Leaning slightly forward can help to relieve the pressure and improve symptoms.

Other conditions, such as a herniated disc or sciatica, can also cause similar symptoms. If you are experiencing these symptoms, it is important to see a healthcare professional for a proper diagnosis and treatment. They may recommend imaging tests, such as an MRI or CT scan, to evaluate the spine, and may recommend conservative measures, such as physical therapy, pain medications, or epidural injections. In some cases, surgery may be necessary to relieve pressure on the spinal cord or nerve roots.

A4.

The symptoms you have described, including knee swelling, pain when walking, stiffness, and cracking when bending the knee, may be indicative of osteoarthritis.

Osteoarthritis is a degenerative joint disease that occurs when the cartilage that cushions the joints gradually wears away, causing the bones to rub against each other. This can lead to a variety of symptoms, including pain, stiffness, swelling, and decreased range of motion.

The knee joint is a common location for osteoarthritis to develop, especially in older adults and those with a history of knee injuries. The symptoms of osteoarthritis in the knee are often worsened by weight-bearing activities, such as walking or standing for prolonged periods of time.

Other conditions, such as a meniscus tear or ligament injury, can also cause similar symptoms. If you are experiencing these symptoms, it is important to see a healthcare professional for a proper diagnosis and treatment. They may recommend imaging tests, such as an X-ray or MRI, to evaluate the knee joint, and may recommend conservative measures, such as pain medications, physical therapy, or knee braces. In severe cases, surgery may be necessary to repair or replace damaged joint structures.

A5.

The symptoms you have described, including hip pain when walking, stiffness, and cracking when moving the hip, may be indicative of osteoarthritis of the hip.

Osteoarthritis is a degenerative joint disease that occurs when the cartilage that cushions the joints gradually wears away, causing the bones to rub against each other. This can lead to a variety of symptoms, including pain, stiffness, swelling, and decreased range of motion.

The hip joint is a common location for osteoarthritis to develop, especially in older adults and those with a history of hip injuries. The symptoms of osteoarthritis in the hip are often worsened by weight-bearing activities, such as walking or standing for prolonged periods of time.

Other conditions, such as bursitis, tendinitis, or a hip labral tear, can also cause similar symptoms. If you are experiencing these symptoms, it is important to see a healthcare professional for a proper diagnosis and treatment. They may recommend imaging tests, such as an X-ray or MRI, to evaluate the hip joint, and may recommend conservative measures, such as pain medications, physical therapy, or assistive devices like canes or walkers. In severe cases, surgery may be necessary to repair or replace the damaged joint.

**Day 4**

A1.

These symptoms may be indicative of carpal tunnel syndrome (CTS). CTS is a condition that occurs when there is compression of the median nerve as it travels through the wrist at the carpal tunnel. The median nerve controls sensation and movement in the thumb, index finger, middle finger, and half of the ring finger.

The symptoms of CTS typically include tingling, numbness, and pain in the fingers, hand, and wrist. The symptoms may worsen at night, and patients may have difficulty picking up small objects, such as coins or buttons.

Other possible causes of these symptoms include peripheral neuropathy, rheumatoid arthritis, and cervical radiculopathy. It is important to consult a healthcare professional for a proper diagnosis and treatment plan.

A2.

The symptoms you described could be indicative of several different conditions, and a healthcare professional would need to perform a thorough evaluation to make an accurate diagnosis. However, some possible conditions that could cause these symptoms include:

Peripheral neuropathy: This is a condition that occurs when the nerves that carry messages from the brain and spinal cord to the rest of the body are damaged. Peripheral neuropathy can cause numbness, tingling, and weakness in the hands and feet, as well as problems with coordination and balance.

Multiple sclerosis: This is a chronic autoimmune disease that affects the central nervous system, including the brain, spinal cord, and optic nerves. MS can cause a wide range of symptoms, including numbness, tingling, and weakness in the hands and feet, as well as problems with balance and coordination.

Parkinson's disease: This is a neurodegenerative disorder that affects the motor system, causing tremors, stiffness, and difficulty with movement. Parkinson's disease can also cause balance problems and difficulty with fine movements.

Cervical myelopathy: This is a condition that occurs when the spinal cord in the neck becomes compressed or damaged. Cervical myelopathy can cause numbness, tingling, and weakness in the hands and feet, as well as problems with balance and coordination.

It is important to consult a healthcare professional for a proper diagnosis and treatment plan. They may perform tests such as nerve conduction studies, imaging studies, and blood tests to determine the underlying cause of your symptoms.

A3.

The symptoms you described may be indicative of a condition called spinal stenosis, which occurs when the spaces within the spinal canal narrow, putting pressure on the nerves that travel through it. The narrowing of the spinal canal can be caused by several factors, including age-related degeneration of the spine, herniated discs, or spinal injuries.

The symptoms of spinal stenosis typically include pain, numbness, or weakness in the lower back, buttocks, and legs. The pain may worsen when standing or walking for extended periods and may improve when leaning forward, sitting, or lying down.

Other possible causes of these symptoms include herniated discs, sciatica, or peripheral neuropathy. It is important to consult a healthcare professional for a proper diagnosis and treatment plan. They may perform tests such as imaging studies, nerve conduction studies, or physical exams to determine the underlying cause of your symptoms. Treatment options may include pain medication, physical therapy, or surgery in severe cases.

A4.

The symptoms you described may be indicative of several different conditions that can affect the knee joint, and a healthcare professional would need to perform a thorough evaluation to make an accurate diagnosis. However, some possible conditions that could cause these symptoms include:

Osteoarthritis: This is a degenerative joint disease that occurs when the cartilage that cushions the ends of bones wears down over time. Osteoarthritis can cause pain, stiffness, and swelling in the knee joint, as well as cracking or popping sounds when bending the knee.

Rheumatoid arthritis: This is a chronic autoimmune disease that causes inflammation in the joints. Rheumatoid arthritis can affect any joint in the body, including the knee, and can cause pain, stiffness, and swelling.

Meniscal tears: The meniscus is a piece of cartilage in the knee joint that cushions and stabilizes the joint. Meniscal tears can occur from sudden twisting or bending of the knee and can cause swelling, pain, and stiffness in the knee joint.

Patellofemoral pain syndrome: This is a condition that occurs when the kneecap does not track properly over the femur, causing pain and swelling in the knee joint. Patellofemoral pain syndrome can cause a cracking or popping sensation when bending the knee.

It is important to consult a healthcare professional for a proper diagnosis and treatment plan. They may perform tests such as imaging studies, joint aspiration, or physical exams to determine the underlying cause of your symptoms. Treatment options may include pain medication, physical therapy, or surgery in severe cases.

A5.

The symptoms you described may be indicative of several different conditions that can affect the hip joint, and a healthcare professional would need to perform a thorough evaluation to make an accurate diagnosis. However, some possible conditions that could cause these symptoms include:

Osteoarthritis: This is a degenerative joint disease that occurs when the cartilage that cushions the ends of bones wears down over time. Osteoarthritis can cause pain, stiffness, and swelling in the hip joint, as well as cracking or popping sounds when moving the hip.

Rheumatoid arthritis: This is a chronic autoimmune disease that causes inflammation in the joints. Rheumatoid arthritis can affect any joint in the body, including the hip, and can cause pain, stiffness, and swelling.

Bursitis: This is a condition that occurs when the bursae, small fluid-filled sacs that cushion the joints, become inflamed. Bursitis can cause pain and stiffness in the hip joint, as well as cracking or popping sounds when moving the hip.

Labral tear: The labrum is a ring of cartilage that surrounds the hip joint, providing stability and cushioning. A labral tear can occur from sudden twisting or bending of the hip and can cause pain, stiffness, and popping sensations in the hip joint.

It is important to consult a healthcare professional for a proper diagnosis and treatment plan. They may perform tests such as imaging studies, joint aspiration, or physical exams to determine the underlying cause of your symptoms. Treatment options may include pain medication, physical therapy, or surgery in severe cases.

**Day 5**

A1.

These symptoms could be indicative of a few different conditions, but one possibility is carpal tunnel syndrome. Carpal tunnel syndrome is a condition in which the median nerve, which runs from the forearm into the hand, becomes compressed as it passes through the carpal tunnel in the wrist. This compression can cause tingling, numbness, and pain in the hand and fingers, especially at night.

Difficulty picking up small objects could also be a symptom of carpal tunnel syndrome, as it can affect fine motor skills.

It's important to note that there are other conditions that could cause similar symptoms, such as nerve damage or arthritis, so it's always best to consult with a healthcare provider for an accurate diagnosis and treatment plan.

A2.

The combination of numbness in the hands, difficulty with fine motor movements, and an unsteady walk could be indicative of a few different conditions, but one possibility is multiple sclerosis (MS).

MS is a chronic autoimmune disease that affects the central nervous system. It can cause a variety of symptoms, including numbness or tingling in the hands and other parts of the body, difficulty with coordination and balance, and problems with fine motor skills.

However, it's important to note that there are other conditions that could cause similar symptoms, such as peripheral neuropathy, Parkinson's disease, or spinal cord injury, so it's always best to consult with a healthcare provider for an accurate diagnosis and treatment plan.

A3.

The symptoms you describe could be indicative of spinal stenosis. Spinal stenosis is a condition in which the spaces within your spine narrow and put pressure on your spinal cord and nerves. This pressure can cause pain, numbness, or weakness in the lower back, buttocks, and legs, especially when standing or walking for extended periods. Leaning slightly forward can relieve the pressure and reduce the pain.

It's important to note that there are other conditions that could cause similar symptoms, such as herniated disc, sciatica, or osteoarthritis, so it's always best to consult with a healthcare provider for an accurate diagnosis and treatment plan.

A4.

The symptoms you describe could be indicative of a few different conditions, but one possibility is osteoarthritis. Osteoarthritis is a common form of arthritis that affects the joints, including the knee joint. It can cause pain, swelling, and stiffness in the affected joint, and cracking or popping sounds when you move the joint.

However, there are other conditions that could cause similar symptoms, such as a knee injury or another type of arthritis, so it's always best to consult with a healthcare provider for an accurate diagnosis and treatment plan. They may recommend imaging tests, such as X-rays or MRI, to help confirm the diagnosis.

A5.

The symptoms you describe could be indicative of a few different conditions, but one possibility is osteoarthritis of the hip joint. Osteoarthritis is a common form of arthritis that affects the joints, including the hip joint. It can cause pain, stiffness, and reduced range of motion in the hip joint, and cracking or popping sounds when you move the joint.

Other possible causes of hip pain and stiffness include hip bursitis, tendinitis, or a hip labral tear. It's important to see a healthcare provider for an accurate diagnosis and treatment plan. They may recommend imaging tests, such as X-rays or MRI, to help confirm the diagnosis. Treatment may include physical therapy, medication, or, in severe cases, surgery.

**Author, EY**

**Day 1**

A1.

Tingling and pain in the fingers, particularly at night, as well as difficulty picking up small objects, are common symptoms of carpal tunnel syndrome. This condition is caused by compression of the median nerve as it passes through the carpal tunnel, a narrow passageway in the wrist that is formed by the wrist bones and a ligament.

Carpal tunnel syndrome can be caused by a variety of factors, including repetitive hand motions, certain medical conditions such as diabetes and arthritis, hormonal changes, and injuries to the wrist. Treatment for carpal tunnel syndrome can include wrist splinting, nonsteroidal anti-inflammatory drugs (NSAIDs), corticosteroid injections, physical therapy, and in some cases, surgery. If you are experiencing these symptoms, it is important to see a doctor for an evaluation and to determine the best course of treatment.

A2.

The symptoms you described could be associated with several conditions, but one possible explanation could be a neurological disorder called peripheral neuropathy.

Peripheral neuropathy is a condition in which the nerves that carry information to and from the brain and spinal cord to the rest of the body are damaged or malfunctioning. The most common symptoms of peripheral neuropathy include numbness or tingling in the hands and feet, muscle weakness, difficulty with fine motor movements, and an unsteady gait.

However, it's important to note that these symptoms could also be indicative of other conditions, such as carpal tunnel syndrome, multiple sclerosis, or Parkinson's disease. It's best to consult a medical professional for a proper diagnosis and treatment plan. They may perform a physical exam and order additional tests, such as nerve conduction studies, electromyography, or imaging scans to help determine the underlying cause of your symptoms.

A3.

It is not possible to diagnose a specific disease or condition based solely on the information provided, but the symptoms you are describing could be associated with a herniated disc or spinal stenosis.

A herniated disc occurs when the soft center of a spinal disc pushes through a crack in the tougher exterior casing. This can cause pain and numbness in the lower back, buttocks, and legs, especially if the herniated disc is pressing on a nerve.

Spinal stenosis occurs when the spaces within the spine narrow, which can also put pressure on nerves and cause pain, numbness, or weakness in the lower back, buttocks, and legs. Symptoms often worsen with walking or standing and may improve with sitting or leaning forward.

However, other conditions could also cause these symptoms, so it's important to see a doctor for a proper diagnosis and treatment plan. Your doctor may recommend imaging tests, such as an X-ray, MRI, or CT scan, to get a better look at your spine and determine the underlying cause of your symptoms.

A4.

Swelling, pain, stiffness, and cracking sounds in the knee can be caused by a variety of conditions. It's difficult to determine the specific disease without a proper medical examination, but some common conditions that can cause these symptoms include:

Osteoarthritis: A degenerative joint disease that causes the cartilage in the knee joint to break down and wear away over time, leading to swelling, pain, stiffness, and cracking sounds.

Rheumatoid arthritis: An autoimmune disorder that causes chronic inflammation in the joints, leading to joint damage, swelling, pain, and stiffness.

Bursitis: Inflammation of the bursae, small fluid-filled sacs that cushion the knee joint, can cause swelling, pain, and stiffness.

Tendinitis: Inflammation of the tendons that attach to the knee joint can cause pain, swelling, and stiffness.

Meniscus tear: A tear in the meniscus, which is a piece of cartilage that acts as a shock absorber in the knee joint, can cause pain, swelling, and stiffness, as well as a sensation of locking or catching in the knee.

It's important to see a doctor for an accurate diagnosis and appropriate treatment.

A5.

There are several possible causes for hip pain, stiffness, and cracking when moving. Some of the common causes include:

Osteoarthritis: This is a degenerative joint disease that affects the cartilage of the joints, including the hip joint. It is a common cause of hip pain in older adults.

Rheumatoid arthritis: This is an autoimmune disease that affects the joints, causing pain, swelling, and stiffness. It can affect any joint, including the hip joint.

Bursitis: This is an inflammation of the bursa, a small fluid-filled sac that cushions the joints. Bursitis can cause hip pain, stiffness, and swelling.

Tendinitis: This is an inflammation of the tendons that attach muscles to bones. It can cause pain, stiffness, and weakness in the hip joint.

Hip labral tear: The labrum is a ring of cartilage that surrounds the hip joint. A tear in the labrum can cause hip pain, stiffness, and a clicking or popping sound when moving the hip.

It's important to see a doctor for an accurate diagnosis and appropriate treatment plan. They may order imaging tests, such as an X-ray or MRI, to evaluate the hip joint and determine the cause of the symptoms.

**Day 2**

A1.

The symptoms you described could be indicative of carpal tunnel syndrome. Carpal tunnel syndrome is a common condition that occurs when the median nerve, which runs through a narrow passage in your wrist (called the carpal tunnel), becomes compressed or pinched. This can result in tingling, numbness, or pain in the fingers, particularly the thumb, index, and middle fingers.

Other symptoms of carpal tunnel syndrome include weakness in the affected hand, difficulty gripping small objects, and a feeling of "pins and needles" in the affected hand and fingers. Symptoms are often worse at night and may wake you up from sleep.

Carpal tunnel syndrome can be caused by a variety of factors, including repetitive hand movements, wrist injuries, pregnancy, diabetes, and thyroid disorders. Treatment options may include wrist splinting, physical therapy, medication, or in severe cases, surgery. If you are experiencing these symptoms, it is important to consult with a healthcare professional for an accurate diagnosis and treatment plan.

A2.

Numbness in the hands, difficulty with fine movements, and unsteady walk, especially when going downstairs, can be symptoms of various medical conditions. It's not possible to make a diagnosis without a full medical evaluation. However, one possible condition that can cause these symptoms is peripheral neuropathy, a disorder that affects the nerves outside of the brain and spinal cord.

Peripheral neuropathy can be caused by a variety of factors, such as diabetes, alcoholism, infections, vitamin deficiencies, and certain medications. It can also occur as a result of nerve damage from an injury or trauma.

Other conditions that can cause similar symptoms include multiple sclerosis, Parkinson's disease, and carpal tunnel syndrome. It's important to see a healthcare professional for a thorough evaluation and diagnosis.

A3.

Based on the symptoms you've described, it's possible that you have a condition called spinal stenosis. This is a narrowing of the spaces within your spine, which can put pressure on the nerves that travel through it.

Symptoms of spinal stenosis often include pain or numbness in the lower back, buttocks, and legs. This pain may be worse when standing or walking, and may improve when bending forward. Other symptoms may include weakness or cramping in the legs, and difficulty with balance or coordination.

Spinal stenosis can be caused by a variety of factors, including age-related changes in the spine, herniated discs, or spinal injuries. Treatment options may include physical therapy, medications, and in some cases, surgery.

However, it's important to note that only a healthcare professional can provide a definitive diagnosis and treatment plan based on a physical examination and diagnostic tests. If you're experiencing these symptoms, you should see a doctor for evaluation and treatment.

A4.

There are several possible conditions that could cause these symptoms, including:

Osteoarthritis: This is a degenerative joint disease that can cause stiffness, swelling, and pain in the joints, particularly in weight-bearing joints such as the knee.

Rheumatoid arthritis: This is an autoimmune disease that causes inflammation in the joints, which can lead to pain, stiffness, and swelling.

Meniscus tear: The meniscus is a piece of cartilage in the knee that acts as a shock absorber between the thighbone and shinbone. If it tears, it can cause pain, swelling, and stiffness.

Bursitis: This is an inflammation of the bursae, small fluid-filled sacs that cushion the joints. Bursitis can cause pain and swelling in the affected joint.

Gout: This is a form of arthritis caused by a buildup of uric acid crystals in the joints, which can cause sudden attacks of pain, swelling, and stiffness.

It's best to see a doctor for an accurate diagnosis and treatment. They may recommend imaging tests such as X-rays or an MRI to help identify the cause of your symptoms.

A5.

Hip pain and stiffness, along with a cracking sound, can be caused by several conditions. Some common conditions that may cause these symptoms include:

Arthritis: This is a condition where the joint becomes inflamed and can cause pain, stiffness, and cracking sounds when moving.

Bursitis: Bursitis is inflammation of the bursa, a small fluid-filled sac near the hip joint. It can cause pain, swelling, and stiffness.

Tendinitis: This is inflammation of the tendons, which connect muscles to bones. It can cause pain, swelling, and stiffness.

Hip labral tear: The labrum is a ring of cartilage that lines the hip joint. A tear in this cartilage can cause pain, stiffness, and a clicking or popping sound.

Hip impingement: This is a condition where there is abnormal contact between the hip bone and the socket, causing pain and stiffness.

Osteonecrosis: Osteonecrosis is a condition where the bone in the hip joint dies due to lack of blood supply, causing pain, stiffness, and joint damage.

It is important to see a doctor for an accurate diagnosis and proper treatment.

**Day 3**

A1.

The symptoms you describe could be indicative of a condition called carpal tunnel syndrome. Carpal tunnel syndrome is a condition that occurs when the median nerve, which runs from your forearm into your hand, becomes compressed at the wrist. This compression can cause tingling, pain, and weakness in the fingers and hand.

Carpal tunnel syndrome is often caused by repetitive motions or prolonged periods of wrist flexion, such as using a computer mouse or typing on a keyboard. Other risk factors for carpal tunnel syndrome include obesity, pregnancy, and certain medical conditions such as diabetes and rheumatoid arthritis.

If you are experiencing symptoms of carpal tunnel syndrome, it is important to see a healthcare provider for an evaluation and diagnosis. Treatment options for carpal tunnel syndrome may include rest and modification of activities, physical therapy, wrist splints, medications, or in some cases, surgery.

A2.

The symptoms you have described can be associated with several different conditions. One possibility is a peripheral neuropathy, which is damage to the nerves outside of the brain and spinal cord. Other potential causes include carpal tunnel syndrome, multiple sclerosis, Parkinson's disease, or a cerebellar disorder.

To get a proper diagnosis and treatment, it is important to see a healthcare professional such as a neurologist, who can conduct a thorough examination and order any necessary tests to determine the underlying cause of your symptoms.

A3.

It is difficult to determine the exact disease without a proper medical examination and diagnosis by a healthcare professional. However, your symptoms may be indicative of a condition called lumbar spinal stenosis.

Lumbar spinal stenosis is a condition where the spinal canal in the lower back narrows, which can put pressure on the nerves that run through it. This can cause symptoms such as lower back pain, numbness, and tingling in the buttocks, legs, and feet, and weakness in the legs.

The pain increasing when walking and improving when leaning forward is a common symptom of lumbar spinal stenosis. This is because leaning forward can open up the space in the spinal canal and relieve pressure on the nerves.

It is important to see a healthcare professional for a proper diagnosis and treatment plan if you are experiencing these symptoms. They may recommend imaging tests, such as an MRI or CT scan, to confirm the diagnosis and suggest appropriate treatment options such as physical therapy, medications or in severe cases, surgery.

A4.

It's difficult to say exactly what is causing your symptoms without conducting a physical examination and possibly ordering diagnostic tests. However, based on the information you've provided, it's possible that you may be experiencing symptoms of osteoarthritis or a knee injury.

Osteoarthritis is a common form of arthritis that can affect the knee joint. It occurs when the protective cartilage that cushions the ends of your bones wears down over time, leading to pain, stiffness, and swelling.

A knee injury, such as a sprain or tear in the ligaments or cartilage of the knee joint, can also cause similar symptoms. Injuries can occur from overuse, sudden impact, or a twisting motion.

It's important to see a healthcare professional, such as a doctor or physical therapist, for a proper diagnosis and treatment plan. They may recommend rest, physical therapy, medication, or in severe cases, surgery.

A5.

As an AI language model, I cannot diagnose medical conditions, but your symptoms may suggest a hip joint problem. There are several conditions that can cause hip pain and stiffness, including:

Osteoarthritis: This is a degenerative joint disease that can cause pain, stiffness, and cracking sounds in the hip joint.

Rheumatoid arthritis: This is an autoimmune disease that can cause inflammation in the joints, leading to pain and stiffness.

Bursitis: This is the inflammation of the bursa, which is a small sac filled with fluid that cushions the joint. Bursitis can cause pain and stiffness in the hip joint.

Tendinitis: This is the inflammation of the tendons, which are the tissues that connect the muscles to the bones. Tendinitis can cause pain and stiffness in the hip joint.

Hip labral tear: This is a tear in the ring of cartilage that surrounds the hip joint. A hip labral tear can cause pain and stiffness in the hip joint.

It is important to see a doctor or a healthcare professional for a proper diagnosis and treatment plan. They may recommend imaging tests such as X-rays, MRI, or CT scans to get a better look at your hip joint. Based on the diagnosis, they may recommend medication, physical therapy, or in some cases, surgery to treat the condition.

**Day 4**

A1.

These symptoms could be indicative of a number of conditions, but one possibility is carpal tunnel syndrome. Carpal tunnel syndrome occurs when there is pressure on the median nerve, which runs through a narrow passageway in the wrist called the carpal tunnel. This pressure can cause tingling, numbness, and pain in the hand and fingers, particularly at night, and may make it difficult to grip or pick up small objects.

Other potential causes of these symptoms could include nerve compression or injury, arthritis, tendonitis, or a pinched nerve in the neck or shoulder. To receive a proper diagnosis and treatment plan, it's important to consult with a medical professional.

A2.

It is difficult to diagnose a disease based on a few symptoms alone, as many different conditions can cause numbness in the hands, difficulty with fine movements, and an unsteady gait. However, some possibilities include:

Carpal tunnel syndrome: This is a common condition caused by compression of the median nerve in the wrist. It can cause numbness, tingling, and weakness in the hands.

Multiple sclerosis: This is a chronic autoimmune disease that affects the central nervous system. It can cause a wide range of symptoms, including numbness or tingling in the hands or feet, difficulty with coordination and balance, and weakness.

Peripheral neuropathy: This is a condition in which the nerves outside the brain and spinal cord are damaged. It can cause numbness, tingling, and weakness in the hands and feet, as well as an unsteady gait.

Parkinson's disease: This is a chronic and progressive neurological disorder that affects movement. It can cause tremors, stiffness, and difficulty with balance and coordination.

It is important to see a doctor for a proper diagnosis and treatment plan. Your doctor may order tests such as nerve conduction studies or imaging tests to help diagnose the underlying cause of your symptoms.

A3.

The symptoms you described, including pain in the lower back, numbness and pain in the buttocks and calves, and relief when leaning forward, are commonly associated with a condition called spinal stenosis.

Spinal stenosis is a narrowing of the spinal canal, which puts pressure on the nerves that run through it. The condition can occur in different parts of the spine, but when it occurs in the lower back, it is called lumbar spinal stenosis. It can be caused by a variety of factors, including aging, arthritis, herniated discs, and injuries.

If you are experiencing these symptoms, it is important to see a healthcare professional for an evaluation and diagnosis. Your doctor may recommend treatments such as physical therapy, pain medication, and surgery in more severe cases.

A4.

I'm sorry to hear about your knee pain and swelling. There could be several possible causes for your symptoms, including:

Osteoarthritis: This is a degenerative joint disease that can cause pain, swelling, and stiffness in the knee joint. It is more common in older adults and can be worsened by overuse or injury.

Rheumatoid arthritis: This is an autoimmune disease that can cause joint pain, stiffness, and swelling. It can affect any joint in the body, including the knee.

Knee injury: An injury to the knee, such as a sprain or strain, can cause swelling and pain. It may also cause stiffness and cracking sounds when bending the knee.

Bursitis: This is an inflammation of the bursae, small fluid-filled sacs that cushion the knee joint. It can cause pain, swelling, and stiffness in the knee.

Gout: Gout is a type of arthritis that can cause sudden and severe pain, swelling, and redness in the knee joint.

It's important to see a doctor or healthcare provider to determine the cause of your knee pain and swelling. They may order imaging tests, such as X-rays or an MRI, to help diagnose the problem. Based on the underlying cause, treatment options may include medication, physical therapy, or surgery.

A5.

Hip pain and stiffness with cracking sounds can be caused by various conditions. It is essential to consult a doctor to get a proper diagnosis and treatment plan. However, some possible causes of your symptoms include:

Osteoarthritis: This is a degenerative joint disease that affects the cartilage lining the joints, causing pain, stiffness, and cracking sounds. It commonly affects older adults.

Bursitis: Bursitis is an inflammation of the bursae, small fluid-filled sacs that cushion the bones, muscles, and tendons near the joints. Bursitis can cause hip pain and stiffness.

Tendinitis: Tendinitis is an inflammation of the tendons that attach muscles to bones. It can cause pain and stiffness around the hip joint.

Hip labral tear: The labrum is a ring of cartilage that lines the socket of the hip joint. A tear in the labrum can cause hip pain, stiffness, and popping or clicking sounds.

Hip impingement: Hip impingement occurs when the bones of the hip joint rub against each other, causing pain and stiffness.

It is best to see a doctor or a healthcare professional to diagnose the cause of your symptoms and recommend appropriate treatment. They may recommend physical therapy, medication, or surgery depending on the underlying condition.

**Day 5**

A1.

These symptoms could be indicative of several possible conditions, but one common condition that matches your description is carpal tunnel syndrome.

Carpal tunnel syndrome occurs when the median nerve, which runs through the wrist and controls sensation and movement in the hand, becomes compressed or pinched at the wrist. This can cause symptoms such as pain, tingling, numbness, or weakness in the hand and fingers, especially at night or during activities that require repetitive hand movements.

Other conditions that could cause similar symptoms include arthritis, peripheral neuropathy, or cervical radiculopathy. However, a proper diagnosis can only be made by a medical professional after a thorough evaluation and possibly some diagnostic tests.

If you are experiencing these symptoms, it is important to consult with a doctor to determine the underlying cause and to receive appropriate treatment.

A2.

It is difficult to determine the specific disease or condition that is causing your symptoms without a proper medical evaluation. However, based on your symptoms, it is possible that you may be experiencing a neurological disorder.

Numbness in the hands and difficulty performing fine motor movements can be symptoms of conditions such as carpal tunnel syndrome, neuropathy, or multiple sclerosis. Unsteady walking, especially when going downstairs, can be a symptom of a condition affecting the cerebellum, which is responsible for balance and coordination.

It is important to seek medical attention from a qualified healthcare provider who can evaluate your symptoms and provide a proper diagnosis. They may conduct various tests, such as nerve conduction studies, blood tests, or imaging studies to determine the underlying cause of your symptoms and develop an appropriate treatment plan.

A3.

Based on your symptoms, it's possible that you may be experiencing a condition called spinal stenosis. Spinal stenosis is a narrowing of the spinal canal, which can put pressure on the spinal cord and nerves. It is most commonly caused by age-related changes in the spine, such as degenerative disc disease or osteoarthritis.

The symptoms of spinal stenosis can include pain in the lower back, as well as numbness, tingling, or weakness in the buttocks, legs, or feet. The pain may worsen with prolonged standing or walking, but improve when you lean forward or sit down.

However, it's important to note that there are other possible causes of lower back pain and leg symptoms, such as herniated discs or sciatica. It's best to see a healthcare professional for an accurate diagnosis and appropriate treatment.

A4.

Swelling, pain, stiffness, and cracking sounds in the knee when bending can be symptoms of several conditions. Without a proper medical examination, it is not possible to give an accurate diagnosis. However, here are some possible causes:

Knee osteoarthritis: This condition occurs when the protective cartilage in the knee joint wears down, causing pain, stiffness, and swelling.

Knee bursitis: This condition occurs when the bursae (small fluid-filled sacs that cushion the knee joint) become inflamed, causing pain and swelling.

Meniscus tear: The meniscus is a piece of cartilage that acts as a shock absorber between the bones in the knee joint. When it tears, it can cause pain, swelling, and stiffness in the knee, and can also produce a clicking or popping sound when you move your knee.

Knee ligament injury: A knee ligament injury, such as an anterior cruciate ligament (ACL) or medial collateral ligament (MCL) tear, can cause pain, swelling, and instability in the knee joint.

It is important to see a doctor or a healthcare professional for an accurate diagnosis and proper treatment of your knee condition.

A5.

Hip pain and stiffness with cracking sounds can be caused by several conditions, and it's essential to consult a medical professional to determine the cause and receive proper treatment. Some possible causes of hip pain and stiffness include:

Osteoarthritis: This is a common condition that affects the joints, causing them to wear down over time. It can cause pain and stiffness, especially after activity or extended periods of sitting or standing.

Bursitis: This is an inflammation of the bursae, which are small fluid-filled sacs that cushion the joints. Bursitis can cause pain and stiffness, especially when moving the joint.

Tendinitis: This is an inflammation of the tendons, which attach muscles to bones. Tendinitis can cause pain and stiffness in the affected area.

Hip labral tear: The labrum is a ring of cartilage that lines the rim of the hip socket. A tear in the labrum can cause pain and stiffness, especially when moving the hip.

Femoroacetabular impingement (FAI): FAI occurs when the bones in the hip joint are abnormally shaped, causing them to rub against each other. This can cause pain, stiffness, and cracking sounds.

It's important to see a doctor for a proper diagnosis and treatment plan. Depending on the cause, treatment options may include rest, physical therapy, medication, or in some cases, surgery.

**Author, AY**

**Day 1**

A1.

The symptoms you describe, especially tingling and pain in the fingers that are worse at night and difficulty picking up small objects, could be due to carpal tunnel syndrome.

Carpal tunnel syndrome is a common condition that occurs when the median nerve, which runs through a narrow passageway in the wrist called the carpal tunnel, becomes compressed. This compression can be caused by repetitive hand motions, injury to the wrist, or certain medical conditions like diabetes, hypothyroidism, or rheumatoid arthritis.

Other symptoms of carpal tunnel syndrome can include numbness, weakness, or a burning sensation in the fingers or hand, and pain that radiates up the arm. If you are experiencing these symptoms, it is important to see a healthcare provider for an accurate diagnosis and appropriate treatment. Treatment options for carpal tunnel syndrome may include rest, ice, splinting, physical therapy, medications, or in some cases, surgery.

A2.

The symptoms you describe, such as numbness in the hands, difficulty with fine movements, and an unsteady walk, could be due to a neurological disorder such as multiple sclerosis (MS).

MS is a chronic autoimmune disease that affects the central nervous system, including the brain, spinal cord, and optic nerves. The symptoms of MS can vary widely and depend on the specific area of the nervous system that is affected. Common symptoms include numbness or tingling in the extremities, muscle weakness, vision problems, and difficulty with coordination and balance.

Other conditions that may cause similar symptoms include peripheral neuropathy, which is damage to the peripheral nerves that can cause numbness, tingling, or weakness in the hands or feet, and Parkinson's disease, which can cause tremors, stiffness, and difficulty with movement.

If you are experiencing these symptoms, it is important to see a healthcare provider for an accurate diagnosis and appropriate treatment. They may perform a physical exam and order imaging tests, blood tests, or nerve conduction studies to help determine the cause of your symptoms.

A3.

The symptoms you describe, such as lower back pain, numbness and pain in the buttocks and calves that worsens with walking but improves when leaning slightly forward, may be indicative of a condition called spinal stenosis.

Spinal stenosis is a narrowing of the spaces within the spine that can put pressure on the spinal cord or nerves. This condition is most commonly seen in older adults, and can be caused by age-related changes in the spine, such as arthritis or degenerative disc disease.

Other symptoms of spinal stenosis can include weakness, tingling or numbness in the legs, and difficulty with balance or coordination. In some cases, spinal stenosis can lead to more serious complications, such as bowel or bladder dysfunction.

If you are experiencing these symptoms, it is important to see a healthcare provider for an accurate diagnosis and appropriate treatment. They may perform a physical exam, imaging tests such as an MRI or CT scan, or nerve conduction studies to help determine the cause of your symptoms. Treatment options for spinal stenosis may include physical therapy, pain management, and in some cases, surgery.

A4.

The symptoms you describe, such as swelling, pain when walking, stiffness, and cracking sounds when bending the knee, may be indicative of a condition called osteoarthritis.

Osteoarthritis is a degenerative joint disease that occurs when the protective cartilage that cushions the ends of bones within a joint wears down over time. As the cartilage breaks down, the bones can rub against each other, causing pain, stiffness, and swelling.

Other symptoms of osteoarthritis can include limited range of motion, muscle weakness, and the development of bone spurs around the affected joint.

If you are experiencing these symptoms, it is important to see a healthcare provider for an accurate diagnosis and appropriate treatment. They may perform a physical exam, imaging tests such as X-rays or an MRI, or joint aspiration to help determine the cause of your symptoms. Treatment options for osteoarthritis may include physical therapy, pain management, and in some cases, joint replacement surgery.

A5.

The symptoms you describe, such as pain in the hip when walking, stiffness, and cracking sounds when moving the hip, may be indicative of a condition called osteoarthritis.

Osteoarthritis is a degenerative joint disease that occurs when the protective cartilage that cushions the ends of bones within a joint wears down over time. As the cartilage breaks down, the bones can rub against each other, causing pain, stiffness, and swelling.

Other symptoms of osteoarthritis can include limited range of motion, muscle weakness, and the development of bone spurs around the affected joint.

If you are experiencing these symptoms, it is important to see a healthcare provider for an accurate diagnosis and appropriate treatment. They may perform a physical exam, imaging tests such as X-rays or an MRI, or joint aspiration to help determine the cause of your symptoms. Treatment options for osteoarthritis may include physical therapy, pain management, and in some cases, joint replacement surgery.

**Day 2**

A1.

The symptoms you are describing, such as tingling and pain in the fingers, especially at night, and difficulty picking up small objects, are commonly associated with a condition called carpal tunnel syndrome.

Carpal tunnel syndrome is a condition that affects the median nerve, which runs from your forearm to your hand through a small space in your wrist called the carpal tunnel. When this space becomes narrowed, it can put pressure on the median nerve and cause the symptoms you are experiencing.

There can be various causes of carpal tunnel syndrome, such as repetitive hand and wrist movements, arthritis, and hormonal changes during pregnancy or menopause. It is also associated with underlying health conditions like diabetes, thyroid disorders, and obesity.

It is important to see a healthcare professional for a proper diagnosis and treatment plan. They may perform a physical exam, imaging tests, and nerve conduction studies to confirm the diagnosis and recommend treatment options such as wrist splinting, physical therapy, and in severe cases, surgery.

A2.

The symptoms you are describing, including numbness in the hands, difficulty with fine motor movements, and an unsteady walk, may be associated with a neurological condition called peripheral neuropathy.

Peripheral neuropathy refers to damage or dysfunction of the peripheral nerves, which are the nerves outside the brain and spinal cord. This can result in a range of symptoms, such as numbness, tingling, and weakness in the hands and feet, and difficulty with balance and coordination.

There can be many causes of peripheral neuropathy, such as diabetes, alcoholism, vitamin deficiencies, infections, and exposure to toxins. In some cases, the cause of peripheral neuropathy may not be clear.

It is important to see a healthcare professional for a proper diagnosis and treatment plan. They may perform a physical exam, blood tests, nerve conduction studies, and other tests to help determine the underlying cause of your symptoms. Treatment may depend on the underlying cause and may include medications, physical therapy, and lifestyle changes.

A3.

The symptoms you are describing, including lower back pain, numbness and pain in the buttocks and calves, and pain that increases with walking but improves when leaning slightly forward, may be associated with a condition called spinal stenosis.

Spinal stenosis is a condition that occurs when the spaces within the spinal canal become narrowed, which can put pressure on the nerves that run through the spine. This can cause a range of symptoms, including lower back pain, numbness or tingling in the buttocks and legs, and weakness in the legs.

There are different types of spinal stenosis, but the most common type is called lumbar spinal stenosis, which affects the lower back. Lumbar spinal stenosis is often caused by age-related changes in the spine, such as arthritis, degenerative disc disease, and thickening of ligaments.

It is important to see a healthcare professional for a proper diagnosis and treatment plan. They may perform a physical exam, imaging tests, and nerve conduction studies to confirm the diagnosis and recommend treatment options, such as physical therapy, pain medication, and in severe cases, surgery. Making lifestyle changes, such as maintaining a healthy weight and avoiding activities that aggravate your symptoms, may also be recommended.

A4.

The symptoms you are describing, including swelling and pain in the knee, stiffness, and cracking sounds when bending the knee, are often associated with a condition called osteoarthritis.

Osteoarthritis is a degenerative joint disease that occurs when the protective cartilage that cushions the ends of bones wears down over time. This can cause a range of symptoms, including pain, swelling, stiffness, and a reduced range of motion in the affected joint. Osteoarthritis is commonly found in weight-bearing joints such as the knee.

There can be many causes of osteoarthritis, such as age, genetics, previous injury or overuse of the joint, and obesity.

It is important to see a healthcare professional for a proper diagnosis and treatment plan. They may perform a physical exam and imaging tests such as X-rays or MRI to confirm the diagnosis and recommend treatment options such as pain medication, physical therapy, and in some cases, surgery. Making lifestyle changes such as losing weight, avoiding activities that aggravate your symptoms, and incorporating low-impact exercises such as swimming and cycling can also help manage the symptoms of osteoarthritis.

A5.

The symptoms you are describing, including hip pain when walking, stiffness, and cracking sounds when moving the hip, can be associated with a few different conditions, but one of the most common is osteoarthritis of the hip.

Osteoarthritis is a degenerative joint disease that occurs when the cartilage that cushions the ends of bones wears down over time, causing pain, swelling, and stiffness in the affected joint. Osteoarthritis is common in weight-bearing joints such as the hip, and it can develop due to factors such as age, genetics, previous injury or overuse of the joint, and obesity.

Other potential causes of hip pain and stiffness include hip bursitis, tendinitis, or a hip labral tear. Hip bursitis and tendinitis are caused by inflammation of the bursae or tendons in the hip, respectively, which can cause pain and stiffness. A hip labral tear is a tear in the ring of cartilage that surrounds the hip joint and can cause pain, stiffness, and limited range of motion in the hip.

It is important to see a healthcare professional for a proper diagnosis and treatment plan. They may perform a physical exam, imaging tests such as X-rays or MRI, and other tests to confirm the diagnosis and recommend treatment options such as pain medication, physical therapy, and in some cases, surgery. Making lifestyle changes such as losing weight, avoiding activities that aggravate your symptoms, and incorporating low-impact exercises such as swimming and cycling can also help manage the symptoms of hip pain and stiffness.

**Day 3**

A1.

The symptoms you are experiencing may be indicative of a condition known as carpal tunnel syndrome. This occurs when the median nerve, which runs from the forearm into the hand, becomes compressed at the wrist. This compression can lead to pain, numbness, and tingling in the fingers, particularly at night.

Carpal tunnel syndrome is often associated with repetitive motions of the hands and wrists, such as typing or using a mouse. It may also be caused by other factors such as injury, arthritis, or thyroid dysfunction.

It's important to see a healthcare professional for a proper diagnosis and treatment plan. Treatment options may include rest and immobilization of the affected wrist, physical therapy, medications, or in severe cases, surgery.

A2.

The symptoms you are experiencing may be indicative of a neurological condition known as peripheral neuropathy. Peripheral neuropathy refers to damage or dysfunction of the peripheral nerves, which are the nerves outside of the brain and spinal cord.

Numbness in the hands is a common symptom of peripheral neuropathy, and difficulty with fine movements and an unsteady gait can also be associated with this condition. Peripheral neuropathy can have a variety of causes, including diabetes, autoimmune disorders, infections, exposure to toxins, and certain medications.

It's important to see a healthcare professional for a proper diagnosis and treatment plan. Treatment options may include addressing the underlying cause of the neuropathy, medications to manage symptoms such as pain or tingling, and physical therapy to improve muscle strength and coordination.

The symptoms you are experiencing may be indicative of a neurological condition known as peripheral neuropathy. Peripheral neuropathy refers to damage or dysfunction of the peripheral nerves, which are the nerves outside of the brain and spinal cord.

Numbness in the hands is a common symptom of peripheral neuropathy, and difficulty with fine movements and an unsteady gait can also be associated with this condition. Peripheral neuropathy can have a variety of causes, including diabetes, autoimmune disorders, infections, exposure to toxins, and certain medications.

It's important to see a healthcare professional for a proper diagnosis and treatment plan. Treatment options may include addressing the underlying cause of the neuropathy, medications to manage symptoms such as pain or tingling, and physical therapy to improve muscle strength and coordination.

A3.

The symptoms you are experiencing may be indicative of a condition known as spinal stenosis. Spinal stenosis is a narrowing of the spinal canal, which can put pressure on the nerves that travel through the spine. This pressure can cause pain, numbness, or weakness in the lower back, buttocks, and legs.

The pain you describe that increases with walking and improves when leaning forward is a common symptom of spinal stenosis, as the forward-leaning position can help to relieve the pressure on the nerves.

Spinal stenosis can have a variety of causes, including degenerative changes in the spine due to aging, herniated discs, or spinal injuries. It's important to see a healthcare professional for a proper diagnosis and treatment plan. Treatment options may include physical therapy, pain management, and in severe cases, surgery to relieve the pressure on the nerves.

A4.

The symptoms you are experiencing may be indicative of a condition known as osteoarthritis of the knee. Osteoarthritis is a degenerative joint disease that occurs when the cartilage that cushions the joints breaks down, leading to pain, stiffness, and swelling.

The cracking sound that you hear when bending your knee may be due to the roughening of the surfaces of the bones within the knee joint.

Osteoarthritis can have a variety of causes, including age, genetics, and prior knee injuries. It's important to see a healthcare professional for a proper diagnosis and treatment plan. Treatment options may include pain management, physical therapy, and lifestyle modifications such as weight loss and low-impact exercise. In severe cases, surgery may be necessary to replace the damaged knee joint.

A5.

The symptoms you are experiencing may be indicative of a condition known as osteoarthritis of the hip. Osteoarthritis is a degenerative joint disease that occurs when the cartilage that cushions the joints breaks down, leading to pain, stiffness, and swelling.

The cracking sound that you hear when moving your hip may be due to the roughening of the surfaces of the bones within the hip joint.

Osteoarthritis can have a variety of causes, including age, genetics, and prior hip injuries. It's important to see a healthcare professional for a proper diagnosis and treatment plan. Treatment options may include pain management, physical therapy, and lifestyle modifications such as weight loss and low-impact exercise. In severe cases, surgery may be necessary to replace the damaged hip joint.

**Day 4**

A1.

The symptoms you described could be related to a condition called carpal tunnel syndrome. Carpal tunnel syndrome occurs when the median nerve, which runs from your forearm into your hand, becomes compressed or squeezed at the wrist. This can cause pain, numbness, tingling, and weakness in the affected hand and fingers, especially at night. The difficulty in picking up small objects may be due to weakness or numbness in the fingers.

Other conditions such as arthritis, tendinitis, or nerve damage can also cause similar symptoms. It is important to consult a healthcare professional to get an accurate diagnosis and appropriate treatment plan.

A2.

The symptoms you have described can be caused by several conditions. However, based on the combination of symptoms you mentioned, it is possible that you may have a neurological condition such as peripheral neuropathy, which can affect the nerves that carry information from your brain and spinal cord to your limbs and organs.

Peripheral neuropathy can cause numbness, tingling, and weakness in the hands and feet. It can also affect your ability to perform fine movements such as handling small objects or buttoning a shirt. Additionally, some people with peripheral neuropathy may experience an unsteady gait or difficulty walking, especially when going downstairs.

However, other conditions such as carpal tunnel syndrome, multiple sclerosis, or Parkinson's disease can also cause similar symptoms. Therefore, it is essential to seek medical advice and undergo proper medical evaluation to determine the underlying cause of your symptoms and receive appropriate treatment.

A3.

The symptoms you have described suggest that you may have a condition called spinal stenosis. Spinal stenosis is a narrowing of the spinal canal, which can put pressure on the nerves that run through it. This pressure can cause pain, numbness, and tingling in the lower back, buttocks, and legs, especially during activities like walking or standing. The pain may improve when you lean slightly forward because this position opens up the spinal canal and reduces the pressure on the affected nerves.

Spinal stenosis can be caused by a variety of factors, such as aging, arthritis, herniated discs, or spinal injuries. It is essential to seek medical advice and undergo proper medical evaluation to determine the underlying cause of your symptoms and receive appropriate treatment. Treatment options may include physical therapy, medications, or surgery, depending on the severity and underlying cause of the spinal stenosis.

A4.

The symptoms you described could be caused by a condition called osteoarthritis of the knee. Osteoarthritis is a common form of arthritis that occurs when the protective cartilage on the ends of bones wears down over time. This can cause pain, swelling, stiffness, and cracking sounds in the affected joint, especially when walking or moving the joint.

Other possible causes of knee pain and swelling include injuries such as a torn ligament or meniscus, bursitis, or tendonitis. A healthcare professional can help you determine the underlying cause of your symptoms through a physical examination and imaging tests if necessary. Treatment options for osteoarthritis of the knee may include pain relievers, physical therapy, and in some cases, surgery.

A5.

The symptoms you described could be caused by a condition called osteoarthritis of the hip. Osteoarthritis is a common form of arthritis that occurs when the protective cartilage on the ends of bones wears down over time. This can cause pain, stiffness, and cracking sounds in the affected joint, especially when walking or moving the joint.

Other possible causes of hip pain and stiffness include injuries such as a hip labral tear, bursitis, or tendonitis. A healthcare professional can help you determine the underlying cause of your symptoms through a physical examination and imaging tests if necessary.

Treatment options for osteoarthritis of the hip may include pain relievers, physical therapy, and in some cases, surgery. It is important to seek medical advice and undergo proper medical evaluation to determine the underlying cause of your symptoms and receive appropriate treatment.

**Day 5**

A1.

These symptoms could be indicative of a condition called Carpal Tunnel Syndrome. Carpal Tunnel Syndrome occurs when the median nerve, which runs from your forearm to your hand through a narrow passageway in your wrist called the carpal tunnel, becomes compressed or squeezed. This can result in pain, tingling, numbness, and weakness in the fingers and hand.

Carpal Tunnel Syndrome is often associated with repetitive motions of the hand and wrist, such as typing on a keyboard or using tools, but can also be caused by other factors such as obesity, pregnancy, or arthritis.

If you are experiencing these symptoms, it is important to consult a healthcare professional for proper diagnosis and treatment. Treatment options may include rest and activity modification, physical therapy, medication, or in severe cases, surgery.

A2.

The symptoms you are describing may be indicative of a neurological condition called peripheral neuropathy. Peripheral neuropathy is a condition that affects the peripheral nerves, which are the nerves that transmit signals between the central nervous system (brain and spinal cord) and the rest of the body.

Numbness and tingling in the hands and feet are common symptoms of peripheral neuropathy. The difficulty in performing fine motor movements and unsteady gait can also be symptoms of this condition. Peripheral neuropathy can have many causes, including diabetes, autoimmune disorders, infections, exposure to toxins, and certain medications.

It is important to consult a healthcare professional for proper diagnosis and treatment if you are experiencing these symptoms. Treatment options may include medication, physical therapy, or addressing the underlying cause of the condition. Early treatment can help prevent further nerve damage and improve outcomes.

A3.

The symptoms you are describing may be indicative of a condition called lumbar spinal stenosis. Lumbar spinal stenosis is a narrowing of the spinal canal in the lower back, which can compress and irritate the nerves that run through it.

The pain in the lower back, buttocks, and calves, as well as numbness, tingling, and weakness in the legs, are common symptoms of lumbar spinal stenosis. The pain may increase with prolonged standing or walking and may improve with bending forward or sitting down.

Lumbar spinal stenosis is most commonly caused by age-related degenerative changes in the spine, such as arthritis or herniated discs. Other causes may include tumors or injuries to the spine.

If you are experiencing these symptoms, it is important to consult a healthcare professional for proper diagnosis and treatment. Treatment options may include medication, physical therapy, or in severe cases, surgery. Your doctor can recommend the best course of treatment based on the severity and underlying cause of your condition.

A4.

The symptoms you are describing may be indicative of a condition called osteoarthritis of the knee. Osteoarthritis is a degenerative joint disease that can affect any joint in the body, but it commonly affects weight-bearing joints such as the knee.

Swelling, pain, stiffness, and cracking sounds when bending the knee are common symptoms of osteoarthritis of the knee. The pain may be worse with activity and may improve with rest. Osteoarthritis is more common in older adults, but it can also develop as a result of injury or overuse.

If you are experiencing these symptoms, it is important to consult a healthcare professional for proper diagnosis and treatment. Treatment options may include medication, physical therapy, or assistive devices such as braces or canes to support the knee. In severe cases, surgery may be recommended.

Early diagnosis and treatment of osteoarthritis can help slow the progression of the disease and relieve symptoms, so it is important to seek medical attention if you suspect you have osteoarthritis of the knee.

A5.

The symptoms you are describing may be indicative of a condition called osteoarthritis of the hip. Osteoarthritis is a degenerative joint disease that can affect any joint in the body, but it commonly affects weight-bearing joints such as the hip.

Pain, stiffness, and cracking sounds when moving the hip are common symptoms of osteoarthritis of the hip. The pain may be worse with activity and may improve with rest. Osteoarthritis is more common in older adults, but it can also develop as a result of injury or overuse.

If you are experiencing these symptoms, it is important to consult a healthcare professional for proper diagnosis and treatment. Treatment options may include medication, physical therapy, or assistive devices such as canes or crutches to support the hip. In severe cases, surgery may be recommended.

Early diagnosis and treatment of osteoarthritis can help slow the progression of the disease and relieve symptoms, so it is important to seek medical attention if you suspect you have osteoarthritis of the hip.
