# Supplementary material for: The Potential of ChatGPT as a Self-Diagnostic Tool in Common Orthopedic Diseases: Exploratory Study
Source: J Med Internet Res. 2023 Sep 15;25:e47621. doi: 10.2196/47621 (PMC10541638; doi:10.2196/47621)
Supplement: Multimedia Appendix 3 [file jmir_v25i1e47621_app3.doc]

**Author, KF**

**Day 1**

**Q1a.**

Based on your symptoms, it is possible that you may have carpal tunnel syndrome, which is a condition that affects the hand and wrist. Carpal tunnel syndrome occurs when the median nerve, which runs from the forearm to the hand through a narrow passageway called the carpal tunnel, is compressed or squeezed at the wrist.

Here are five potential differential diagnoses:

1. Rheumatoid arthritis: This is an autoimmune disease that causes inflammation in the joints, including those in the hands and fingers. It can cause pain, stiffness, and swelling, which can lead to difficulty with fine motor skills and gripping objects.

2. Peripheral neuropathy: This is a condition that occurs when there is damage to the peripheral nerves, which are the nerves that connect the brain and spinal cord to the rest of the body. It can cause tingling, numbness, and pain in the hands and feet.

3. Dupuytren's contracture: This is a condition that causes the tissue in the palm of the hand to thicken and tighten, which can make it difficult to straighten the fingers. It can cause pain and difficulty with fine motor skills.

4. Trigger finger: This is a condition that occurs when the tendons in the fingers become inflamed, causing pain and difficulty with movement. It can cause the finger to get stuck in a bent position.

5. Cubital tunnel syndrome: This is a condition that occurs when the ulnar nerve, which runs from the elbow to the hand, is compressed or squeezed at the elbow. It can cause pain, tingling, and weakness in the hand and fingers.

**Q1c.**

Based on your symptoms, it is possible that you may have carpal tunnel syndrome. This is a condition that affects the hand and wrist, and occurs when the median nerve, which runs from the forearm to the hand through a narrow passageway called the carpal tunnel, is compressed or squeezed at the wrist. This compression can cause tingling, numbness, and pain in the hand and fingers, especially at night. Difficulty picking up small objects can also be a symptom of carpal tunnel syndrome. However, it's important to note that a definitive diagnosis can only be made by a medical professional after a thorough examination and any necessary testing.

**Q2a.**

Based on your symptoms, it is possible that you may have a neurological disorder such as multiple sclerosis (MS). MS is a chronic autoimmune disease that affects the central nervous system, which includes the brain and spinal cord. The symptoms of MS can vary widely, but some common symptoms include numbness or tingling in the hands and feet, difficulty with fine movements, and problems with balance and coordination.

Here are five potential differential diagnoses:

1. Peripheral neuropathy: This is a condition that occurs when there is damage to the peripheral nerves, which are the nerves that connect the brain and spinal cord to the rest of the body. It can cause numbness, tingling, and weakness in the hands and feet.

2. Diabetic neuropathy: This is a type of peripheral neuropathy that occurs in people with diabetes. It can cause numbness, tingling, and pain in the hands and feet, as well as difficulty with fine movements.

3. Parkinson's disease: This is a progressive neurological disorder that affects movement. It can cause tremors, stiffness, and difficulty with balance and coordination.

4. Cervical spondylosis: This is a degenerative condition that affects the cervical spine (neck). It can cause numbness, tingling, and weakness in the arms and hands, as well as problems with balance and coordination.

5. Stroke: This is a medical emergency that occurs when the blood supply to the brain is interrupted. It can cause a wide range of symptoms, including numbness, weakness, and difficulty with movement and coordination.

**Q2b.**

Based on your symptoms, it is possible that you may have carpal tunnel syndrome. This is a condition that affects the hand and wrist, and occurs when the median nerve, which runs from the forearm to the hand through a narrow passageway called the carpal tunnel, is compressed or squeezed at the wrist. This compression can cause numbness, tingling, and weakness in the hand and fingers, especially when performing fine movements such as handling small objects. Difficulty with buttoning a shirt is a common complaint in individuals with carpal tunnel syndrome. However, it's important to note that a definitive diagnosis can only be made by a medical professional after a thorough examination and any necessary testing. Other conditions such as peripheral neuropathy or cervical spondylosis can also cause similar symptoms, so it's important to seek medical attention for an accurate diagnosis and appropriate treatment.

**Q3a.**

Based on your symptoms, it is possible that you may have a condition called lumbar spinal stenosis. This is a condition where the spinal canal in the lower back narrows and puts pressure on the nerves that run through the spine. This can cause pain, numbness, and weakness in the buttocks, legs, and feet, especially during walking or standing upright.

Here are five potential differential diagnoses:

1. Herniated disc: This is a condition where the soft tissue in between the vertebrae in the spine ruptures, causing pressure on the nerves and resulting in pain, numbness, and weakness.

2. Sciatica: This is a condition where the sciatic nerve, which runs from the lower back down the legs, is compressed or irritated, causing pain, numbness, and weakness in the buttocks and legs.

3. Spondylolisthesis: This is a condition where one vertebra in the spine slips forward over the vertebra below it, causing pressure on the nerves and resulting in pain, numbness, and weakness in the lower back and legs.

4. Osteoarthritis: This is a degenerative joint disease that can affect the lower back and hips, causing pain and stiffness that may be worse with activity.

5. Piriformis syndrome: This is a condition where the piriformis muscle, which runs from the lower back to the hip, becomes tight or inflamed, causing pressure on the sciatic nerve and resulting in pain, numbness, and weakness in the buttocks and legs.

**Q4a.**

Based on your symptoms, it is possible that you may have a knee joint condition called osteoarthritis. This is a degenerative joint disease that affects the cartilage, which is the cushioning material that covers the ends of the bones in the knee joint. Osteoarthritis can cause pain, stiffness, and swelling in the knee joint, and may also cause cracking or popping sounds when the knee is moved.

Here are five potential differential diagnoses:

1. Rheumatoid arthritis: This is an autoimmune disorder that can affect the joints, including the knee joint, causing pain, swelling, stiffness, and deformity.

2. Bursitis: This is a condition where the bursae, which are small fluid-filled sacs that cushion the knee joint, become inflamed, causing pain and swelling.

3. Meniscus tear: This is a tear in the cartilage that cushions the knee joint, which can cause pain, swelling, and stiffness in the knee, especially when walking or bending the knee.

4. Gout: This is a type of arthritis caused by a build-up of uric acid crystals in the joint, which can cause sudden episodes of severe pain, swelling, and stiffness in the knee joint.

5. Ligament injury: This is a tear or sprain in one of the ligaments that supports the knee joint, such as the anterior cruciate ligament (ACL) or the medial collateral ligament (MCL), which can cause pain, swelling, and instability in the knee joint.

**Q4c.**

Based on your symptoms, it is possible that you may have osteoarthritis of the knee joint. Osteoarthritis is a degenerative joint disease that affects the cartilage, which is the cushioning material that covers the ends of the bones in the joint. This can cause pain, swelling, stiffness, and a sensation of cracking or popping when the joint is moved.

However, it's important to note that a definitive diagnosis can only be made by a medical professional after a thorough examination and any necessary testing. Other conditions such as rheumatoid arthritis, bursitis, meniscus tears, ligament injuries, or gout can also cause similar symptoms, so it's important to seek medical attention for an accurate diagnosis and appropriate treatment.

**Q5a.**

Based on your symptoms, it is possible that you may have hip osteoarthritis. Osteoarthritis is a degenerative joint disease that affects the cartilage in the hip joint, causing pain, stiffness, and a sensation of cracking or popping when the joint is moved.

Here are five potential differential diagnoses:

1. Bursitis: This is a condition where the bursae, which are small fluid-filled sacs that cushion the hip joint, become inflamed, causing pain and tenderness in the hip.

2. Hip labral tear: The labrum is a ring of cartilage that helps to stabilize the hip joint. A tear in the labrum can cause pain in the hip and a sensation of catching or clicking.

3. Avascular necrosis: This is a condition where the blood supply to the hip joint is interrupted, causing damage to the bone and cartilage, and resulting in pain and stiffness in the hip.

4. Hip fracture: A hip fracture is a break in the bone of the hip joint, usually caused by trauma. This can cause severe pain in the hip, difficulty walking or standing, and swelling or bruising in the hip area.

5. Hip impingement: This is a condition where the bones in the hip joint rub against each other, causing pain and stiffness. Hip impingement can be caused by abnormalities in the shape of the bones or by damage to the soft tissue in the hip joint.

**Q5c.**

Based on your symptoms, it's possible that you may have hip osteoarthritis. Osteoarthritis is a degenerative joint disease that can affect the hip joint and cause pain, stiffness, and a sensation of cracking or popping when the joint is moved.

However, it's important to note that a definitive diagnosis can only be made by a medical professional after a thorough examination and any necessary testing. Other conditions such as bursitis, tendonitis, hip labral tear, avascular necrosis, or hip impingement can also cause similar symptoms, so it's important to seek medical attention for an accurate diagnosis and appropriate treatment.

**Day 2**

**Q1a.**

Based on your symptoms, it is possible that you have Carpal Tunnel Syndrome (CTS), which is a common condition that affects the hand and wrist. The symptoms of CTS usually include tingling, numbness, and pain in the fingers (especially at night), as well as difficulty picking up small objects. However, a proper diagnosis can only be made by a medical professional after a physical examination and diagnostic testing.

Here are five potential differential diagnoses to consider:

1. Cubital Tunnel Syndrome: This condition involves the compression of the ulnar nerve at the elbow, which can cause tingling, numbness, and pain in the fingers, particularly the ring and little finger.

2. Radial Tunnel Syndrome: This condition involves the compression of the radial nerve near the elbow, which can cause pain and weakness in the forearm, wrist, and hand.

3. Peripheral Neuropathy: This is a general term that refers to damage or dysfunction of the peripheral nerves, which can cause a variety of symptoms, including tingling, numbness, and pain in the hands and feet.

4. Rheumatoid Arthritis: This is an autoimmune disorder that causes inflammation in the joints, which can lead to pain, stiffness, and swelling in the hands and fingers.

5. Multiple Sclerosis: This is a chronic autoimmune disorder that affects the central nervous system, which can cause a variety of symptoms, including tingling, numbness, and weakness in the hands and fingers.

**Q1c.**

Based on your symptoms, it is possible that you have Carpal Tunnel Syndrome (CTS). CTS is a common condition that affects the hand and wrist. It is caused by compression of the median nerve as it passes through the carpal tunnel in the wrist. The symptoms of CTS usually include tingling, numbness, and pain in the fingers (especially at night), as well as difficulty picking up small objects. However, a proper diagnosis can only be made by a medical professional after a physical examination and diagnostic testing.

**Q2a.**

Based on your symptoms, it is possible that you have a neurological condition that affects your motor and sensory functions. One potential primary diagnosis could be Multiple Sclerosis (MS), a chronic autoimmune disease that affects the central nervous system. MS can cause a wide range of symptoms, including numbness or tingling in the hands and feet, difficulty with fine movements, and an unsteady gait. However, a proper diagnosis can only be made by a medical professional after a physical examination and diagnostic testing.

Here are five potential differential diagnoses to consider:

1. Peripheral neuropathy: This is a general term for nerve damage that can cause numbness, tingling, and weakness in the hands and feet. It can be caused by a variety of factors, including diabetes, alcoholism, and certain medications.

2. Amyotrophic Lateral Sclerosis (ALS): ALS is a progressive neurodegenerative disease that affects the motor neurons in the brain and spinal cord. It can cause muscle weakness, difficulty with fine movements, and an unsteady gait.

3. Parkinson's disease: Parkinson's is a neurodegenerative disorder that affects movement. It can cause tremors, stiffness, and difficulty with fine movements, as well as an unsteady gait.

4. Cervical spondylosis: This is a degenerative condition that affects the bones and discs in the neck. It can cause compression of the nerves that control the hands and arms, leading to numbness, tingling, and weakness.

5. Guillain-Barré syndrome: This is a rare disorder in which the body's immune system attacks the nerves. It can cause muscle weakness, numbness, and tingling in the hands and feet, as well as difficulty with fine movements.

**Q2b.**

Based on your symptoms, it is possible that you have Carpal Tunnel Syndrome (CTS). CTS is a common condition that affects the hand and wrist. It is caused by compression of the median nerve as it passes through the carpal tunnel in the wrist. The symptoms of CTS usually include numbness, tingling, and weakness in the hands, especially in the thumb, index, and middle fingers, as well as difficulty with fine movements, such as buttoning a shirt. However, a proper diagnosis can only be made by a medical professional after a physical examination and diagnostic testing. Other potential causes of these symptoms include peripheral neuropathy, cervical radiculopathy, and certain vitamin deficiencies.

**Q3a.**

Based on your symptoms, it is possible that you have a condition called Lumbar Spinal Stenosis (LSS). LSS is a narrowing of the spinal canal in the lower back, which can cause compression of the nerves that run through the spinal cord. The symptoms of LSS usually include lower back pain, numbness or tingling in the buttocks and legs, and difficulty with walking or standing for long periods of time. Leaning forward can help alleviate the symptoms by increasing the space in the spinal canal.

Here are five potential differential diagnoses to consider:

1. Herniated disc: A herniated disc occurs when the soft inner portion of a spinal disc protrudes through the tougher outer portion, which can cause pressure on the spinal nerves and result in lower back pain and numbness or tingling in the legs.

2. Sciatica: Sciatica is a condition that occurs when the sciatic nerve, which runs from the lower back down through the legs, is compressed or irritated. This can cause lower back pain and numbness, tingling, or weakness in the legs.

3. Piriformis syndrome: Piriformis syndrome occurs when the piriformis muscle in the buttocks becomes tight or irritated, which can put pressure on the sciatic nerve and cause lower back pain and numbness or tingling in the legs.

4. Degenerative disc disease: Degenerative disc disease is a condition in which the spinal discs between the vertebrae break down over time, causing lower back pain and possible nerve compression.

5. Osteoarthritis: Osteoarthritis is a condition in which the cartilage that cushions the joints breaks down, causing pain, stiffness, and possible nerve compression. It can occur in the lower back and lead to numbness or tingling in the legs.

**Q4a.**

Based on your symptoms, it is possible that you have knee osteoarthritis. Knee osteoarthritis is a common condition in which the cartilage that cushions the knee joint wears down over time, causing pain, swelling, stiffness, and possible cracking sounds when moving the knee.

Here are five potential differential diagnoses to consider:

1. Rheumatoid arthritis: Rheumatoid arthritis is an autoimmune disorder that can affect the joints, including the knee. It can cause swelling, pain, and stiffness in the joint, and can lead to joint damage if left untreated.

2. Gout: Gout is a form of arthritis that occurs when uric acid crystals accumulate in the joint, causing inflammation, swelling, and pain.

3. Meniscus tear: A meniscus tear is a common injury that can occur in the knee joint, causing pain, swelling, stiffness, and possible clicking or popping sounds when moving the knee.

4. Bursitis: Bursitis is a condition in which the bursae, small fluid-filled sacs that cushion the joints, become inflamed. It can cause pain, swelling, and stiffness in the knee joint.

5. Tendinitis: Tendinitis is a condition in which the tendons that attach muscles to bones become inflamed, causing pain and stiffness in the affected area. It can occur in the knee and cause pain and swelling.

**Q4c.**

Based on your symptoms, it is possible that you have Knee Osteoarthritis. Knee Osteoarthritis is a degenerative joint disease that occurs when the cartilage in the knee joint breaks down, leading to pain, stiffness, and swelling.

Some of the common symptoms of knee osteoarthritis include:

• Knee pain

• Stiffness in the knee joint, especially after sitting or lying down for a prolonged period

• Swelling in the knee joint

• Cracking or popping sounds when bending the knee

• Reduced range of motion in the knee joint

However, a proper diagnosis can only be made by a medical professional after a physical examination and diagnostic testing, such as X-rays or an MRI. Other potential causes of these symptoms include knee injuries, meniscus tears, rheumatoid arthritis, and bursitis, among others.

**Q5a.**

Based on your symptoms, it is possible that you have Hip Osteoarthritis. Hip Osteoarthritis is a degenerative joint disease that occurs when the cartilage in the hip joint breaks down, leading to pain, stiffness, and swelling.

Some of the common symptoms of hip osteoarthritis include:

• Hip pain

• Stiffness in the hip joint, especially after sitting or lying down for a prolonged period

• Swelling in the hip joint

• Cracking or popping sounds when moving the hip joint

• Reduced range of motion in the hip joint

Here are five potential differential diagnoses to consider:

1. Bursitis: Bursitis is a condition in which the bursae, small fluid-filled sacs that cushion the joints, become inflamed. It can cause pain, swelling, and stiffness in the hip joint.

2. Hip labral tear: A hip labral tear is an injury to the cartilage that lines the hip socket, which can cause pain, stiffness, and a clicking or catching sensation in the hip joint.

3. Rheumatoid arthritis: Rheumatoid arthritis is an autoimmune disorder that can affect the joints, including the hip. It can cause swelling, pain, and stiffness in the joint, and can lead to joint damage if left untreated.

4. Avascular necrosis: Avascular necrosis is a condition in which the bone tissue in the hip joint dies due to a lack of blood supply, leading to pain and stiffness in the hip joint.

5. Hip fracture: A hip fracture is a serious injury that can occur as a result of a fall or other trauma, causing severe hip pain, swelling, and difficulty moving the affected leg.

**Q5c.**

Based on your symptoms, it is possible that you have Hip Osteoarthritis. Hip Osteoarthritis is a degenerative joint disease that occurs when the cartilage in the hip joint breaks down, leading to pain, stiffness, and swelling.

Some of the common symptoms of hip osteoarthritis include:

• Hip pain

• Stiffness in the hip joint, especially after sitting or lying down for a prolonged period

• Swelling in the hip joint

• Cracking or popping sounds when moving the hip joint

• Reduced range of motion in the hip joint

However, a proper diagnosis can only be made by a medical professional after a physical examination and diagnostic testing, such as X-rays or an MRI. Other potential causes of these symptoms include hip injuries, bursitis, hip labral tear, avascular necrosis, and hip fractures, among others.

**Day 3**

**Q1a.**

Based on your symptoms of tingling and pain in your fingers, especially at night, along with difficulty picking up small objects, a possible primary diagnosis could be carpal tunnel syndrome.

Carpal tunnel syndrome is a condition that occurs when the median nerve, which runs from the forearm into the hand, becomes compressed or squeezed at the wrist. This can result in tingling, pain, and weakness in the hand and fingers, particularly in the thumb, index, and middle fingers. The symptoms may be worse at night, and individuals may experience difficulty grasping small objects.

However, it is important to note that there are other conditions that can cause similar symptoms, which should be considered as differential diagnoses. Here are five potential differential diagnoses to consider:

1. Cubital tunnel syndrome: This condition occurs when the ulnar nerve, which runs from the neck to the hand, becomes compressed at the elbow. It can cause similar symptoms to carpal tunnel syndrome, such as tingling, pain, and weakness in the hand and fingers.

2. Peripheral neuropathy: This is a condition that affects the peripheral nerves, which transmit signals between the central nervous system and the rest of the body. It can cause symptoms such as tingling, numbness, and pain in the hands and feet.

3. Rheumatoid arthritis: This is a chronic autoimmune disorder that affects the joints, causing inflammation and pain. It can also cause symptoms such as tingling and weakness in the hands.

4. Multiple sclerosis: This is a neurological disorder that affects the central nervous system. It can cause a wide range of symptoms, including tingling, numbness, and weakness in the hands and other parts of the body.

5. Thoracic outlet syndrome: This condition occurs when the nerves and blood vessels that run between the neck and the arm become compressed. It can cause symptoms such as tingling, pain, and weakness in the arm and hand.

It is important to consult with a healthcare provider for a proper diagnosis and treatment plan.

**Q1c.**

These symptoms may indicate a condition called carpal tunnel syndrome. Carpal tunnel syndrome occurs when the median nerve, which runs from the forearm to the hand through a narrow passage called the carpal tunnel, becomes compressed or pinched. This can cause tingling, pain, and weakness in the hand and fingers, particularly the thumb, index, and middle fingers.

Carpal tunnel syndrome can have several causes, including repetitive hand movements, wrist injuries, arthritis, pregnancy, and underlying medical conditions such as diabetes and thyroid dysfunction. Treatment options may include rest and activity modification, wrist splints, pain medication, and in some cases, surgery.

It is important to consult a healthcare professional if you suspect you have carpal tunnel syndrome or if your symptoms persist, as they can recommend the appropriate diagnosis and treatment plan.

**Q2a.**

Based on the symptoms you have described, the primary diagnosis that comes to mind is peripheral neuropathy. Peripheral neuropathy is a condition that affects the peripheral nerves, which are the nerves outside the brain and spinal cord that control our movements and sensations.

Peripheral neuropathy can have various causes, including:

1. Diabetes: high blood sugar levels can damage nerves throughout the body, causing peripheral neuropathy.

2. Autoimmune disorders: conditions such as lupus or rheumatoid arthritis can cause damage to nerves.

3. Vitamin deficiencies: deficiencies in vitamins B1, B6, B12, or E can lead to peripheral neuropathy.

4. Trauma or injury: physical injuries, such as a broken bone, can damage nerves.

5. Infections: certain viral or bacterial infections, such as Lyme disease or HIV, can lead to peripheral neuropathy.

Other potential differential diagnoses to consider in addition to peripheral neuropathy could include:

1. Carpal tunnel syndrome: a condition that causes numbness and tingling in the hand and arm due to compression of the median nerve.

2. Multiple sclerosis: a chronic autoimmune disorder that affects the central nervous system.

3. Parkinson's disease: a progressive disorder of the nervous system that affects movement.

4. Guillain-Barré syndrome: a rare autoimmune disorder that can cause rapid-onset muscle weakness, numbness, and tingling in the extremities.

5. Amyotrophic lateral sclerosis (ALS): a progressive neurodegenerative disorder that affects nerve cells in the brain and spinal cord, leading to muscle weakness and atrophy.

**Q2b.**

Based on the symptoms you have described, the possible disease that comes to mind is Carpal Tunnel Syndrome. It is a condition in which the median nerve, which runs from the forearm into the hand, becomes compressed at the wrist. This can cause numbness, tingling, weakness, or pain in the hand and wrist, and can make it difficult to do fine movements such as buttoning a shirt.

Carpal Tunnel Syndrome is often caused by repetitive hand movements, such as typing on a computer keyboard, or by medical conditions such as arthritis, diabetes, or thyroid dysfunction. Treatment options for Carpal Tunnel Syndrome may include wearing a wrist brace, taking pain relievers, undergoing physical therapy, or in severe cases, surgery.

**Q3a.**

Based on the symptoms you have described, the primary diagnosis that comes to mind is Lumbar Spinal Stenosis. It is a condition in which the spinal canal in the lower back narrows, causing pressure on the nerves that run through the spine. This can cause pain, numbness, and weakness in the buttocks and legs, especially when walking or standing upright, but may improve when leaning forward.

Other potential differential diagnoses to consider include:

1. Herniated Disc: a condition in which the gel-like center of a spinal disc protrudes into the spinal canal, causing pressure on the nerves and resulting in pain, numbness, and weakness in the legs and buttocks.

2. Sciatica: a condition caused by irritation or compression of the sciatic nerve, which runs from the lower back down through the legs, and may cause pain, tingling, or numbness in the buttocks and legs.

3. Osteoarthritis: a degenerative joint disease that can affect the lower back, causing pain and stiffness, and may also cause nerve compression if bone spurs develop.

4. Spondylolisthesis: a condition in which one vertebra slips forward over the one below it, causing pressure on the nerves and resulting in pain, numbness, and weakness in the legs and buttocks.

5. Piriformis Syndrome: a condition in which the piriformis muscle in the buttocks compresses the sciatic nerve, causing pain, tingling, or numbness in the buttocks and legs.

**Q4a.**

Based on the symptoms you have described, the primary diagnosis that comes to mind is Osteoarthritis of the Knee. Osteoarthritis is a degenerative joint disease that occurs when the cartilage that cushions the joint wears down over time, causing pain, swelling, and stiffness in the affected joint. Osteoarthritis of the knee is a common type of osteoarthritis that affects the knee joint.

Other potential differential diagnoses to consider include:

1. Rheumatoid Arthritis: an autoimmune disorder that causes inflammation in the joints, leading to pain, swelling, and stiffness, especially in the morning or after periods of inactivity.

2. Gout: a type of arthritis caused by the buildup of uric acid crystals in the joints, leading to sudden episodes of intense pain, swelling, and redness.

3. Meniscal Tear: a tear in the cartilage that cushions the knee joint, usually caused by sudden twisting or pivoting movements, leading to pain, swelling, and stiffness.

4. Bursitis: inflammation of the bursae, small fluid-filled sacs that cushion the knee joint, leading to pain, swelling, and stiffness.

5. Tendinitis: inflammation of the tendons that attach the muscles to the knee joint, leading to pain and stiffness, especially when moving the knee.

**Q4c.**

Based on the symptoms you have described, the possible disease that comes to mind is Osteoarthritis of the Knee. Osteoarthritis is a degenerative joint disease that occurs when the cartilage that cushions the joint wears down over time, causing pain, swelling, and stiffness in the affected joint. Osteoarthritis of the knee is a common type of osteoarthritis that affects the knee joint.

Other potential diseases to consider include:

1. Rheumatoid Arthritis: an autoimmune disorder that causes inflammation in the joints, leading to pain, swelling, and stiffness, especially in the morning or after periods of inactivity.

2. Gout: a type of arthritis caused by the buildup of uric acid crystals in the joints, leading to sudden episodes of intense pain, swelling, and redness.

3. Meniscal Tear: a tear in the cartilage that cushions the knee joint, usually caused by sudden twisting or pivoting movements, leading to pain, swelling, and stiffness.

4. Bursitis: inflammation of the bursae, small fluid-filled sacs that cushion the knee joint, leading to pain, swelling, and stiffness.

5. Patellofemoral Pain Syndrome: a condition in which the cartilage under the kneecap deteriorates, causing pain and stiffness in the front of the knee, especially when bending or squatting.

**Q5a**

Based on the symptoms you have described, the primary diagnosis that comes to mind is Osteoarthritis of the Hip. Osteoarthritis is a degenerative joint disease that occurs when the cartilage that cushions the joint wears down over time, causing pain, swelling, and stiffness in the affected joint. Osteoarthritis of the hip is a common type of osteoarthritis that affects the hip joint.

Other potential differential diagnoses to consider include:

1. Rheumatoid Arthritis: an autoimmune disorder that causes inflammation in the joints, leading to pain, swelling, and stiffness, especially in the morning or after periods of inactivity.

2. Bursitis: inflammation of the bursae, small fluid-filled sacs that cushion the hip joint, leading to pain, swelling, and stiffness.

3. Tendinitis: inflammation of the tendons that attach the muscles to the hip joint, leading to pain and stiffness, especially when moving the hip.

4. Hip Labral Tear: a tear in the labrum, the cartilage that surrounds the hip joint, leading to pain, stiffness, and clicking or catching sensations in the hip.

5. Avascular Necrosis: a condition in which the blood supply to the hip joint is disrupted, causing the bone to die and leading to pain, stiffness, and limited mobility.

**Q5c**

Based on the symptoms you have described, the possible disease that comes to mind is Osteoarthritis of the Hip. Osteoarthritis is a degenerative joint disease that occurs when the cartilage that cushions the joint wears down over time, causing pain, swelling, and stiffness in the affected joint. Osteoarthritis of the hip is a common type of osteoarthritis that affects the hip joint.

Other potential diseases to consider include:

1. Rheumatoid Arthritis: an autoimmune disorder that causes inflammation in the joints, leading to pain, swelling, and stiffness, especially in the morning or after periods of inactivity.

2. Bursitis: inflammation of the bursae, small fluid-filled sacs that cushion the hip joint, leading to pain, swelling, and stiffness.

3. Tendinitis: inflammation of the tendons that attach the muscles to the hip joint, leading to pain and stiffness, especially when moving the hip.

4. Hip Labral Tear: a tear in the labrum, the cartilage that surrounds the hip joint, leading to pain, stiffness, and clicking or catching sensations in the hip.

5. Avascular Necrosis: a condition in which the blood supply to the hip joint is disrupted, causing the bone to die and leading to pain, stiffness, and limited mobility.

**Day 4**

**Q1a**

Based on your symptoms of tingling and pain in the fingers, especially at night, along with difficulty picking up small objects, the primary diagnosis could be carpal tunnel syndrome. This is a condition that occurs when the median nerve, which runs from the forearm to the hand, becomes compressed or squeezed as it passes through the wrist. Other symptoms may include weakness in the hand or a tendency to drop objects.

Here are five potential differential diagnoses:

1. Peripheral neuropathy: This is a condition that affects the nerves outside of the brain and spinal cord, causing symptoms such as tingling, numbness, and pain in the hands and feet. It can be caused by a variety of factors, including diabetes, alcohol abuse, and certain medications.

2. Rheumatoid arthritis: This is an autoimmune disease that affects the joints, causing pain, stiffness, and swelling. It can also cause tingling and numbness in the hands and fingers.

3. Thoracic outlet syndrome: This is a condition that occurs when the blood vessels or nerves that run from the neck to the arm become compressed or squeezed. Symptoms may include tingling, pain, and weakness in the arms and hands.

4. Cubital tunnel syndrome: This is a condition that occurs when the ulnar nerve, which runs from the elbow to the hand, becomes compressed or irritated. Symptoms may include tingling, numbness, and pain in the fingers.

5. Multiple sclerosis: This is a neurological condition that affects the central nervous system, causing symptoms such as tingling, numbness, and weakness in the limbs. While it is less common than the other conditions listed here, it is important to consider as a potential differential diagnosis.

**Q1c**

Based on your symptoms of tingling and pain in the fingers, especially at night, along with difficulty picking up small objects, the most likely diagnosis is carpal tunnel syndrome. This is a condition that occurs when the median nerve, which runs from the forearm to the hand, becomes compressed or squeezed as it passes through the wrist. Other symptoms may include weakness in the hand or a tendency to drop objects. If left untreated, carpal tunnel syndrome can lead to permanent nerve damage. It is important to seek medical attention for an accurate diagnosis and appropriate treatment.

**Q2a**

Based on your symptoms of numbness in the hands, difficulty with fine movements, and an unsteady walk (especially when going downstairs), the primary diagnosis could be multiple sclerosis (MS). This is a neurological condition that affects the central nervous system, causing symptoms such as numbness, weakness, and difficulty with coordination.

Here are five potential differential diagnoses:

1. Diabetic neuropathy: This is a type of nerve damage that can occur in people with diabetes. It often affects the hands and feet and can cause symptoms such as numbness, tingling, and weakness.

2. Peripheral neuropathy: This is a condition that affects the nerves outside of the brain and spinal cord, causing symptoms such as numbness, tingling, and weakness in the hands and feet. It can be caused by a variety of factors, including diabetes, alcohol abuse, and certain medications.

3. Guillain-Barre syndrome: This is a rare but serious autoimmune disorder that can cause numbness, tingling, and weakness in the limbs. It can progress rapidly and may require hospitalization.

4. Cervical spondylosis: This is a condition that occurs when the discs in the neck degenerate, causing compression of the spinal cord or nerve roots. Symptoms can include numbness, tingling, and weakness in the arms and hands.

5. Parkinson's disease: This is a progressive neurological condition that affects movement, causing symptoms such as tremors, stiffness, and difficulty with balance and coordination. While it is less likely to present with numbness and tingling, it is important to consider as a potential differential diagnosis.

**Q2b**

Based on your symptoms of numbness in the hands and difficulty with fine movements, the most likely diagnosis is carpal tunnel syndrome. This is a condition that occurs when the median nerve, which runs from the forearm to the hand, becomes compressed or squeezed as it passes through the wrist. Other symptoms may include weakness in the hand or a tendency to drop objects. If left untreated, carpal tunnel syndrome can lead to permanent nerve damage. It is important to seek medical attention for an accurate diagnosis and appropriate treatment, which may include wrist splinting, medication, or in severe cases, surgery.

**Q3b**

Based on your symptoms of lower back pain, numbness, and pain in the buttocks and calves that increases with walking and improves with leaning forward, the primary diagnosis could be spinal stenosis. This is a condition that occurs when the spinal canal narrows, putting pressure on the spinal cord and nerves.

Here are five potential differential diagnoses:

1. Herniated disc: This is a condition that occurs when the soft material inside a disc in the spine pushes out through a tear in the outer layer, putting pressure on the spinal cord and nerves. Symptoms may include lower back pain and numbness or tingling in the legs.

2. Sciatica: This is a condition that occurs when the sciatic nerve, which runs from the lower back down through the legs, becomes compressed or irritated. Symptoms may include lower back pain, numbness, and pain or weakness in the legs.

3. Osteoarthritis: This is a type of arthritis that occurs when the cartilage in the joints breaks down, causing pain and stiffness. It can affect the spine and cause symptoms such as lower back pain.

4. Spondylolisthesis: This is a condition that occurs when one vertebra in the spine slips forward over the vertebra below it, causing pressure on the spinal cord and nerves. Symptoms may include lower back pain and numbness or weakness in the legs.

5. Piriformis syndrome: This is a condition that occurs when the piriformis muscle in the buttocks presses on the sciatic nerve, causing pain, numbness, and tingling in the buttocks and legs.

**Q4a**

Based on your symptoms of knee swelling, pain when walking, stiffness, and cracking sounds when bending the knee, the primary diagnosis could be osteoarthritis of the knee. This is a degenerative joint disease that causes the cartilage in the knee joint to wear down over time, leading to pain and stiffness.

Here are five potential differential diagnoses:

1. Rheumatoid arthritis: This is a chronic autoimmune disorder that causes inflammation in the joints, leading to pain, swelling, and stiffness. It can affect any joint in the body, including the knee.

2. Gout: This is a type of arthritis that occurs when uric acid crystals build up in the joints, causing inflammation and pain. It commonly affects the big toe, but can also affect the knee.

3. Knee bursitis: This is a condition that occurs when the bursae, small fluid-filled sacs that cushion the knee joint, become inflamed. It can cause swelling, pain, and stiffness in the knee.

4. Meniscal tear: This is a tear in the cartilage that cushions the knee joint, usually caused by twisting or turning the knee. Symptoms may include swelling, pain, and stiffness, and may also include a feeling of instability in the knee.

5. Ligament injury: This is a common knee injury that can occur when the knee is twisted or bent too far. Symptoms may include swelling, pain, and instability in the knee, and may also include a popping or snapping sensation at the time of injury.

**Q4c**

Based on your symptoms of knee swelling, pain when walking, stiffness, and cracking sounds when bending the knee, the most likely diagnosis is osteoarthritis of the knee. This is a degenerative joint disease that can affect any joint in the body, but commonly affects weight-bearing joints such as the knee.

In osteoarthritis, the cartilage that cushions the joint wears down over time, causing the bones to rub against each other and leading to pain, swelling, and stiffness. Other symptoms may include a limited range of motion in the knee joint, and the sensation of grinding or cracking when moving the joint.

It is important to seek medical attention for an accurate diagnosis and appropriate treatment, which may include pain medication, physical therapy, and, in severe cases, joint replacement surgery.

**Q5a**

Based on your symptoms of hip pain when walking, stiffness, and cracking sounds when moving the hip, the primary diagnosis could be osteoarthritis of the hip joint. This is a degenerative joint disease that can cause the cartilage that cushions the hip joint to wear down over time, leading to pain and stiffness.

Here are five potential differential diagnoses:

1. Hip labral tear: This is a tear in the cartilage that surrounds the hip joint, usually caused by injury or overuse. Symptoms may include pain, stiffness, and a clicking or locking sensation in the hip joint.

2. Bursitis: This is a condition that occurs when the bursae, small fluid-filled sacs that cushion the hip joint, become inflamed. It can cause pain and stiffness in the hip.

3. Tendinitis: This is a condition that occurs when a tendon, the tissue that connects muscles to bones, becomes inflamed. It can cause pain and stiffness in the hip, especially during movement.

4. Hip impingement: This is a condition that occurs when the bones in the hip joint rub against each other, causing pain and damage to the joint. It can cause pain and stiffness in the hip, especially during movement.

5. Avascular necrosis: This is a condition that occurs when the blood supply to the hip joint is disrupted, causing the bone tissue to die. It can cause pain and stiffness in the hip, especially during movement, and may require surgery to treat.

**Q5c**

Based on your symptoms of hip swelling, pain when walking, stiffness, and cracking sounds when moving the hip, the most likely diagnosis is osteoarthritis of the hip joint. This is a degenerative joint disease that can cause the cartilage that cushions the hip joint to wear down over time, leading to pain and stiffness.

It is important to seek medical attention for an accurate diagnosis and appropriate treatment, which may include pain medication, physical therapy, and, in severe cases, joint replacement surgery. Other possible causes of hip pain and swelling should also be considered and ruled out, such as bursitis, tendinitis, hip impingement, or avascular necrosis.

**Day 5**

**Q1a**

Based on the symptoms you described, it's possible that you are experiencing Carpal Tunnel Syndrome (CTS), a condition that occurs when the median nerve, which runs from your forearm into your hand, becomes compressed at the wrist. This can cause pain, tingling, and numbness in your fingers, especially at night. Other symptoms may include weakness in the affected hand and difficulty performing tasks that require fine motor skills.

Here are five potential differential diagnoses that your doctor may consider:

1. Peripheral neuropathy - A condition in which the nerves that carry messages to and from the brain and spinal cord to the rest of the body are damaged or diseased. This can cause numbness, tingling, and pain in the hands and feet, as well as weakness and loss of coordination.

2. Raynaud's disease - A condition in which the blood vessels in the fingers and toes spasm, causing a temporary interruption of blood flow. This can cause numbness, tingling, and pain in the affected areas.

3. Rheumatoid arthritis - An autoimmune disorder that causes inflammation in the joints. This can lead to pain, stiffness, and swelling in the hands and fingers, as well as difficulty with fine motor skills.

4. Dupuytren's contracture - A condition in which the tissue beneath the skin in the palm of the hand thickens and tightens, causing the fingers to curl inward. This can make it difficult to pick up small objects.

5. Trigger finger - A condition in which the tendon that allows you to bend your finger becomes inflamed and swollen, making it difficult to move the affected finger.

It's important to consult with a healthcare professional to receive an accurate diagnosis and appropriate treatment.

**Q1c**

Based on the symptoms you described, it is possible that you are experiencing Carpal Tunnel Syndrome (CTS). CTS occurs when the median nerve, which runs from your forearm into your hand, becomes compressed at the wrist, leading to pain, tingling, and numbness in your fingers, particularly at night. Other symptoms may include weakness in the affected hand and difficulty performing tasks that require fine motor skills, such as picking up small objects. It is important to consult with a healthcare professional to receive an accurate diagnosis and appropriate treatment.

Regenerate response

**Q2a**

Based on the symptoms you described, it is possible that you are experiencing a neurological condition that affects your peripheral nerves or spinal cord. A primary diagnosis cannot be given without a full medical evaluation, but here are five potential differential diagnoses that your doctor may consider:

1. Peripheral Neuropathy: A condition that affects the peripheral nerves, causing numbness, tingling, and weakness in the hands and feet. This can also affect fine motor movements and coordination, making it difficult to button a shirt or handle small objects.

2. Multiple Sclerosis (MS): A chronic autoimmune disease that affects the central nervous system, causing a range of symptoms such as numbness or tingling in the hands and feet, difficulty with fine motor movements, and unsteady walking.

3. Amyotrophic Lateral Sclerosis (ALS): A progressive neurodegenerative disease that affects nerve cells in the brain and spinal cord, leading to muscle weakness, including in the hands and feet.

4. Cervical Spondylosis: A condition that affects the cervical spine, causing wear and tear on the bones and cartilage of the neck. This can lead to nerve compression and symptoms such as numbness in the hands, difficulty with fine motor movements, and unsteady walking.

5. Parkinson's Disease: A neurodegenerative disorder that affects the nervous system and can cause tremors, stiffness, and difficulty with movement, including fine motor skills like buttoning a shirt.

It is important to consult with a healthcare professional to receive an accurate diagnosis and appropriate treatment. They may recommend imaging tests, nerve conduction studies, or other tests to help diagnose the underlying cause of your symptoms.

**Q2b**

Based on the symptoms you described, it is possible that you are experiencing peripheral neuropathy. Peripheral neuropathy is a condition that affects the peripheral nerves, causing numbness, tingling, and weakness in the hands and feet. It can also affect fine motor movements and coordination, making it difficult to handle small objects, such as buttoning a shirt. There are many potential causes of peripheral neuropathy, including diabetes, alcoholism, vitamin deficiencies, and certain medications. It is important to consult with a healthcare professional to receive an accurate diagnosis and appropriate treatment. They may recommend imaging tests, nerve conduction studies, or other tests to help diagnose the underlying cause of your symptoms.

**Q3b**

Based on the symptoms you described, it is possible that you are experiencing a condition called spinal stenosis. Spinal stenosis is a narrowing of the spaces within the spine, which can put pressure on the nerves that travel through the spine, leading to pain and numbness in the buttocks and legs. Symptoms often worsen with standing or walking and can improve with leaning forward, as this position can help relieve pressure on the affected nerves. However, a primary diagnosis cannot be given without a full medical evaluation. Here are five potential differential diagnoses that your doctor may consider:

1. Herniated Disc: A herniated disc occurs when the soft center of a spinal disc pushes through a crack in the tougher exterior casing, putting pressure on the nerves in the spine and causing pain, numbness, and weakness in the buttocks and legs.

2. Sciatica: Sciatica is a condition that occurs when the sciatic nerve, which runs from the lower back down through the legs, becomes compressed or irritated. This can cause pain, numbness, and weakness in the buttocks and legs.

3. Piriformis Syndrome: Piriformis syndrome occurs when the piriformis muscle in the buttocks compresses the sciatic nerve, leading to pain, numbness, and tingling in the buttocks and legs.

4. Osteoarthritis: Osteoarthritis is a degenerative joint disease that can affect the spine, causing pain and stiffness in the lower back and legs.

5. Spinal Tumor: A spinal tumor is an abnormal growth of cells in the spine that can put pressure on the nerves, causing pain, numbness, and weakness in the lower back and legs.

It is important to consult with a healthcare professional to receive an accurate diagnosis and appropriate treatment. They may recommend imaging tests, nerve conduction studies, or other tests to help diagnose the underlying cause of your symptoms.

**Q4a**

Based on the symptoms you described, it is possible that you are experiencing a knee injury or condition. A primary diagnosis cannot be given without a full medical evaluation, but here are five potential differential diagnoses that your doctor may consider:

1. Knee Osteoarthritis: Knee osteoarthritis is a degenerative joint disease that can cause swelling, stiffness, and pain in the knee joint. It can also cause cracking or popping sounds when bending the knee.

2. Meniscal Tear: A meniscal tear occurs when the cartilage in the knee joint is damaged, causing pain, swelling, and stiffness in the knee. It can also cause popping or clicking sensations when bending the knee.

3. Knee Bursitis: Knee bursitis is a condition that occurs when the bursae (small fluid-filled sacs) in the knee become inflamed, causing pain and swelling in the knee joint.

4. Patellofemoral Pain Syndrome: Patellofemoral pain syndrome is a condition that causes pain in the front of the knee, particularly when walking, running, or climbing stairs. It can also cause cracking or popping sounds when bending the knee.

5. Rheumatoid Arthritis: Rheumatoid arthritis is an autoimmune disease that can affect the knee joint, causing pain, swelling, stiffness, and deformity in the joint.

It is important to consult with a healthcare professional to receive an accurate diagnosis and appropriate treatment. They may recommend imaging tests, such as X-rays or an MRI, or other tests to help diagnose the underlying cause of your symptoms.

**Q4c**

Based on the symptoms you described, it is possible that you are experiencing knee osteoarthritis. Knee osteoarthritis is a degenerative joint disease that can cause swelling, stiffness, and pain in the knee joint. It can also cause cracking or popping sounds when bending the knee. Other symptoms may include decreased range of motion and difficulty walking. Knee osteoarthritis is a common condition, particularly in older adults and those with a history of knee injuries.

However, a primary diagnosis cannot be given without a full medical evaluation. It is important to consult with a healthcare professional to receive an accurate diagnosis and appropriate treatment. They may recommend imaging tests, such as X-rays or an MRI, or other tests to help diagnose the underlying cause of your symptoms. Treatment options may include pain management, physical therapy, or in severe cases, surgery.

**Q5a**

Based on the symptoms you described, it is possible that you are experiencing a hip injury or condition. A primary diagnosis cannot be given without a full medical evaluation, but here are five potential differential diagnoses that your doctor may consider:

1. Hip Osteoarthritis: Hip osteoarthritis is a degenerative joint disease that can cause pain, stiffness, and limited range of motion in the hip joint. It can also cause cracking or popping sounds when moving the hip.

2. Hip Labral Tear: A hip labral tear occurs when the labrum (a ring of cartilage in the hip joint) is damaged, causing pain and stiffness in the hip. It can also cause popping or clicking sensations when moving the hip.

3. Bursitis: Hip bursitis is a condition that occurs when the bursae (small fluid-filled sacs) in the hip become inflamed, causing pain and stiffness in the hip joint.

4. Hip Impingement: Hip impingement occurs when the bones of the hip joint rub against each other, causing pain and stiffness in the hip. It can also cause popping or clicking sensations when moving the hip.

5. Fracture: A hip fracture is a break in the bones of the hip joint, which can cause severe pain and difficulty moving the hip.

It is important to consult with a healthcare professional to receive an accurate diagnosis and appropriate treatment. They may recommend imaging tests, such as X-rays or an MRI, or other tests to help diagnose the underlying cause of your symptoms. Treatment options may include pain management, physical therapy, or in severe cases, surgery.

**Q5c**

Based on the symptoms you described, it is possible that you are experiencing a hip injury or condition. A primary diagnosis cannot be given without a full medical evaluation, but here are some potential causes that your doctor may consider:

1. Hip osteoarthritis: This is a degenerative joint disease that can cause pain, stiffness, and limited range of motion in the hip joint. It can also cause cracking or popping sounds when moving the hip.

2. Trochanteric bursitis: This is a condition where the bursa (a small fluid-filled sac) on the outside of the hip becomes inflamed, causing pain and swelling.

3. Hip labral tear: A labral tear occurs when the cartilage in the hip joint is damaged, causing pain and stiffness in the hip. It can also cause popping or clicking sensations when moving the hip.

4. Avascular necrosis: This is a condition where the blood supply to the hip joint is disrupted, causing damage to the bone and leading to pain, stiffness, and limited mobility.

5. Hip impingement: This is a condition where the bones of the hip joint rub against each other, causing pain and stiffness in the hip. It can also cause popping or clicking sensations when moving the hip.

It is important to consult with a healthcare professional to receive an accurate diagnosis and appropriate treatment. They may recommend imaging tests, such as X-rays or an MRI, or other tests to help diagnose the underlying cause of your symptoms. Treatment options may include pain management, physical therapy, or in severe cases, surgery.

**Author, TI**

**Day 1**

**Q1a**

Based on the symptoms you have described, the primary diagnosis could be carpal tunnel syndrome. Carpal tunnel syndrome occurs when the median nerve, which runs through the wrist, is compressed or pinched, resulting in tingling, pain, and weakness in the hand and fingers.

Here are five potential differential diagnoses:

Cubital tunnel syndrome: Similar to carpal tunnel syndrome, cubital tunnel syndrome involves compression of the ulnar nerve, which runs along the inner side of the elbow. This can cause pain, tingling, and weakness in the hand and fingers.

Peripheral neuropathy: Peripheral neuropathy is a condition in which the peripheral nerves, which transmit signals between the brain and spinal cord and the rest of the body, are damaged. This can cause a wide range of symptoms, including tingling, pain, and weakness in the hands and feet.

Rheumatoid arthritis: Rheumatoid arthritis is an autoimmune disorder that causes inflammation and damage to the joints. In addition to joint pain and stiffness, it can also cause tingling and weakness in the hands and fingers.

Thoracic outlet syndrome: Thoracic outlet syndrome involves compression of the nerves and blood vessels that pass through the thoracic outlet, a narrow space between the collarbone and the first rib. This can cause tingling, pain, and weakness in the arms and hands.

Multiple sclerosis: Multiple sclerosis is a neurological disorder that affects the central nervous system, causing a wide range of symptoms including tingling, numbness, and weakness in the limbs. While hand and finger symptoms are not typically the primary symptoms of multiple sclerosis, they can occur as part of the condition.

**Q1c**

These symptoms may indicate a condition called Carpal Tunnel Syndrome (CTS). CTS occurs when the median nerve, which runs from the forearm to the hand through a narrow passageway called the carpal tunnel, is compressed or squeezed at the wrist. This can lead to tingling, numbness, weakness, or pain in the hand and fingers, especially at night or in the morning.

CTS is commonly caused by repetitive hand and wrist movements, such as typing or using a computer mouse, but it can also be caused by other factors such as pregnancy, arthritis, or obesity.

It is important to see a doctor if you are experiencing these symptoms to confirm the diagnosis and receive appropriate treatment, which may include rest, hand and wrist exercises, wearing a splint, or in severe cases, surgery.

**Q2a**

I'm sorry to hear that you are experiencing these symptoms. However, as an AI language model, I cannot make medical diagnoses. It is crucial to consult with a healthcare professional who can evaluate your symptoms and provide a proper diagnosis.

That being said, based on the symptoms you described, there are several potential conditions that could be causing them. Here are five differential diagnoses to consider:

Carpal Tunnel Syndrome: This condition occurs when the median nerve, which runs from the forearm to the palm of the hand, is compressed. This can cause numbness, tingling, and weakness in the hand.

Multiple Sclerosis: This is a chronic autoimmune disease that affects the central nervous system, including the brain, spinal cord, and optic nerves. It can cause a range of symptoms, including numbness and tingling in the hands and feet, difficulty with fine motor movements, and problems with balance and coordination.

Parkinson's Disease: This is a neurodegenerative disorder that affects movement. It can cause tremors, stiffness, and difficulty with balance and coordination.

Diabetic Neuropathy: This condition can occur in people with diabetes and is caused by damage to the nerves. It can cause numbness, tingling, and weakness in the hands and feet.

Vitamin B12 Deficiency: This is a condition caused by a lack of vitamin B12 in the body. It can cause a range of symptoms, including numbness and tingling in the hands and feet, difficulty with fine motor movements, and problems with balance and coordination.

**Q2b**

Numbness in the hands and difficulty with fine movements could be symptoms of several medical conditions. One possible condition is carpal tunnel syndrome. This occurs when the median nerve, which runs from the forearm to the hand, is compressed as it passes through the carpal tunnel in the wrist. This can cause numbness, tingling, and weakness in the hand and fingers, particularly in the thumb, index, and middle fingers.

Another possible condition is peripheral neuropathy, which is damage to the peripheral nerves that connect the brain and spinal cord to the rest of the body. This can cause numbness, tingling, and weakness in the hands and feet, as well as difficulty with fine movements.

There are also other conditions that can cause these symptoms, such as cervical radiculopathy, which is a pinched nerve in the neck that can cause pain, numbness, and weakness in the arms and hands. It's important to see a doctor to get a proper diagnosis and treatment plan.

**Q3b**

Based on the symptoms you have described, it is possible that you are experiencing sciatica. Sciatica is a condition caused by irritation or compression of the sciatic nerve, which runs from the lower back down to the legs. The most common symptoms of sciatica include pain, numbness, and tingling in the lower back, buttocks, legs, or feet, as well as difficulty walking or standing for long periods of time.

Here are five potential differential diagnoses for your symptoms:

Lumbar spinal stenosis: This is a condition where the spinal canal narrows, putting pressure on the spinal cord and nerves. Symptoms include lower back pain, leg pain, and numbness, which may worsen when standing or walking.

Herniated disc: This occurs when the soft center of a spinal disc pushes through a crack in the outer layer, causing nerve compression and pain. Symptoms may include lower back pain, leg pain, and numbness or tingling in the legs.

Piriformis syndrome: This is a condition where the piriformis muscle in the buttocks compresses the sciatic nerve, causing pain and numbness in the buttocks and legs.

Spondylolisthesis: This is a condition where one vertebra slips forward over another, causing lower back pain and nerve compression. Symptoms may include leg pain and numbness.

Degenerative disc disease: This is a condition where the spinal discs degenerate, causing pain and nerve compression. Symptoms may include lower back pain and leg pain or numbness.

It is important to note that these are only potential differential diagnoses and a proper diagnosis would require a medical evaluation from a licensed healthcare professional.

**Q4a**

Based on your symptoms, it is possible that you may have a knee condition or injury, such as arthritis, bursitis, tendinitis, or a meniscus tear. A more specific diagnosis would require a physical examination and possibly imaging tests, such as an X-ray or MRI. It is important to see a medical professional for a proper diagnosis and treatment plan.

Here are five potential differential diagnoses for your knee symptoms:

Osteoarthritis: This is a degenerative joint disease that can cause swelling, stiffness, and pain in the knee, especially after periods of inactivity or overuse.

Rheumatoid arthritis: This is an autoimmune disease that can affect the joints, causing inflammation, stiffness, and pain in the knee and other areas.

Patellofemoral pain syndrome: This is a condition that affects the patella (kneecap) and the surrounding area, causing pain, swelling, and a feeling of grinding or cracking in the knee.

Anterior cruciate ligament (ACL) injury: This is a common knee injury that can occur during sports or other activities, causing pain, swelling, and instability in the knee.

Baker's cyst: This is a fluid-filled sac that can form behind the knee, causing swelling and pain, especially when the knee is bent.

**Q4c**

There are several possible conditions that could cause knee swelling, pain, stiffness, and cracking sounds. Some of the most common conditions include:

Osteoarthritis: This is a degenerative joint disease that occurs when the cartilage that cushions the joints wears down over time. Symptoms include joint pain, stiffness, and swelling, as well as a cracking or popping sound when moving the joint.

Rheumatoid arthritis: This is an autoimmune disease that causes inflammation and damage to the joints. Symptoms include joint pain, stiffness, and swelling, as well as fatigue and fever.

Gout: This is a type of arthritis that occurs when uric acid crystals build up in the joints. Symptoms include sudden attacks of intense pain, swelling, and redness in the joint.

Tendinitis: This is an inflammation of the tendon, which is the tissue that connects muscle to bone. Symptoms include pain and swelling in the affected area.

Bursitis: This is an inflammation of the bursae, which are small fluid-filled sacs that cushion the joints. Symptoms include pain, swelling, and stiffness in the affected joint.

It is important to see a doctor to determine the specific cause of your knee symptoms and to receive appropriate treatment.

**Q5a**

I'm sorry to hear that you're experiencing hip pain and stiffness. As an AI language model, I cannot provide a definitive diagnosis, and it is important to consult with a healthcare professional for an accurate evaluation. However, based on your symptoms, one possible primary diagnosis is osteoarthritis.

Here are five potential differential diagnoses for hip pain and stiffness:

Bursitis: Inflammation of the bursae, small fluid-filled sacs that cushion the hip joint.

Hip labral tear: A tear in the labrum, the cartilage that lines the rim of the hip socket.

Tendinitis: Inflammation of the tendons that attach muscles to bones in the hip joint.

Hip impingement syndrome: A condition where the hip bones are abnormally shaped, causing them to rub against each other and lead to hip pain and stiffness.

Rheumatoid arthritis: An autoimmune disorder that can cause joint pain, stiffness, and swelling in the hip joint, as well as other joints in the body.

**Q5c**

It is not possible to diagnose a specific disease based on these symptoms alone, as they can be indicative of several different conditions. However, hip swelling, pain, stiffness, and cracking sounds may be signs of hip osteoarthritis, which is a degenerative joint disease that occurs when the cartilage in the hip joint wears down over time. Other potential causes of these symptoms include bursitis, tendinitis, labral tears, or other types of arthritis such as rheumatoid arthritis or psoriatic arthritis.

It's important to consult with a healthcare provider, who can perform a physical examination and potentially order imaging tests to determine the underlying cause of your symptoms and recommend appropriate treatment options.

**Day 2**

**Q1a**

Based on your symptoms of tingling and pain in the fingers, especially at night, and difficulty picking up small objects, it is possible that you may be experiencing carpal tunnel syndrome. Carpal tunnel syndrome occurs when there is pressure on the median nerve that runs through the carpal tunnel in the wrist. This can cause symptoms such as pain, tingling, and weakness in the fingers, particularly the thumb, index, and middle fingers.

However, it is important to note that there can be other conditions that can cause similar symptoms. Here are five potential differential diagnoses:

Cubital tunnel syndrome: Similar to carpal tunnel syndrome, this condition affects the ulnar nerve at the elbow rather than the median nerve at the wrist. Symptoms may include tingling and pain in the ring and little fingers, as well as weakness in the hand.

Thoracic outlet syndrome: This condition occurs when there is compression of the nerves and blood vessels that pass through the thoracic outlet, a narrow space between the collarbone and the first rib. Symptoms can include pain, tingling, and weakness in the hand and arm.

Peripheral neuropathy: This condition is characterized by damage to the nerves that carry messages to and from the brain and spinal cord. Symptoms can include pain, tingling, and numbness in the hands and feet.

Rheumatoid arthritis: This is an autoimmune disease that can cause joint pain, swelling, and stiffness. It can also cause inflammation in the tendons and ligaments of the hands, leading to symptoms such as pain and difficulty gripping.

Multiple sclerosis: This is a neurological condition that can cause a range of symptoms, including tingling and weakness in the hands and feet. Other symptoms may include muscle stiffness, difficulty with coordination and balance, and vision problems.

It is important to note that this list is not exhaustive and that a proper diagnosis can only be made after a thorough medical examination and appropriate diagnostic tests. Therefore, it is recommended that you see a healthcare professional for an accurate diagnosis and appropriate treatment.

Modified Q1c

The symptoms you describe could be related to carpal tunnel syndrome, which is a condition that affects the hand and wrist.

Carpal tunnel syndrome is caused by compression of the median nerve, which runs through a narrow passageway in the wrist called the carpal tunnel. When this nerve becomes compressed, it can lead to tingling, numbness, and pain in the hand and fingers, especially at night.

People with carpal tunnel syndrome may also have difficulty grasping small objects or performing fine motor tasks with their hands. The condition is often associated with repetitive motions of the hand and wrist, such as typing on a keyboard or using a mouse, but it can also be caused by other factors such as pregnancy, arthritis, or a wrist injury.

If you suspect that you may have carpal tunnel syndrome, it's important to see a healthcare professional for an accurate diagnosis and appropriate treatment. In some cases, conservative treatments like splinting, stretching, or modifying activities can be effective in managing symptoms. In more severe cases, surgery may be recommended to relieve pressure on the nerve.

**Q2a**

I'm sorry to hear about your symptoms. It's important to note that I am an AI language model and not a licensed medical professional. Therefore, I cannot provide a definitive diagnosis. However, I can provide some information that may be helpful to you.

Based on your symptoms, the primary diagnosis that comes to mind is peripheral neuropathy. Peripheral neuropathy refers to damage or dysfunction of the peripheral nerves that transmit signals between the central nervous system and the rest of the body, such as the hands and feet.

Here are five potential differential diagnoses to consider:

Carpal tunnel syndrome: This condition occurs when the median nerve in the wrist is compressed, leading to numbness, tingling, and weakness in the hands.

Multiple sclerosis: MS is a chronic autoimmune disorder that affects the central nervous system, leading to a variety of symptoms, including numbness, weakness, and difficulty with coordination and balance.

Guillain-Barre syndrome: This rare autoimmune disorder occurs when the immune system attacks the peripheral nerves, leading to weakness, numbness, and tingling in the hands and feet.

Diabetic neuropathy: This type of neuropathy is caused by diabetes and can lead to numbness, tingling, and weakness in the hands and feet.

Cervical radiculopathy: This condition occurs when the nerves in the neck become compressed, leading to numbness, tingling, and weakness in the arms and hands.

It's important to seek medical attention from a licensed healthcare professional to determine the underlying cause of your symptoms and to receive appropriate treatment.

**Q2b**

The symptoms you are describing could be indicative of a few different conditions, but one possibility is carpal tunnel syndrome (CTS). CTS is a common condition that occurs when the median nerve, which runs from the forearm into the hand, becomes compressed or squeezed as it passes through the narrow space in the wrist called the carpal tunnel.

When the median nerve is compressed, it can cause numbness, tingling, and weakness in the hand and fingers, especially the thumb, index, and middle fingers. These symptoms may be more noticeable at night or after using the affected hand for repetitive tasks such as typing or using a computer mouse.

In addition to difficulty with fine movements, people with CTS may also have difficulty gripping objects, dropping objects, or experiencing a weak grasp. In some cases, CTS can be managed with rest, splinting, and physical therapy, while more severe cases may require surgery to relieve the compression on the median nerve.

However, other conditions such as peripheral neuropathy or cervical spondylosis can also cause similar symptoms. Therefore, it is essential to consult with a doctor or a medical professional to receive a proper diagnosis and treatment.

**Q3b**

Based on the symptoms you've described, your condition may be related to a problem with your sciatic nerve, which is the largest nerve in your body that runs from your lower back down to your feet.

A primary diagnosis would be sciatica, which is a term used to describe pain that radiates along the path of the sciatic nerve, typically affecting one side of the body. The pain is often described as shooting or burning and can be accompanied by numbness, tingling, or weakness in the affected leg or foot.

However, other conditions can also cause similar symptoms, so a thorough evaluation is necessary to determine the exact cause. Some potential differential diagnoses to consider include:

Herniated disc: A condition where the soft material inside a spinal disc protrudes through a crack in the tough outer layer, putting pressure on the nerves.

Spinal stenosis: A narrowing of the spinal canal, which puts pressure on the nerves and can cause symptoms similar to sciatica.

Piriformis syndrome: A condition where the piriformis muscle in the buttocks irritates the sciatic nerve, causing pain and numbness.

Degenerative disc disease: A condition where the discs in the spine lose their cushioning ability, leading to nerve compression and pain.

Spondylolisthesis: A condition where a vertebra in the spine slips out of place, causing nerve compression and pain.

It is important to seek medical attention from a healthcare provider to get an accurate diagnosis and appropriate treatment for your symptoms.

**Q4a**

I'm sorry to hear that you're experiencing discomfort in your knee. However, as an AI language model, I am not qualified to provide medical diagnoses. It is important to consult a healthcare professional for a proper diagnosis and treatment.

That being said, here are five potential differential diagnoses that could cause knee swelling, pain, stiffness, and cracking:

Osteoarthritis: A common form of arthritis that can cause pain, swelling, stiffness, and cracking sounds in the knee joint.

Rheumatoid arthritis: An autoimmune disorder that can cause joint pain, swelling, and stiffness, and may affect multiple joints, including the knee.

Meniscus tear: A tear in the cartilage of the knee joint can cause pain, swelling, and stiffness in the knee, especially when walking or bending.

Bursitis: Inflammation of the bursae (fluid-filled sacs) around the knee joint can cause pain, swelling, and stiffness.

Gout: A type of arthritis that occurs when there is a buildup of uric acid crystals in the joints, including the knee, causing sudden onset of pain, swelling, and stiffness.

Again, it is important to consult a healthcare professional for an accurate diagnosis and proper treatment.

**Q4c**

There are several conditions that can cause knee swelling, pain, stiffness, and cracking sounds when bending. One of the most common causes is osteoarthritis, which is a degenerative joint disease that affects the cartilage in the knee joint.

Other possible causes include rheumatoid arthritis, gout, knee bursitis, meniscal tears, ligament injuries, and patellofemoral pain syndrome. To determine the underlying cause of your symptoms, it is important to consult with a medical professional who can perform a physical examination and may recommend imaging studies such as X-rays, MRI, or ultrasound.

Based on the results of the examination and tests, the doctor can provide an accurate diagnosis and recommend appropriate treatment options, such as physical therapy, pain management, anti-inflammatory medications, or surgery.

**Q5a**

As an AI language model, I cannot provide a definitive medical diagnosis. However, based on your description, your symptoms could be indicative of several possible conditions.

A primary diagnosis could be osteoarthritis of the hip joint. This is a condition where the cartilage in the hip joint gradually wears away, causing pain and stiffness in the hip.

Here are five potential differential diagnoses to consider:

Bursitis: This is an inflammation of the fluid-filled sacs (bursae) that cushion the hip joint. It can cause pain and stiffness in the hip.

Hip labral tear: This is a tear in the ring of cartilage (labrum) that surrounds the hip joint. It can cause pain, stiffness, and a clicking or catching sensation in the hip.

Avascular necrosis: This is a condition where the bone tissue in the hip joint dies due to a lack of blood supply. It can cause pain and stiffness in the hip, as well as a limited range of motion.

Rheumatoid arthritis: This is a chronic inflammatory condition that can affect multiple joints, including the hip. It can cause pain, stiffness, and swelling in the affected joint.

Piriformis syndrome: This is a condition where the piriformis muscle in the buttock compresses the sciatic nerve, causing pain and tingling in the hip and leg.

It's important to consult with a healthcare professional for a proper diagnosis and treatment plan.

**Q5c**

Hip swelling and pain can have various causes, and it is not possible to provide a diagnosis without a medical examination. However, the symptoms you described are commonly associated with osteoarthritis, a condition in which the cartilage that cushions the joints wears down over time.

Osteoarthritis can cause pain, stiffness, and swelling in the hip joint, especially when you walk or engage in other activities that put pressure on the joint. The cracking sound you hear could be due to the roughening of the joint surface or the formation of bone spurs.

Other possible causes of hip pain and swelling include bursitis, tendinitis, and hip labral tears. It is essential to consult a medical professional to obtain a proper diagnosis and treatment plan. Your doctor may perform a physical examination, take X-rays or other imaging tests, and recommend a course of treatment based on the underlying cause of your symptoms.

**Day 3**

**Q1a**

Based on your symptoms, the primary diagnosis that comes to mind is carpal tunnel syndrome. This condition occurs when the median nerve, which runs from your forearm into your hand, becomes compressed at the wrist. This can cause pain, numbness, and tingling in the fingers, especially at night or in the morning. It can also make it difficult to grasp small objects.

Here are five potential differential diagnoses:

Cubital tunnel syndrome: This condition occurs when the ulnar nerve, which runs from your neck down to your hand, becomes compressed at the elbow. This can cause pain, tingling, and weakness in the hand and fingers.

Peripheral neuropathy: This is a broad term that refers to damage to the peripheral nerves, which can cause tingling, pain, and numbness in the hands and feet.

Raynaud's phenomenon: This is a condition in which the blood vessels in the fingers and toes narrow in response to cold or stress, causing pain, numbness, and tingling.

Rheumatoid arthritis: This is a chronic autoimmune disorder that causes inflammation and pain in the joints, including those in the hands and fingers.

Multiple sclerosis: This is a neurological disorder that can cause a wide range of symptoms, including tingling and numbness in the hands and fingers. However, it is usually accompanied by other neurological symptoms as well, such as vision problems or difficulty with balance and coordination.

**Q1c**

The symptoms you describe of tingling and pain in the fingers, particularly at night, along with difficulty picking up small objects, are commonly associated with carpal tunnel syndrome. This condition occurs when the median nerve, which runs from the forearm to the hand, becomes compressed as it passes through the carpal tunnel in the wrist.

Carpal tunnel syndrome can be caused by a variety of factors, including repetitive hand motions, injury, or underlying medical conditions such as diabetes or rheumatoid arthritis. Treatment options may include resting the affected hand, wearing a wrist splint, performing exercises to strengthen the hand and wrist, and in some cases, surgery.

It's important to see a healthcare professional for a proper diagnosis and treatment plan. They can assess your symptoms and perform tests, such as nerve conduction studies, to confirm if you have carpal tunnel syndrome or another condition.

**Q2a**

As an AI language model, I cannot provide a specific medical diagnosis. However, based on your symptoms, it is possible that you have a neurological condition that affects the nerves in your hands and feet. Some possible primary diagnoses and differential diagnoses are:

Primary diagnosis: Peripheral Neuropathy

Differential diagnoses:

Carpal Tunnel Syndrome: a condition caused by the compression of the median nerve in the wrist that can cause numbness, tingling, and weakness in the hand.

Multiple Sclerosis: a chronic autoimmune disease that affects the central nervous system and can cause numbness, weakness, and difficulty with coordination and balance.

Guillain-Barre Syndrome: a rare autoimmune disorder that affects the peripheral nerves and can cause numbness, weakness, and paralysis.

Amyotrophic Lateral Sclerosis (ALS): a progressive neurological disease that affects the nerve cells responsible for controlling voluntary muscles and can cause muscle weakness, atrophy, and difficulty with fine movements.

Parkinson's Disease: a progressive neurological disorder that affects movement and can cause tremors, rigidity, and difficulty with coordination and balance.

**Q2b**

The symptoms you described, including numbness in the hands and difficulty with fine movements, could be indicative of several different conditions, including carpal tunnel syndrome, peripheral neuropathy, or even multiple sclerosis.

Carpal tunnel syndrome is a condition that occurs when the median nerve, which runs from the forearm to the hand, becomes compressed as it passes through a narrow passageway called the carpal tunnel. This compression can cause numbness, tingling, and weakness in the hand and fingers.

Peripheral neuropathy refers to a group of disorders that affect the peripheral nerves, which transmit messages between the central nervous system (the brain and spinal cord) and the rest of the body. Symptoms of peripheral neuropathy can include numbness, tingling, and weakness in the hands and feet, as well as difficulty with fine motor skills.

Multiple sclerosis is a chronic autoimmune disease that affects the central nervous system. It can cause a wide range of symptoms, including numbness and tingling in the hands and feet, as well as weakness, difficulty with coordination and balance, and problems with vision and speech.

It's important to see a healthcare provider for an accurate diagnosis and appropriate treatment.

**Q3b**

Based on the symptoms you described, your condition may be related to spinal nerve compression, which is often caused by conditions such as a herniated disc or spinal stenosis. This condition is also known as lumbar radiculopathy or sciatica.

However, a definitive diagnosis requires a thorough medical evaluation by a qualified healthcare professional, including a physical exam, medical history, and diagnostic tests such as X-rays, MRI, or CT scans.

Here are five potential differential diagnoses to consider:

Piriformis syndrome: a condition where the piriformis muscle in the buttocks compresses the sciatic nerve, causing similar symptoms to those of lumbar radiculopathy.

Sacroiliac joint dysfunction: a condition where the sacroiliac joint in the pelvis is inflamed or damaged, causing lower back and leg pain.

Degenerative disc disease: a condition where the spinal discs between the vertebrae deteriorate, causing lower back pain and potentially radiating leg pain.

Spinal tumors: rare tumors that can compress spinal nerves and cause similar symptoms to lumbar radiculopathy.

Spinal infections: rare infections that can cause inflammation and compression of spinal nerves, leading to similar symptoms.

**Q4a**

As an AI language model, I am not a licensed healthcare professional and cannot diagnose medical conditions. However, based on the symptoms you described, it is possible that you are experiencing knee osteoarthritis.

Osteoarthritis is a condition where the cartilage in the knee joint wears away over time, causing pain, swelling, stiffness, and cracking sounds. It is more common in older adults but can also occur due to injuries or genetic factors.

Here are five potential differential diagnoses to consider:

Rheumatoid arthritis: This is an autoimmune disease that can cause joint pain, swelling, stiffness, and damage. It can affect multiple joints, including the knee, and may cause fatigue and fever.

Gout: Gout is a type of arthritis caused by the buildup of uric acid crystals in the joint, leading to inflammation, pain, and swelling. It typically affects the big toe, but can also affect the knee.

Bursitis: Bursitis is the inflammation of a fluid-filled sac called a bursa, which helps cushion and lubricate the joint. It can cause pain and swelling in the knee, especially when walking or bending.

Meniscus tear: A meniscus tear is a common knee injury that can cause pain, swelling, and stiffness. It may also cause a popping or clicking sound when the knee is bent.

Ligament injury: An injury to the ligaments that support the knee, such as the anterior cruciate ligament (ACL), can cause pain, swelling, and instability. It may also cause a popping or tearing sensation at the time of injury.

**Q4c**

There are several conditions that can cause knee swelling, pain, stiffness, and cracking sounds when bending. Some possible causes include:

Osteoarthritis: This is a degenerative joint disease that can cause inflammation, pain, and stiffness in the knee joint. It occurs when the protective cartilage on the ends of bones wears down over time.

Rheumatoid arthritis: This is an autoimmune disorder that can cause swelling, pain, and stiffness in the joints, including the knee joint.

Knee injury: A knee injury such as a sprain, strain, or tear in the ligaments, tendons, or cartilage can cause swelling, pain, stiffness, and cracking sounds.

Meniscus tear: The meniscus is a piece of cartilage that cushions the knee joint. A tear in the meniscus can cause pain, swelling, stiffness, and a popping or clicking sound when you move your knee.

Bursitis: Bursitis is the inflammation of the small sacs of fluid (bursae) that cushion the knee joint. It can cause pain, swelling, and stiffness.

It's important to consult with a healthcare professional to determine the underlying cause of your symptoms and to develop an appropriate treatment plan.

**Q5a**

I'm sorry to hear that you're experiencing hip pain and stiffness. As an AI language model, I cannot provide a definitive diagnosis. However, based on your symptoms, it is possible that you may be experiencing osteoarthritis, which is a type of degenerative joint disease that commonly affects the hip joint.

Here are five potential differential diagnoses that your doctor may consider when evaluating your symptoms:

Rheumatoid arthritis: This is an autoimmune disorder that can cause pain, stiffness, and swelling in multiple joints, including the hip.

Bursitis: This is inflammation of the bursae, small fluid-filled sacs that cushion the joints. It can cause hip pain, stiffness, and a clicking or popping sensation.

Tendinitis: This is inflammation of the tendons that attach muscles to bones. It can cause pain and stiffness in the hip joint.

Hip labral tear: This is a tear in the ring of cartilage (labrum) that surrounds the hip joint. It can cause pain and stiffness in the hip, as well as a clicking or locking sensation.

Avascular necrosis: This is a condition that occurs when the blood supply to a bone is disrupted, causing the bone tissue to die. It can cause hip pain, stiffness, and a limited range of motion.

It's important to consult with a healthcare provider for a proper diagnosis and treatment plan. They may recommend imaging tests such as X-rays or MRI scans to help determine the cause of your symptoms.

**Q5c**

The symptoms you are experiencing could be due to various causes, such as osteoarthritis, rheumatoid arthritis, hip labral tear, or bursitis.

Osteoarthritis is a condition in which the cartilage that cushions the joints breaks down, causing pain, stiffness, and swelling. Rheumatoid arthritis is an autoimmune disorder that causes inflammation in the joints, leading to pain and stiffness.

A hip labral tear is an injury to the ring of cartilage that surrounds the hip socket, which can cause pain, stiffness, and a locking or catching sensation in the hip joint. Bursitis is an inflammation of the fluid-filled sacs (bursae) that cushion the joints, causing pain and swelling.

It is important to consult with a healthcare provider to determine the exact cause of your symptoms and receive appropriate treatment. Your provider may perform a physical examination, imaging tests such as X-rays or MRI, or blood tests to diagnose the underlying cause of your hip pain and swelling.

**Day 4**

**Q1a**

It is difficult to provide a specific diagnosis without conducting a thorough medical examination, but based on the symptoms you described, it is possible that you are experiencing carpal tunnel syndrome. This is a condition that occurs when the median nerve, which runs from the forearm into the hand, becomes compressed as it passes through the wrist.

Here are five potential differential diagnoses to consider:

Cubital tunnel syndrome: This is a condition in which the ulnar nerve, which runs from the shoulder to the hand, becomes compressed at the elbow. Symptoms can include tingling and pain in the fingers, especially the pinky and ring fingers.

Rheumatoid arthritis: This is an autoimmune disease that can cause joint pain, swelling, and stiffness. It can affect the hands and fingers, and can cause difficulty picking up small objects.

Peripheral neuropathy: This is a condition in which the nerves outside of the brain and spinal cord are damaged, which can cause tingling and pain in the hands and feet.

Multiple sclerosis: This is a neurological disorder that can cause a wide range of symptoms, including tingling and numbness in the hands and feet.

Thoracic outlet syndrome: This is a condition in which the nerves and blood vessels that pass through the neck and shoulder become compressed, which can cause tingling and pain in the arms and hands.

It is important to see a doctor or healthcare professional for a proper diagnosis and treatment. They may refer you to a specialist such as a neurologist or hand surgeon.

**Q1c**

The symptoms you are experiencing, including tingling and pain in your fingers, difficulty picking up small objects, and worsening of symptoms at night, are commonly associated with carpal tunnel syndrome.

Carpal tunnel syndrome occurs when the median nerve, which runs from the forearm to the hand, becomes compressed or squeezed as it travels through the wrist. This can happen due to repetitive motions of the wrist or hand, injury, arthritis, or other underlying health conditions.

If you are experiencing these symptoms, it is important to see a healthcare professional for an accurate diagnosis and treatment plan. Your doctor may recommend wearing a splint or brace to immobilize the wrist, physical therapy, or medication to reduce inflammation and pain. In some cases, surgery may be necessary to relieve the pressure on the median nerve.

**Q2a**

Based on the symptoms you described, the primary diagnosis that comes to mind is peripheral neuropathy, which is a disorder of the peripheral nerves that can cause numbness, tingling, and weakness in the hands and feet.

Five potential differential diagnoses for these symptoms could include:

Carpal tunnel syndrome: This is a common condition that affects the median nerve as it travels through the wrist, causing numbness and tingling in the hand.

Multiple sclerosis: This is a chronic autoimmune disorder that affects the central nervous system, causing a wide range of symptoms including numbness, weakness, and difficulty with coordination.

Parkinson's disease: This is a neurodegenerative disorder that affects movement and can cause tremors, stiffness, and difficulty with fine motor skills.

Vitamin B12 deficiency: This can cause nerve damage, leading to symptoms such as numbness and tingling in the hands and feet, difficulty walking, and difficulty with fine motor skills.

Stroke: A stroke can cause sudden weakness, numbness, or paralysis on one side of the body, which can include the hands and feet, and may affect fine motor skills.

It's important to note that a proper diagnosis requires a thorough medical evaluation and testing by a qualified healthcare professional.

**Q2b**

The symptoms you are describing could be related to a number of different conditions, but one possibility is carpal tunnel syndrome. Carpal tunnel syndrome occurs when the median nerve, which runs from the forearm into the hand, is compressed as it passes through the wrist. This can cause numbness, tingling, and weakness in the hand, and can make it difficult to perform fine motor tasks like buttoning a shirt.

Other conditions that could cause similar symptoms include peripheral neuropathy, which is a general term for nerve damage that affects the peripheral nerves (the nerves outside the brain and spinal cord), and cervical radiculopathy, which is a condition that occurs when a nerve in the neck is compressed or irritated.

It's important to see a healthcare provider for an accurate diagnosis and appropriate treatment. They can perform a physical examination, take a medical history, and order any necessary tests to determine the underlying cause of your symptoms.

**Q3b**

As an AI language model, I cannot provide medical diagnosis, but based on the symptoms you provided, it is important to seek medical attention from a healthcare provider who can perform a physical examination and diagnostic tests to determine the underlying cause of your symptoms.

However, to assist you, here is some information on potential conditions that may cause similar symptoms:

Primary Diagnosis:

The symptoms you described are consistent with a condition called "Lumbar spinal stenosis". It is a common condition in older adults, which occurs when the spinal canal narrows and compresses the nerves that run through the lower back, buttocks, and legs. This can cause pain, numbness, and weakness in these areas.

Differential Diagnoses:

Other potential causes of similar symptoms include:

Herniated Disc: A herniated disc occurs when the soft, jelly-like center of a spinal disc pushes through a tear in the tougher outer layer, putting pressure on nearby nerves and causing pain and numbness.

Sciatica: Sciatica is a condition caused by compression of the sciatic nerve, which runs from the lower back down the back of each leg. It can cause pain, numbness, and tingling in the lower back, buttocks, and legs.

Piriformis Syndrome: Piriformis syndrome occurs when the piriformis muscle, which runs from the lower spine to the thighbone, compresses the sciatic nerve, causing pain and numbness in the buttocks and down the leg.

Spinal Tumor: A spinal tumor can cause similar symptoms to those of lumbar spinal stenosis, including pain, numbness, and weakness in the lower back, buttocks, and legs.

Osteoarthritis: Osteoarthritis is a degenerative joint disease that can affect the lower back and cause pain and stiffness. It can also cause the bones to compress the nerves leading to pain, numbness, and tingling in the legs.

Again, these are just some of the potential causes of your symptoms, and a healthcare provider would be best equipped to determine the underlying condition through a physical examination, medical history, and diagnostic tests.

**Q4a**

Based on the symptoms you described, the primary diagnosis could be knee osteoarthritis, which is a degenerative joint disease characterized by joint inflammation, pain, and stiffness. However, other conditions could also present with similar symptoms, and a proper diagnosis requires a physical exam, medical history, and diagnostic tests. Here are five potential differential diagnoses:

Rheumatoid arthritis: An autoimmune disorder that causes joint pain, swelling, and stiffness, particularly in the small joints of the hands and feet, but can also affect the knees.

Gout: A form of arthritis that results from the buildup of uric acid crystals in the joints, leading to severe pain, swelling, and stiffness, particularly in the big toe, but can also affect the knee.

Bursitis: An inflammation of the bursae, which are small fluid-filled sacs that cushion the knee joint, leading to pain, swelling, and stiffness around the knee.

Meniscus tear: A tear in the cartilage of the knee joint that can cause pain, swelling, and stiffness, particularly when bending or twisting the knee.

Knee ligament injury: A sprain or tear of one of the knee ligaments, such as the ACL or MCL, which can cause pain, swelling, and instability in the knee joint.

**Q4c**

There are several possible conditions that could cause the symptoms you're experiencing. One common condition is osteoarthritis, which occurs when the cartilage in your joints wears down over time, causing pain and stiffness. Other possible conditions include rheumatoid arthritis, gout, or a knee injury such as a meniscus tear or ligament sprain.

It's important to see a doctor for an accurate diagnosis and appropriate treatment. Your doctor may order imaging tests such as X-rays or an MRI to help identify the underlying cause of your knee pain and swelling. They may also recommend physical therapy, medications, or in severe cases, surgery.

**Q5a**

As an AI language model, I cannot provide a medical diagnosis, and I strongly advise seeking the advice of a licensed healthcare professional who can evaluate your symptoms and conduct appropriate tests. However, I can provide some information on potential causes of hip pain and stiffness.

Based on your symptoms, there could be several potential causes of your hip pain, such as:

Primary diagnosis:

Osteoarthritis: This is a common degenerative joint disease that causes inflammation, pain, and stiffness in the joints, including the hips. It is often accompanied by a cracking or popping sound when the joint is moved.

Differential diagnoses could include:

Rheumatoid arthritis: This is an autoimmune disease that can affect multiple joints, including the hips. It can cause stiffness, pain, and swelling.

Bursitis: Inflammation of the bursae (fluid-filled sacs that cushion the joints) can cause pain and stiffness in the hip.

Tendinitis: Inflammation of the tendons around the hip joint can cause pain and stiffness.

Hip labral tear: A tear in the labrum (a ring of cartilage that helps stabilize the hip joint) can cause pain, stiffness, and clicking or popping sensations.

Avascular necrosis: This is a condition in which the blood supply to the hip joint is disrupted, leading to bone death and joint damage. It can cause pain and stiffness, as well as a cracking or popping sound when the joint is moved.

Again, these are just some potential causes of hip pain and stiffness. A licensed healthcare professional would be able to evaluate your symptoms and provide a more accurate diagnosis.

**Q5c**

The symptoms you describe could be related to several different conditions, including arthritis, bursitis, tendonitis, or hip labral tear. It's difficult to determine the exact cause without a physical examination and diagnostic tests such as X-rays or MRI.

Arthritis is a common condition that causes inflammation and swelling in the joints. Osteoarthritis, rheumatoid arthritis, and psoriatic arthritis are the most common types that affect the hip joint.

Bursitis is another condition that can cause hip pain and swelling. It occurs when the bursae, small fluid-filled sacs that cushion the joint, become inflamed.

Tendonitis is inflammation of the tendons, the fibrous tissue that connects muscles to bones. It can also cause hip pain and stiffness.

Hip labral tear is a condition where the ring of cartilage that surrounds the hip joint, known as the labrum, is damaged or torn. It can cause pain, stiffness, and clicking or locking sensations.

It is recommended to see a doctor or a specialist such as a rheumatologist or an orthopedic surgeon for a proper evaluation and diagnosis. They will be able to determine the underlying cause of your symptoms and develop a treatment plan to alleviate your pain and improve your hip function.

**Day 5**

**Q1a**

Based on the symptoms you have described, it is possible that you are experiencing carpal tunnel syndrome. Carpal tunnel syndrome occurs when the median nerve, which runs from the forearm to the hand, becomes compressed or squeezed as it passes through the wrist.

Here are five potential differential diagnoses for your symptoms:

Peripheral neuropathy: This is a disorder of the peripheral nervous system that can cause numbness, tingling, and pain in the hands and feet.

Cubital tunnel syndrome: Similar to carpal tunnel syndrome, cubital tunnel syndrome occurs when the ulnar nerve, which runs from the shoulder to the hand, becomes compressed or irritated as it passes through the elbow.

Rheumatoid arthritis: This is a chronic autoimmune disorder that can cause joint pain, swelling, and stiffness in the hands and feet.

Dupuytren's contracture: This is a condition where the connective tissue in the palm of the hand thickens and tightens, causing the fingers to curl inward and making it difficult to straighten them.

Raynaud's disease: This is a disorder that affects the blood vessels in the fingers and toes, causing them to narrow and restrict blood flow, leading to pain, tingling, and numbness.

**Q1c**

The symptoms you have described, such as tingling and pain in the fingers, as well as difficulty picking up small objects, may be indicative of carpal tunnel syndrome (CTS). CTS is a common condition that occurs when the median nerve, which runs from the forearm into the hand, becomes compressed or pinched as it passes through a narrow passageway called the carpal tunnel in the wrist.

The symptoms of CTS can be mild to severe and can vary from person to person. In addition to tingling and pain in the fingers, other common symptoms include numbness, weakness, and a burning sensation in the affected hand or wrist. Symptoms are often worse at night or when performing activities that involve repetitive hand movements, such as typing or using tools.

If you suspect you have CTS, it is important to consult with a healthcare professional for an accurate diagnosis and treatment options. Treatment may include wrist splinting, medications, or, in severe cases, surgery.

**Q2a**

Based on your symptoms, a possible primary diagnosis is peripheral neuropathy. Peripheral neuropathy is a condition in which the nerves that carry information to and from the brain and spinal cord to the rest of the body become damaged or diseased, resulting in symptoms such as numbness, tingling, and weakness in the affected areas.

Here are five potential differential diagnoses:

Carpal tunnel syndrome: This is a condition that occurs when the median nerve, which runs from the forearm to the hand, is compressed as it passes through the wrist. Symptoms include numbness, tingling, and weakness in the hand, particularly in the thumb, index, and middle fingers.

Multiple sclerosis: This is a chronic autoimmune disorder that affects the central nervous system. Symptoms can include numbness, tingling, and weakness in the limbs, as well as difficulty with balance and coordination.

Parkinson's disease: This is a neurodegenerative disorder that primarily affects movement, with symptoms including tremors, stiffness, and difficulty with balance and coordination.

Guillain-Barre syndrome: This is a rare autoimmune disorder in which the immune system attacks the peripheral nerves, resulting in symptoms such as numbness, tingling, and weakness in the limbs.

Vitamin B12 deficiency: This is a condition in which the body doesn't have enough vitamin B12, which is essential for healthy nerve function. Symptoms can include numbness, tingling, and weakness in the limbs, as well as difficulty with balance and coordination.

**Q2b**

Numbness in the hands and difficulty with fine movements could be symptoms of various conditions, but one possible explanation is carpal tunnel syndrome (CTS).

CTS is a condition that occurs when the median nerve, which runs from the forearm into the hand, becomes compressed at the wrist. This can cause numbness, tingling, or weakness in the hand, as well as difficulty with fine movements like buttoning a shirt.

There are also other conditions that can cause similar symptoms, such as peripheral neuropathy or cervical radiculopathy, so it's important to see a doctor to get an accurate diagnosis and appropriate treatment.

**Q3a**

Based on the symptoms you described, it is possible that you are experiencing a herniated or bulging disc in your lower back, which can put pressure on the nerves that run down your legs. This condition is also known as sciatica.

Here are five potential differential diagnoses that may be considered:

Spinal stenosis: This is a narrowing of the spinal canal, which can compress the nerves that run through it and cause pain, weakness, or numbness in the legs.

Piriformis syndrome: This is a condition in which the piriformis muscle, which runs from the lower back to the hip joint, becomes tight or inflamed and compresses the sciatic nerve, causing similar symptoms to those of a herniated disc.

Osteoarthritis: This is a condition in which the cartilage in the joints breaks down, causing pain, stiffness, and sometimes numbness or weakness in the legs.

Spondylolisthesis: This is a condition in which a vertebra in the spine slips out of place and puts pressure on the nerves that run through the spinal canal.

Tumors: While rare, tumors or growths in the spine can cause similar symptoms to those of a herniated disc, including pain, numbness, and weakness in the legs.

It's important to note that only a qualified medical professional can provide an accurate diagnosis. If you are experiencing these symptoms, it's recommended that you seek medical attention as soon as possible.

**Q4a**

As an AI language model, I cannot provide a definitive diagnosis, and it's important to consult with a medical professional for proper evaluation and treatment. However, based on your symptoms, a possible primary diagnosis could be osteoarthritis of the knee.

Here are five potential differential diagnoses to consider:

Rheumatoid arthritis: A chronic autoimmune disease that affects the joints and causes swelling, stiffness, and pain.

Gout: A type of arthritis that occurs due to the buildup of uric acid crystals in the joint, causing severe pain and swelling.

Meniscal tear: A tear in the rubbery meniscus tissue that cushions the knee joint, which can cause swelling, pain, and stiffness.

Bursitis: Inflammation of the bursae, small fluid-filled sacs that cushion and lubricate the joints, causing pain and swelling.

Tendinitis: Inflammation of the tendons that attach muscles to bones, causing pain and stiffness in the affected area.

**Q4c**

There are several possible causes of knee swelling, pain, stiffness, and cracking sounds when bending the knee. One possible condition is osteoarthritis, which is a degenerative joint disease that can cause joint inflammation, cartilage damage, and bone spurs. Another possibility is rheumatoid arthritis, which is an autoimmune disorder that causes inflammation and damage to joints. Other potential causes include knee injury, bursitis, tendonitis, gout, and infection.

To determine the specific cause of your knee symptoms, you should see a healthcare professional, such as an orthopedist or rheumatologist. They may perform a physical exam and diagnostic tests, such as x-rays or blood tests, to help identify the underlying condition and develop an appropriate treatment plan.

**Q5a**

As an AI language model, I cannot diagnose medical conditions. However, I can provide you with some information that might be useful.

Hip pain and stiffness with a cracking sound can be associated with various medical conditions. Here are five potential differential diagnoses:

Osteoarthritis: A degenerative joint disease that affects the cartilage in joints, leading to pain, stiffness, and cracking sounds during movement.

Bursitis: Inflammation of the bursa, which are small sacs of fluid that cushion and lubricate the joints. Bursitis can cause pain, swelling, and stiffness in the hip joint.

Tendinitis: Inflammation of a tendon, which connects muscle to bone. Tendinitis can cause pain and stiffness in the hip joint, and may be associated with cracking sounds during movement.

Hip labral tear: A tear in the labrum, which is a ring of cartilage that surrounds the hip joint. A labral tear can cause pain, stiffness, and a clicking or locking sensation in the hip joint.

Avascular necrosis: A condition in which the blood supply to the hip joint is disrupted, leading to the death of bone tissue. This can cause pain, stiffness, and cracking sounds during movement.

It's important to see a doctor or healthcare provider to determine the underlying cause of your symptoms and receive appropriate treatment.

**Q5c**

Hip swelling, pain, stiffness, and cracking sounds can be symptoms of various conditions related to the hip joint. The most common causes of hip pain are arthritis, bursitis, and tendonitis.

Arthritis is a condition that causes inflammation and damage to the joints, and it can affect the hip joint. The two most common types of arthritis that affect the hip are osteoarthritis and rheumatoid arthritis. Osteoarthritis is a degenerative joint disease that occurs when the cartilage that cushions the joint breaks down over time. Rheumatoid arthritis is an autoimmune disease that causes inflammation and damage to the joints, including the hip joint.

Bursitis is inflammation of the bursa, which is a fluid-filled sac that cushions and lubricates the joints. Bursitis in the hip can cause pain, stiffness, and swelling.

Tendonitis is inflammation of the tendons, which are the thick cords that attach muscles to bones. Tendonitis in the hip can cause pain, stiffness, and limited mobility.

Other possible causes of hip pain and swelling include hip fractures, hip labral tears, and hip impingement syndrome. To determine the specific cause of your symptoms, it is best to consult a healthcare professional who can perform a physical exam and order any necessary imaging tests.

**Author, KT**

**Day 1**

**Q1a**

I'm not a doctor, but I can try to provide some general information. The symptoms you described, such as tingling, pain in the fingers (especially at night), and difficulty picking up small objects, could be indicative of a condition called carpal tunnel syndrome (CTS). CTS is a condition that occurs when the median nerve, which runs from the forearm to the hand, becomes compressed or squeezed at the wrist. It is commonly associated with repetitive hand movements, such as typing or using vibrating tools.

However, it's important to note that many conditions can cause similar symptoms. It would be best to consult with a healthcare professional for a proper diagnosis. They will evaluate your symptoms, conduct a physical examination, and may order additional tests if necessary. That said, here are five potential differential diagnoses to consider:

Peripheral neuropathy: This condition involves damage to the peripheral nerves, which can cause tingling, pain, and numbness in the hands and fingers. It can be caused by various factors such as diabetes, vitamin deficiencies, or nerve compression.

Cubital tunnel syndrome: Similar to carpal tunnel syndrome, cubital tunnel syndrome involves compression of the ulnar nerve at the elbow. It can lead to tingling, pain, and weakness in the fingers, especially the ring and little fingers.

Cervical radiculopathy: This condition occurs when a nerve root in the neck becomes compressed or irritated. It can cause pain, tingling, and weakness that radiate into the arms and fingers.

Rheumatoid arthritis: This chronic autoimmune disease primarily affects the joints, but it can also cause inflammation and nerve compression, leading to hand pain, swelling, and tingling.

Raynaud's disease: This condition is characterized by spasms of the blood vessels in response to cold or stress, resulting in reduced blood flow to the fingers. It can cause numbness, tingling, and pain in the fingers, often triggered by cold temperatures.

Remember, these are just potential differential diagnoses, and a healthcare professional is best equipped to provide an accurate diagnosis based on a comprehensive evaluation of your symptoms and medical history.

**Q1c**

The symptoms you described, such as tingling, pain (especially at night), and difficulty picking up small objects, are commonly associated with a condition called carpal tunnel syndrome (CTS). CTS occurs when the median nerve, which runs through a narrow passage in the wrist called the carpal tunnel, becomes compressed or squeezed. This can result in symptoms like numbness, tingling, pain, and weakness in the hand, particularly the thumb, index, middle, and ring fingers.

Carpal tunnel syndrome is often caused by repetitive hand and wrist movements, such as typing, using vibrating tools, or engaging in activities that require prolonged gripping. Other factors that may contribute to the development of CTS include wrist injuries, certain medical conditions (such as diabetes, rheumatoid arthritis, or hypothyroidism), hormonal changes (such as during pregnancy), and certain anatomical factors.

It's important to note that I'm an AI language model and not a healthcare professional, so I can't provide a definitive diagnosis. If you're experiencing these symptoms, it's recommended to consult with a healthcare provider who can evaluate your condition properly. They will consider your symptoms, medical history, and may conduct physical examinations or diagnostic tests to confirm the diagnosis and provide appropriate treatment options.

**Q2a**

I'm not a doctor, but I can provide some general information. The combination of symptoms you mentioned, including numbness in the hands, difficulty with fine movements, and an unsteady walk (particularly when going downstairs), could be indicative of a neurological condition. However, it's important to consult with a healthcare professional for a proper diagnosis. They will evaluate your symptoms, conduct a physical examination, and may order additional tests if necessary. That said, here are five potential differential diagnoses to consider:

Multiple Sclerosis (MS): MS is a chronic autoimmune disease that affects the central nervous system. It can cause a wide range of symptoms, including numbness or tingling in the hands, difficulties with coordination and fine movements, and problems with balance and walking.

Peripheral Neuropathy: Peripheral neuropathy refers to damage or dysfunction of the peripheral nerves. It can lead to symptoms such as numbness, tingling, and difficulties with fine motor skills. There are various causes for peripheral neuropathy, including diabetes, nutritional deficiencies, and certain medications.

Parkinson's Disease: Parkinson's disease is a progressive neurological disorder that affects movement. It can cause motor symptoms such as a shaky or unsteady walk, as well as difficulties with fine motor skills and dexterity.

Cervical Spinal Stenosis: Cervical spinal stenosis is a condition characterized by the narrowing of the spinal canal in the neck region. It can put pressure on the spinal cord and nerves, leading to symptoms like numbness in the hands, difficulties with fine movements, and an unsteady gait.

Vitamin B12 Deficiency: A deficiency in vitamin B12 can lead to neurological symptoms, including numbness or tingling in the hands, difficulty with fine movements, and gait disturbances.

These are just potential differential diagnoses, and a healthcare professional is best equipped to provide an accurate diagnosis based on a comprehensive evaluation of your symptoms, medical history, and any additional diagnostic tests that may be necessary.

**Q2b**

I'm not a doctor, but the combination of symptoms you described, including numbness in the hands and difficulty with fine movements, could potentially be associated with a condition called peripheral neuropathy. Peripheral neuropathy refers to damage or dysfunction of the peripheral nerves, which are responsible for transmitting signals between the central nervous system (brain and spinal cord) and the rest of the body.

There are various potential causes of peripheral neuropathy, including:

Diabetes: High blood sugar levels in diabetes can lead to nerve damage, resulting in symptoms such as numbness, tingling, and difficulties with fine motor skills.

Nutritional deficiencies: Deficiencies in certain vitamins (such as vitamin B12), minerals, or other nutrients can affect nerve health and contribute to peripheral neuropathy.

Autoimmune disorders: Conditions like rheumatoid arthritis, lupus, or Guillain-Barré syndrome can cause inflammation and damage to the peripheral nerves.

Infections: Certain infections, such as Lyme disease, HIV/AIDS, or hepatitis C, can lead to peripheral neuropathy as a complication.

Trauma or injury: Physical trauma or nerve compression due to repetitive motions or pressure on the nerves can result in peripheral neuropathy symptoms.

It's important to note that this is just a general overview, and a proper diagnosis can only be provided by a qualified healthcare professional. If you're experiencing these symptoms, I highly recommend consulting with a doctor who can evaluate your condition, conduct a thorough examination, and order any necessary tests to determine the underlying cause and provide appropriate treatment.

**Q3a**

I'm not a doctor, but based on the symptoms you described, it's possible that you may be experiencing a condition called sciatica. Sciatica refers to pain that radiates along the path of the sciatic nerve, which runs from the lower back down through the hips, buttocks, and legs. Sciatica is commonly caused by compression or irritation of the sciatic nerve, often due to a herniated disc or spinal stenosis in the lumbar (lower) spine.

However, it's important to consult with a healthcare professional for a proper diagnosis. They will consider your symptoms, medical history, and may conduct physical examinations or diagnostic tests to confirm the diagnosis and provide appropriate treatment options. That said, here are five potential differential diagnoses to consider:

Lumbar herniated disc: A herniated disc occurs when the soft center of a spinal disc pushes through a crack in the tougher outer layer, potentially compressing a nerve root and causing symptoms like lower back pain, numbness, and radiating pain down the leg.

Lumbar spinal stenosis: This condition involves the narrowing of the spinal canal in the lower back, which can put pressure on the nerves and lead to symptoms such as lower back pain, buttock pain, and leg pain that worsens with standing or walking.

Piriformis syndrome: The piriformis muscle, located in the buttock region, can sometimes irritate or compress the sciatic nerve, causing symptoms similar to sciatica, including buttock pain, numbness, and radiating pain down the leg.

Spinal tumors or infections: Rarely, tumors or infections in the spine can cause lower back pain and radiating symptoms. These conditions require medical attention for proper diagnosis and treatment.

Sacroiliac joint dysfunction: Dysfunction or inflammation of the sacroiliac joint, which connects the sacrum and pelvis, can cause lower back pain that may radiate to the buttocks and legs. The pain may worsen with walking or standing.

Remember, these are potential differential diagnoses, and a healthcare professional is best equipped to provide an accurate diagnosis based on a comprehensive evaluation of your symptoms, medical history, and any necessary tests.

**Q4a**

I'm not a doctor, but based on the symptoms you described, it's possible that you may be experiencing a knee condition such as osteoarthritis. Osteoarthritis is a degenerative joint disease that can cause swelling, pain, stiffness, and cracking sounds when moving the affected joint. It commonly occurs due to the gradual wear and tear of the protective cartilage in the knee joint.

However, it's important to consult with a healthcare professional for a proper diagnosis. They will consider your symptoms, medical history, and may conduct physical examinations or diagnostic tests to confirm the diagnosis and provide appropriate treatment options. That said, here are five potential differential diagnoses to consider:

Rheumatoid arthritis: Rheumatoid arthritis is an autoimmune disease that can affect multiple joints, including the knee. It can cause swelling, pain, stiffness, and joint deformity. Rheumatoid arthritis is characterized by inflammation and can affect other organs as well.

Meniscal tear: A meniscal tear is a common knee injury that can cause swelling, pain, and a feeling of stiffness. It may occur due to sudden twisting or direct trauma to the knee and is often associated with a popping or clicking sensation.

Bursitis: Bursitis is the inflammation of the fluid-filled sacs (bursae) that cushion the joints. In the knee, it can lead to swelling, pain, and stiffness. Bursitis can be caused by repetitive kneeling, overuse, or direct trauma.

Ligament injury: Injuries to the ligaments in the knee, such as an anterior cruciate ligament (ACL) tear or a medial collateral ligament (MCL) sprain, can cause swelling, pain, and instability. Ligament injuries often occur due to sudden twisting or direct impact to the knee.

Gout: Gout is a form of arthritis caused by the buildup of uric acid crystals in the joints. It commonly affects the big toe but can also affect the knee, causing sudden and severe pain, swelling, and redness.

Remember, these are potential differential diagnoses, and a healthcare professional is best equipped to provide an accurate diagnosis based on a comprehensive evaluation of your symptoms, medical history, and any necessary tests.

**Q4c**

I'm not a doctor, but based on the symptoms you described, it's possible that you may be experiencing a knee condition called patellofemoral pain syndrome (PFPS). PFPS is a common knee problem characterized by pain in the front of the knee, particularly around or behind the kneecap (patella).

PFPS often occurs due to factors such as overuse, improper alignment of the patella, muscle imbalances, or abnormal tracking of the kneecap. The pain is typically aggravated by activities that involve repetitive knee bending, such as walking, running, or going up and down stairs. Stiffness and cracking sounds (known as crepitus) when bending the knee can also be present.

However, it's important to consult with a healthcare professional for a proper diagnosis. They will consider your symptoms, conduct a physical examination, and may order additional tests if necessary to confirm the diagnosis and rule out other potential causes. Other possible conditions that could cause similar symptoms include:

Osteoarthritis: Osteoarthritis is a degenerative joint disease that can cause knee pain, swelling, stiffness, and cracking sounds. It typically occurs due to the gradual wear and tear of the knee joint.

Meniscal tear: A tear in the meniscus, which is a piece of cartilage in the knee, can cause pain, swelling, and a feeling of stiffness. It may be associated with clicking or locking sensations in the knee.

Ligament injury: Injuries to the ligaments of the knee, such as an anterior cruciate ligament (ACL) tear or a medial collateral ligament (MCL) sprain, can lead to knee pain, swelling, and instability.

Bursitis: Bursitis is inflammation of the fluid-filled sacs (bursae) around the knee joint. It can cause localized pain, swelling, and stiffness in the knee.

Rheumatoid arthritis: Rheumatoid arthritis is an autoimmune disease that can affect multiple joints, including the knee. It typically causes pain, swelling, stiffness, and joint deformity.

Remember, these are potential differential diagnoses, and a healthcare professional is best equipped to provide an accurate diagnosis based on a comprehensive evaluation of your symptoms, medical history, and any necessary tests.

**Q5a**

I'm not a doctor, but based on the symptoms you described, it's possible that you may be experiencing a hip condition called hip osteoarthritis. Hip osteoarthritis is a degenerative joint disease that occurs when the protective cartilage in the hip joint wears down over time, resulting in pain, stiffness, and sometimes cracking or popping sensations with movement.

However, it's important to consult with a healthcare professional for a proper diagnosis. They will consider your symptoms, medical history, and may conduct physical examinations or diagnostic tests to confirm the diagnosis and provide appropriate treatment options. That said, here are five potential differential diagnoses to consider:

Bursitis: Hip bursitis is inflammation of the bursae, small fluid-filled sacs that cushion the hip joint. It can cause pain, stiffness, and tenderness in the hip, especially with walking or prolonged periods of standing.

Hip labral tear: The labrum is a ring of cartilage that helps stabilize the hip joint. A tear in the labrum can cause hip pain, stiffness, and a sensation of catching or locking with certain movements.

Hip tendonitis: Tendonitis refers to inflammation of the tendons surrounding the hip joint. It can cause pain, stiffness, and difficulty with movement, especially during activities that involve repetitive hip motions.

Hip impingement: Hip impingement, also known as femoroacetabular impingement (FAI), occurs when the bones of the hip joint abnormally rub against each other. It can result in hip pain, stiffness, and a feeling of catching or clicking during movement.

Hip stress fracture: A stress fracture is a small crack in the bone that typically occurs due to overuse or repetitive stress. It can cause hip pain that worsens with weight-bearing activities.

These are potential differential diagnoses, and a healthcare professional is best equipped to provide an accurate diagnosis based on a comprehensive evaluation of your symptoms, medical history, and any necessary tests.

**Q5c**

I'm not a doctor, but based on the symptoms you described, it's possible that you may be experiencing a hip condition called hip osteoarthritis. Hip osteoarthritis is a degenerative joint disease that occurs when the protective cartilage in the hip joint wears down over time, resulting in pain, swelling, stiffness, and sometimes cracking or popping sensations with movement.

However, it's important to consult with a healthcare professional for a proper diagnosis. They will consider your symptoms, medical history, and may conduct physical examinations or diagnostic tests to confirm the diagnosis and provide appropriate treatment options.

Other potential conditions that could cause similar symptoms include:

Hip bursitis: Hip bursitis is inflammation of the bursae, small fluid-filled sacs that cushion the hip joint. It can cause pain, swelling, and stiffness in the hip, especially with walking or prolonged periods of standing.

Hip labral tear: The labrum is a ring of cartilage that helps stabilize the hip joint. A tear in the labrum can cause hip pain, swelling, stiffness, and a sensation of catching or locking with certain movements.

Rheumatoid arthritis: Rheumatoid arthritis is an autoimmune disease that can affect multiple joints, including the hip. It typically causes pain, swelling, stiffness, and joint deformity.

Avascular necrosis: Avascular necrosis, also known as osteonecrosis, occurs when the blood supply to the hip joint is disrupted, leading to the death of bone tissue. It can cause hip pain, swelling, stiffness, and difficulty walking.

Hip joint infection: Infections can occasionally affect the hip joint, resulting in pain, swelling, and stiffness. Infections can occur due to bacteria or other pathogens entering the joint space.

Remember, these are potential differential diagnoses, and a healthcare professional is best equipped to provide an accurate diagnosis based on a comprehensive evaluation of your symptoms, medical history, and any necessary tests.

**Day 2**

**Q1a**

I'm not a doctor, but I can provide you with some general information. The symptoms you're experiencing, such as tingling, pain in the fingers (particularly at night), and difficulty picking up small objects, could be indicative of various conditions affecting the hands and fingers. It's important to consult with a healthcare professional for a proper diagnosis. They will consider your medical history, conduct a physical examination, and possibly order diagnostic tests to determine the underlying cause of your symptoms.

However, here are five potential differential diagnoses that could explain your symptoms:

Carpal Tunnel Syndrome: This condition occurs when the median nerve, which runs through the carpal tunnel in the wrist, becomes compressed. It commonly causes tingling, pain, and numbness in the hand, particularly the thumb, index, middle, and half of the ring finger.

Cubital Tunnel Syndrome: Also known as ulnar nerve entrapment, this condition involves compression or irritation of the ulnar nerve near the elbow. Symptoms may include tingling, pain, and weakness in the ring finger and pinky finger.

Peripheral Neuropathy: This is a disorder affecting the peripheral nerves, often caused by conditions like diabetes, vitamin deficiencies, or autoimmune diseases. Peripheral neuropathy can lead to tingling, pain, and sensory abnormalities in the hands and feet.

Rheumatoid Arthritis: An autoimmune disease that primarily affects the joints, rheumatoid arthritis can cause inflammation and damage in the hands and fingers. Symptoms may include pain, swelling, stiffness, and difficulty with fine motor tasks.

Thoracic Outlet Syndrome: This condition involves the compression of nerves or blood vessels in the space between the collarbone and first rib. It can cause symptoms such as tingling, pain, and weakness in the fingers, hand, or arm.

Remember, these are just a few potential conditions that could explain your symptoms. It's important to consult a healthcare professional for an accurate diagnosis and appropriate treatment.

**Q1c**

I apologize for any confusion, but as an AI language model, I cannot provide specific medical diagnoses. The symptoms you've described, including tingling, pain (especially at night), and difficulty picking up small objects, can be associated with various medical conditions. It's important to consult with a qualified healthcare professional, such as a doctor or a neurologist, who can evaluate your symptoms, conduct a thorough examination, and possibly order diagnostic tests to determine the underlying cause.

Some potential causes for your symptoms may include:

Carpal Tunnel Syndrome: This occurs when the median nerve, which runs through a narrow passage in the wrist called the carpal tunnel, becomes compressed or irritated. It can cause tingling, pain, and difficulty with fine motor tasks.

Peripheral Neuropathy: This condition involves damage or dysfunction of the peripheral nerves, which can be caused by various factors such as diabetes, vitamin deficiencies, infections, or certain medications. Peripheral neuropathy can lead to tingling, pain, and sensory changes in the affected areas.

Nerve Compression: Compression of nerves in other areas of the arm, such as the ulnar nerve at the elbow or the radial nerve in the forearm, can cause similar symptoms.

Arthritis: Certain types of arthritis, such as rheumatoid arthritis or osteoarthritis, can affect the hands and fingers, leading to joint pain, inflammation, and difficulties with dexterity.

Raynaud's Disease: This condition causes narrowing of blood vessels in the extremities, particularly in response to cold temperatures or stress. It can result in pain, tingling, and color changes in the fingers.

Again, it's crucial to consult with a healthcare professional for an accurate diagnosis based on your specific symptoms, medical history, and a proper evaluation. They will be able to provide you with the most appropriate guidance and treatment options.

**Q2a**

I'm not a doctor, but I can provide you with some general information. The symptoms you're experiencing, including numbness in the hands, difficulty with fine movements, and an unsteady walk, can be associated with various medical conditions. It's important to consult with a healthcare professional for a proper diagnosis based on a thorough examination, medical history, and possibly additional tests.

That being said, here is a primary diagnosis and five potential differential diagnoses that could explain your symptoms:

Primary Diagnosis:

Peripheral Neuropathy: Peripheral neuropathy refers to damage or dysfunction of the peripheral nerves that transmit signals between the central nervous system (brain and spinal cord) and the rest of the body. It can cause numbness, tingling, and weakness in the hands and feet, affecting fine motor skills and balance.

Potential Differential Diagnoses:

Carpal Tunnel Syndrome: This condition involves compression of the median nerve in the wrist, resulting in symptoms such as numbness, tingling, and weakness in the hand.

Multiple Sclerosis (MS): MS is an autoimmune disease that affects the central nervous system. It can cause various neurological symptoms, including numbness, difficulties with coordination, and an unsteady gait.

Diabetic Neuropathy: Diabetes can lead to nerve damage, particularly in the hands and feet. Diabetic neuropathy can result in numbness, tingling, and loss of sensation in the affected areas.

Guillain-Barré Syndrome: This is a rare neurological disorder where the body's immune system mistakenly attacks the peripheral nerves. It often starts with weakness and numbness in the limbs and can progress to difficulties with motor function.

Cervical Spinal Stenosis: This condition involves the narrowing of the spinal canal in the neck region, putting pressure on the spinal cord and nerves. It can cause symptoms such as hand numbness, clumsiness, and difficulty with balance.

Please note that these are just possible explanations, and an accurate diagnosis can only be provided by a qualified healthcare professional. It's important to consult with a doctor who can evaluate your symptoms, conduct appropriate tests, and provide the necessary guidance for your specific situation.

Modified Q2b

I'm not a doctor, but based on the symptoms you've described, one possible explanation could be Carpal Tunnel Syndrome (CTS). Carpal Tunnel Syndrome occurs when the median nerve, which runs from the forearm into the hand, becomes compressed or squeezed at the wrist.

The symptoms of Carpal Tunnel Syndrome often include:

Numbness or tingling sensation in the thumb, index finger, middle finger, and half of the ring finger.

Difficulty with fine movements, such as buttoning a shirt or gripping small objects.

Weakness in the hand or a tendency to drop objects.

Discomfort or pain that may extend from the wrist to the forearm or even the upper arm.

It's important to note that these symptoms can also be caused by other conditions, so it's crucial to consult with a healthcare professional for an accurate diagnosis. They will consider your medical history, conduct a physical examination, and possibly order tests, such as nerve conduction studies, to confirm the diagnosis and rule out other potential causes.

Remember, only a qualified medical professional can provide a definitive diagnosis based on a comprehensive evaluation of your symptoms and medical history.

**Q3b**

I'm not a doctor, but based on the symptoms you've described, one possible explanation could be a condition called Lumbar Spinal Stenosis. Lumbar spinal stenosis refers to the narrowing of the spinal canal in the lower back, which can put pressure on the nerves that travel through that area.

The symptoms of lumbar spinal stenosis often include:

Pain in the lower back.

Numbness or pain in the buttocks and calves, which may radiate down the legs (referred to as sciatica).

Increased pain with walking or standing for prolonged periods, known as neurogenic claudication.

Relief of pain when leaning forward or sitting down, as these positions can temporarily alleviate pressure on the affected nerves.

However, it's important to note that other conditions can also cause similar symptoms, so it's crucial to consult with a healthcare professional for an accurate diagnosis. They will consider your medical history, conduct a physical examination, and possibly order imaging tests, such as an MRI, to confirm the diagnosis and rule out other potential causes.

Here are five potential differential diagnoses that could explain your symptoms:

Herniated Disc: A herniated or slipped disc occurs when the cushioning discs between the vertebrae in the spine become damaged, causing them to press on nearby nerves and lead to pain and numbness in the back and legs.

Degenerative Disc Disease: This condition involves the breakdown of the intervertebral discs in the spine over time, resulting in pain, numbness, and stiffness in the back and legs.

Spinal Arthritis: Arthritis of the spine, such as osteoarthritis or spondylosis, can cause pain, stiffness, and nerve compression in the lower back, leading to similar symptoms.

Piriformis Syndrome: The piriformis muscle, located in the buttocks, can sometimes irritate or compress the sciatic nerve, resulting in buttock pain, numbness, and radiating leg pain.

Peripheral Neuropathy: Peripheral neuropathy refers to nerve damage in the peripheral nervous system, which can cause pain, numbness, and tingling sensations in various areas of the body, including the lower back and legs.

Again, it's essential to consult with a healthcare professional for a proper diagnosis based on your specific symptoms, medical history, and a thorough evaluation. They will be able to provide you with the most accurate guidance and appropriate treatment options.

**Q4a**

I'm not a doctor, but based on the symptoms you've described, one possible explanation could be Knee Osteoarthritis. Knee osteoarthritis is a degenerative joint disease characterized by the breakdown of cartilage in the knee joint. It commonly causes swelling, pain, stiffness, and a cracking or grinding sensation when moving the knee.

The primary diagnosis, based on the information provided, could be Knee Osteoarthritis.

Here are five potential differential diagnoses that could also explain your symptoms:

Rheumatoid Arthritis: Rheumatoid arthritis is an autoimmune disease that can affect multiple joints, including the knees. It causes joint inflammation, pain, swelling, and stiffness. Unlike osteoarthritis, rheumatoid arthritis is a systemic condition that may involve other symptoms and affect other joints symmetrically.

Meniscal Tear: A meniscal tear is a common injury that can cause knee pain, swelling, and stiffness. It often occurs due to sudden twisting or a direct impact to the knee, and it may be associated with a feeling of instability or catching.

Bursitis: Knee bursitis refers to the inflammation of the bursae, which are small fluid-filled sacs that cushion the joints. It can cause swelling, pain, and limited mobility in the affected knee.

Gout: Gout is a form of arthritis caused by the buildup of uric acid crystals in the joints. It typically affects the big toe, but it can also involve the knee joint, leading to severe pain, swelling, redness, and warmth.

Ligamentous Injury: An injury to the ligaments of the knee, such as an anterior cruciate ligament (ACL) tear or sprain, can cause pain, swelling, instability, and difficulty walking or bearing weight on the affected knee.

Please keep in mind that these are just potential explanations, and an accurate diagnosis can only be provided by a qualified healthcare professional. It's important to consult with a doctor who can evaluate your symptoms, conduct appropriate tests or imaging, and provide the necessary guidance for your specific situation.

**Q4c**

Based on the symptoms you've described, one possible explanation could be Knee Osteoarthritis. Knee osteoarthritis is a degenerative joint disease characterized by the breakdown of cartilage in the knee joint. It commonly causes swelling, pain, stiffness, and a cracking or grinding sensation when moving the knee.

However, there are several other conditions that could also explain your symptoms, including:

Rheumatoid Arthritis: Rheumatoid arthritis is an autoimmune disease that can affect multiple joints, including the knees. It causes joint inflammation, pain, swelling, and stiffness. Unlike osteoarthritis, rheumatoid arthritis is a systemic condition that may involve other symptoms and affect other joints symmetrically.

Meniscal Tear: A meniscal tear is a common injury that can cause knee pain, swelling, and stiffness. It often occurs due to sudden twisting or a direct impact to the knee, and it may be associated with a feeling of instability or catching.

Bursitis: Knee bursitis refers to the inflammation of the bursae, which are small fluid-filled sacs that cushion the joints. It can cause swelling, pain, and limited mobility in the affected knee.

Gout: Gout is a form of arthritis caused by the buildup of uric acid crystals in the joints. It typically affects the big toe, but it can also involve the knee joint, leading to severe pain, swelling, redness, and warmth.

Ligamentous Injury: An injury to the ligaments of the knee, such as an anterior cruciate ligament (ACL) tear or sprain, can cause pain, swelling, instability, and difficulty walking or bearing weight on the affected knee.

Please keep in mind that these are just potential explanations, and an accurate diagnosis can only be provided by a qualified healthcare professional. It's important to consult with a doctor who can evaluate your symptoms, conduct appropriate tests or imaging, and provide the necessary guidance for your specific situation.

**Q5a**

I'm not a doctor, but based on the symptoms you've described, one possible explanation could be Hip Osteoarthritis. Hip osteoarthritis is a degenerative joint disease characterized by the breakdown of cartilage in the hip joint. It commonly causes pain, stiffness, and a cracking or grinding sensation when moving the hip. The pain is often felt in the groin, buttocks, or thigh, and it may worsen with activity such as walking.

The primary diagnosis, based on the information provided, could be Hip Osteoarthritis.

Here are five potential differential diagnoses that could also explain your symptoms:

Bursitis: Hip bursitis refers to the inflammation of the bursae, which are small fluid-filled sacs that cushion the joints. It can cause pain, tenderness, and stiffness in the hip, particularly on the outer side.

Hip Labral Tear: A labral tear is an injury to the ring of cartilage, known as the labrum, that lines the hip socket. It can cause hip pain, stiffness, and a catching or locking sensation during movement.

Tendinitis: Tendinitis refers to inflammation or irritation of the tendons around the hip joint. It can cause pain, tenderness, and limited range of motion in the hip.

Avascular Necrosis: Avascular necrosis, also known as osteonecrosis, is a condition in which the blood supply to the hip joint is disrupted, leading to the death of bone tissue. It can cause hip pain, stiffness, and limited mobility.

Hip Fracture: A fracture in the hip, such as a femoral neck fracture or a hip socket fracture, can cause severe hip pain, difficulty bearing weight on the affected leg, and limited mobility.

Please keep in mind that these are just potential explanations, and an accurate diagnosis can only be provided by a qualified healthcare professional. It's important to consult with a doctor who can evaluate your symptoms, conduct appropriate tests or imaging, and provide the necessary guidance for your specific situation.

Modified Q5c

I'm not a doctor, but based on the symptoms you've described, one possible explanation could be Hip Osteoarthritis. Hip osteoarthritis is a degenerative joint disease characterized by the breakdown of cartilage in the hip joint. It commonly causes pain, swelling, stiffness, and a cracking or grinding sensation when moving the hip. The pain is often felt in the groin, buttocks, or thigh, and it may worsen with activity such as walking.

However, there are other conditions that could also cause similar symptoms, so it's important to consult with a healthcare professional for an accurate diagnosis. Here are five potential differential diagnoses that could explain your symptoms:

Hip Bursitis: Hip bursitis refers to the inflammation of the bursae, which are small fluid-filled sacs that cushion the joints. It can cause hip pain, swelling, and stiffness, particularly on the outer side of the hip.

Hip Labral Tear: A labral tear is an injury to the ring of cartilage, known as the labrum, that lines the hip socket. It can cause hip pain, stiffness, and a catching or locking sensation during movement.

Avascular Necrosis: Avascular necrosis, also known as osteonecrosis, occurs when the blood supply to the hip joint is disrupted, leading to the death of bone tissue. It can cause hip pain, swelling, and limited range of motion.

Rheumatoid Arthritis: Rheumatoid arthritis is an autoimmune disease that can affect multiple joints, including the hip. It causes joint inflammation, pain, swelling, and stiffness. Unlike osteoarthritis, rheumatoid arthritis is a systemic condition that may involve other symptoms and affect other joints symmetrically.

Hip Fracture: A fracture in the hip, such as a femoral neck fracture or a hip socket fracture, can cause severe hip pain, swelling, difficulty bearing weight on the affected leg, and limited mobility.

Remember, it's important to consult with a healthcare professional who can evaluate your symptoms, conduct a physical examination, and possibly order imaging tests to provide an accurate diagnosis and recommend appropriate treatment options.

**Day3**

**Q1a**

I'm not a doctor, but I can provide you with some general information. The symptoms you described, such as tingling, pain, and difficulty picking up small objects, can be associated with various conditions. It's important to consult with a healthcare professional for an accurate diagnosis. However, I can give you a primary diagnosis and a list of five potential differential diagnoses based on the symptoms you mentioned:

Primary Diagnosis:

Carpal Tunnel Syndrome (CTS): This is a common condition characterized by compression of the median nerve as it passes through the wrist. It often leads to tingling, pain, and numbness in the fingers, particularly at night.

Potential Differential Diagnoses:

Peripheral Neuropathy: A condition involving damage to the peripheral nerves, which can cause tingling, pain, and loss of sensation in the hands and fingers.

Cubital Tunnel Syndrome: Compression of the ulnar nerve at the elbow, leading to similar symptoms as CTS, but affecting different fingers.

Thoracic Outlet Syndrome: Compression of nerves and blood vessels as they pass through the thoracic outlet (area between the neck and shoulder), resulting in hand and finger symptoms.

Rheumatoid Arthritis: An autoimmune disorder that can cause inflammation and joint pain, which may affect the fingers and hand dexterity.

Multiple Sclerosis: A chronic neurological condition that can cause various symptoms, including tingling, numbness, and weakness in the extremities.

Remember, these are just potential differential diagnoses, and only a qualified healthcare professional can accurately evaluate your symptoms and provide a diagnosis. It's essential to consult with a doctor for a proper evaluation and appropriate treatment.

**Q1c**

I'm not a doctor, but based on the symptoms you described, one possible disease that could be causing your symptoms is carpal tunnel syndrome (CTS). CTS is a condition that occurs when the median nerve, which runs through the wrist, becomes compressed or squeezed. This compression can lead to tingling, pain, and numbness in the fingers, particularly at night. Difficulty picking up small objects can also be a symptom of CTS, as it can affect hand dexterity.

However, it's important to note that there could be other conditions or factors contributing to your symptoms. To receive an accurate diagnosis, I highly recommend consulting with a healthcare professional. They will be able to evaluate your symptoms, perform a physical examination, and possibly order additional tests to determine the underlying cause of your symptoms and provide appropriate treatment.

**Q2a**

I'm not a doctor, but I can provide you with some general information. The symptoms you described, including numbness in the hands, difficulty with fine movements, and an unsteady walk, could be indicative of a neurological condition. It's important to consult with a healthcare professional for an accurate diagnosis. However, I can give you a primary diagnosis and a list of five potential differential diagnoses based on the symptoms you mentioned:

Primary Diagnosis:

Peripheral Neuropathy: Peripheral neuropathy refers to damage or dysfunction of the peripheral nerves, leading to symptoms such as numbness, tingling, and difficulty with fine movements. It can affect both the hands and feet and may cause balance problems.

Potential Differential Diagnoses:

Multiple Sclerosis (MS): MS is a chronic autoimmune disease that affects the central nervous system, leading to a variety of symptoms, including numbness, weakness, and coordination difficulties.

Diabetic Neuropathy: Diabetes can cause nerve damage, leading to peripheral neuropathy. This can result in numbness, tingling, and difficulty with fine motor skills.

Guillain-Barré Syndrome: This is a rare autoimmune disorder that affects the peripheral nerves, causing weakness, numbness, and difficulty with coordination and balance.

Cervical Radiculopathy: Compression or irritation of the nerves in the cervical spine (neck region) can lead to symptoms such as numbness in the hands, weakness, and coordination problems.

Parkinson's Disease: Parkinson's disease is a progressive neurological disorder that affects movement. In addition to motor symptoms like an unsteady walk, it can also cause hand tremors and difficulty with fine motor skills.

Please keep in mind that these are just potential differential diagnoses, and a healthcare professional would be best suited to evaluate your symptoms, conduct a thorough examination, and order any necessary tests to reach a definitive diagnosis.

**Q2b**

I'm not a doctor, but based on the symptoms you described, one possible disease that could be causing your symptoms is peripheral neuropathy. Peripheral neuropathy is a condition that involves damage or dysfunction of the peripheral nerves, which can lead to numbness, tingling, and difficulty with fine movements in the affected areas, such as the hands.

Peripheral neuropathy can have various causes, including diabetes, vitamin deficiencies, autoimmune disorders, infections, and exposure to certain toxins. To determine the underlying cause and provide an accurate diagnosis, it's important to consult with a healthcare professional. They will be able to evaluate your symptoms, perform a thorough examination, and may order additional tests such as nerve conduction studies or blood tests to help identify the specific cause of your symptoms.

Remember, this is just one possible diagnosis, and it's essential to consult with a healthcare professional for an accurate evaluation and appropriate treatment.

**Q3b**

I'm not a doctor, but based on the symptoms you described, one possible disease that could be causing your symptoms is lumbar spinal stenosis. Lumbar spinal stenosis is a condition in which the spinal canal in the lower back narrows, putting pressure on the nerves that travel through it. This can lead to symptoms such as lower back pain, numbness or pain in the buttocks and calves, and an increase in symptoms with prolonged walking or standing, which can be relieved by leaning forward.

However, there are other conditions that can cause similar symptoms, so it's important to consult with a healthcare professional for an accurate diagnosis. Here is a list of five potential differential diagnoses:

Primary Diagnosis:

Lumbar Spinal Stenosis: Narrowing of the spinal canal in the lower back, leading to compression of the nerves and resulting in pain, numbness, and symptoms that worsen with activity and improve with forward bending.

Potential Differential Diagnoses:

Herniated Disc: A condition in which the gel-like material within a spinal disc protrudes and presses on nearby nerves, causing pain, numbness, and weakness.

Sciatica: Compression or irritation of the sciatic nerve, which runs from the lower back down the back of the legs, resulting in lower back pain, buttock pain, and leg symptoms.

Degenerative Disc Disease: Wear and tear of the spinal discs over time, leading to pain, stiffness, and possible nerve compression.

Spondylolisthesis: A condition in which one vertebra slips forward or backward onto another, causing lower back pain, nerve compression, and possible leg symptoms.

Piriformis Syndrome: Irritation or compression of the sciatic nerve by the piriformis muscle in the buttock, resulting in buttock pain, leg pain, and possible numbness.

Remember, these are just potential differential diagnoses, and it's important to consult with a healthcare professional for a proper evaluation and accurate diagnosis. They will be able to conduct a thorough examination, review your medical history, and possibly order imaging tests to help determine the underlying cause of your symptoms.

**Q4a**

I'm not a doctor, but based on the symptoms you described, one possible disease that could be causing your symptoms is knee osteoarthritis. Knee osteoarthritis is a degenerative joint disease that involves the breakdown of cartilage in the knee joint, leading to symptoms such as swelling, pain during weight-bearing activities like walking, stiffness, and cracking sounds when moving the knee.

However, there are other conditions that can cause similar symptoms, so it's important to consult with a healthcare professional for an accurate diagnosis. Here is a list of five potential differential diagnoses:

Primary Diagnosis:

Knee Osteoarthritis: Degenerative joint disease that affects the knee, causing symptoms like knee swelling, pain during walking, stiffness, and cracking sounds.

Potential Differential Diagnoses:

Rheumatoid Arthritis: An autoimmune disorder that causes inflammation in the joints, including the knee, leading to swelling, pain, stiffness, and possible joint deformity.

Knee Bursitis: Inflammation of the bursae (fluid-filled sacs) around the knee joint, resulting in swelling, pain, and stiffness.

Meniscus Tear: A tear in the rubbery meniscus cartilage in the knee, which can cause swelling, pain, stiffness, and a sensation of catching or locking of the knee joint.

Ligament Injury (e.g., ACL or MCL Tear): Damage to the ligaments in the knee, often caused by sports or other physical activities, leading to swelling, pain, instability, and difficulty with knee movements.

Gout: A form of inflammatory arthritis caused by the buildup of uric acid crystals in the joints, including the knee, resulting in sudden and severe pain, swelling, redness, and limited mobility.

Please note that these are just potential differential diagnoses, and it's important to consult with a healthcare professional for a proper evaluation and accurate diagnosis. They will be able to conduct a thorough examination, review your medical history, and possibly order imaging tests (such as X-rays or an MRI) to help determine the underlying cause of your symptoms.

**Q4c**

Again, I'm not a doctor, but based on the symptoms you described, one possible disease that could be causing your symptoms is knee osteoarthritis. Knee osteoarthritis is a degenerative joint disease characterized by the breakdown of cartilage in the knee joint. It often leads to symptoms such as knee swelling, pain during weight-bearing activities like walking, stiffness, and cracking or popping sounds when moving the knee.

However, it's important to note that there could be other conditions or factors contributing to your symptoms. To receive an accurate diagnosis, I highly recommend consulting with a healthcare professional. They will be able to evaluate your symptoms, conduct a physical examination, and possibly order imaging tests, such as X-rays or an MRI, to determine the underlying cause of your symptoms.

Remember, this is just one possible diagnosis, and it's essential to consult with a healthcare professional for an accurate evaluation and appropriate treatment.

**Q5a**

I'm not a doctor, but based on the symptoms you described, one possible disease that could be causing your symptoms is hip osteoarthritis. Hip osteoarthritis is a degenerative joint disease that involves the breakdown of cartilage in the hip joint. It often leads to symptoms such as hip pain during weight-bearing activities like walking, stiffness, and cracking or popping sounds when moving the hip.

However, there are other conditions that can cause similar symptoms, so it's important to consult with a healthcare professional for an accurate diagnosis. Here is a list of five potential differential diagnoses:

Primary Diagnosis:

Hip Osteoarthritis: Degenerative joint disease that affects the hip, causing symptoms like hip pain during walking, stiffness, and cracking sounds.

Potential Differential Diagnoses:

Hip Bursitis: Inflammation of the bursae (fluid-filled sacs) around the hip joint, resulting in hip pain, tenderness, and stiffness.

Hip Labral Tear: A tear in the ring of cartilage (labrum) that lines the hip socket, leading to hip pain, clicking or catching sensations, and limited range of motion.

Hip Impingement: Also known as femoroacetabular impingement (FAI), it involves abnormal contact between the ball and socket of the hip joint, causing pain, stiffness, and limited movement.

Hip Tendonitis: Inflammation of the tendons in the hip region, leading to hip pain, stiffness, and difficulty with certain movements.

Avascular Necrosis: A condition in which the blood supply to the hip joint is compromised, leading to bone tissue death and subsequent hip pain, stiffness, and joint dysfunction.

Please note that these are just potential differential diagnoses, and it's important to consult with a healthcare professional for a proper evaluation and accurate diagnosis. They will be able to conduct a thorough examination, review your medical history, and possibly order imaging tests, such as X-rays or an MRI, to help determine the underlying cause of your symptoms.

**Q5c**

I'm not a doctor, but based on the symptoms you described, one possible disease that could be causing your symptoms is hip osteoarthritis. Hip osteoarthritis is a degenerative joint disease that involves the breakdown of cartilage in the hip joint. It can lead to symptoms such as hip swelling, pain during weight-bearing activities like walking, stiffness, and cracking or popping sounds when moving the hip.

However, it's important to note that there could be other conditions or factors contributing to your symptoms. To receive an accurate diagnosis, I highly recommend consulting with a healthcare professional. They will be able to evaluate your symptoms, conduct a physical examination, and possibly order imaging tests, such as X-rays or an MRI, to determine the underlying cause of your symptoms.

Remember, this is just one possible diagnosis, and it's essential to consult with a healthcare professional for an accurate evaluation and appropriate treatment.

**Day 4**

**Q1a**

I'm not a doctor, but I can try to provide you with some information based on the symptoms you've described. Tingling and pain in the fingers, particularly at night, along with difficulty picking up small objects, could be associated with several conditions. It's important to consult with a healthcare professional for a proper diagnosis. However, here are a primary diagnosis and five potential differential diagnoses to consider:

Primary Diagnosis:

Carpal Tunnel Syndrome (CTS): This is a common condition that occurs when the median nerve, which runs from the forearm to the hand, becomes compressed or squeezed at the wrist. It often leads to symptoms such as tingling, numbness, pain, and weakness in the hand and fingers, particularly at night.

Potential Differential Diagnoses:

Peripheral Neuropathy: This refers to damage or dysfunction of the peripheral nerves, which can cause symptoms like tingling, pain, and weakness in the hands and fingers. It can have various causes, including diabetes, vitamin deficiencies, infections, and certain medications.

Cubital Tunnel Syndrome: This condition occurs when the ulnar nerve, which runs along the inner side of the elbow, becomes compressed or irritated. It can result in tingling, pain, and weakness in the ring finger, little finger, and the inner side of the hand.

Thoracic Outlet Syndrome: This condition involves compression or irritation of nerves and blood vessels between the neck and shoulder, which can cause symptoms such as tingling, pain, and weakness in the hand and fingers.

Rheumatoid Arthritis: This is an autoimmune condition that primarily affects the joints but can also cause inflammation and symptoms in other parts of the body, including the hands. Rheumatoid arthritis can lead to pain, swelling, and difficulty with fine motor tasks.

Nerve Entrapment in the Neck: Compression or irritation of nerves in the neck region, such as cervical radiculopathy or cervical spondylosis, can cause radiating pain, tingling, and weakness in the arms, including the fingers.

Remember, these are just potential conditions based on the information provided, and a healthcare professional would be best equipped to evaluate your symptoms, perform a thorough examination, and provide an accurate diagnosis.

**Q1c**

I'm not a doctor, but based on the symptoms you've described, one possible condition that could be causing your symptoms is carpal tunnel syndrome (CTS). Carpal tunnel syndrome is a condition where the median nerve, which runs from your forearm to your hand, becomes compressed or squeezed at the wrist.

Some of the common symptoms of carpal tunnel syndrome include:

Tingling or numbness: You may experience tingling sensations or numbness in your fingers, particularly the thumb, index, middle, and ring fingers.

Pain: You may feel pain or aching in your hand or wrist, which can sometimes extend up to your arm.

Weakness: Carpal tunnel syndrome can cause weakness in your hand, making it difficult to grip or pick up small objects.

Worsening symptoms at night: Many individuals with carpal tunnel syndrome experience increased discomfort and tingling sensations during the night, which can disrupt sleep.

It's important to note that a proper diagnosis can only be provided by a healthcare professional who can evaluate your symptoms, conduct a physical examination, and possibly order additional tests such as nerve conduction studies.

Other conditions could potentially cause similar symptoms, so it's crucial to consult with a medical professional to receive an accurate diagnosis and appropriate treatment.

**Q2a**

I'm not a doctor, but based on the symptoms you've described, one possible primary diagnosis could be peripheral neuropathy. Peripheral neuropathy refers to damage or dysfunction of the peripheral nerves, which are responsible for transmitting signals between the central nervous system (brain and spinal cord) and the rest of the body.

Here are five potential differential diagnoses to consider:

Diabetic Neuropathy: This condition is a type of peripheral neuropathy that is associated with long-term diabetes. It can cause numbness, tingling, and loss of sensation in the hands and feet, as well as difficulties with fine motor movements.

Guillain-Barré Syndrome: This is a rare autoimmune disorder where the immune system mistakenly attacks the peripheral nerves. It often starts with weakness and tingling in the legs and can progress to affect the hands and arms, leading to difficulties with fine movements and an unsteady gait.

Multiple Sclerosis (MS): MS is an autoimmune disease that affects the central nervous system. It can cause a wide range of symptoms, including numbness or tingling in the hands, coordination problems, and difficulties with balance and walking.

Charcot-Marie-Tooth Disease: This is an inherited neurological disorder that affects the peripheral nerves. It can lead to progressive weakness and muscle wasting in the hands and feet, resulting in difficulties with fine movements and an unsteady gait.

Cervical Spondylosis: This condition involves degenerative changes in the cervical spine (neck), which can lead to compression of the nerves. It may cause symptoms such as numbness, weakness, and difficulties with coordination and walking.

It's important to consult with a healthcare professional for a thorough evaluation of your symptoms, a physical examination, and any necessary diagnostic tests to determine the underlying cause and provide an accurate diagnosis.

**Q2b**

I'm not a doctor, but based on the symptoms you've described, one possible condition that could be causing your symptoms is peripheral neuropathy. Peripheral neuropathy refers to damage or dysfunction of the peripheral nerves, which are responsible for transmitting signals between the central nervous system (brain and spinal cord) and the rest of the body.

Some common symptoms of peripheral neuropathy include:

Numbness or tingling: You may experience a loss of sensation or abnormal sensations, such as tingling or pins and needles, in your hands.

Difficulty with fine motor movements: Peripheral neuropathy can affect the coordination and dexterity of your hands, making it challenging to perform precise movements needed for tasks like buttoning a shirt or handling small objects.

Weakness: You may notice weakness or a sense of clumsiness in your hands.

Pain: In some cases, peripheral neuropathy can also cause pain or a burning sensation in the affected areas.

Sensitivity to touch: Some individuals with peripheral neuropathy may experience increased sensitivity to touch or temperature changes.

It's important to note that peripheral neuropathy can have various causes, including diabetes, nutritional deficiencies, certain medications, autoimmune disorders, infections, and more. To receive an accurate diagnosis and appropriate treatment, it's essential to consult with a healthcare professional who can evaluate your symptoms, conduct a physical examination, and possibly order additional tests, such as nerve conduction studies or blood tests.

Keep in mind that this is just one possible explanation for your symptoms, and a healthcare professional would be best suited to provide a definitive diagnosis based on a thorough evaluation.

**Q3b**

I'm not a doctor, but based on the symptoms you've described, one possible primary diagnosis could be lumbar spinal stenosis. Lumbar spinal stenosis refers to a narrowing of the spinal canal in the lower back, which can put pressure on the nerves that travel through it.

Here are five potential differential diagnoses to consider:

Herniated Disc: A herniated disc occurs when the soft center of a spinal disc pushes through a crack in the tougher outer layer, potentially compressing nearby nerves and causing symptoms such as lower back pain and radiating pain, numbness, or tingling in the buttocks and legs.

Sciatica: Sciatica is a condition characterized by irritation or compression of the sciatic nerve, which is the largest nerve in the body. It can cause lower back pain, buttock pain, and numbness, tingling, or pain that radiates down the back of the leg.

Degenerative Disc Disease: Degenerative disc disease refers to the gradual breakdown of the spinal discs, often associated with aging. It can lead to lower back pain, as well as radiating pain, numbness, or tingling in the buttocks and legs.

Spondylolisthesis: Spondylolisthesis occurs when a vertebra slips forward over the vertebra below it, which can narrow the spinal canal and put pressure on the nerves. It may cause lower back pain, buttock pain, and symptoms similar to lumbar spinal stenosis.

Piriformis Syndrome: The piriformis muscle, located in the buttock region, can sometimes irritate or compress the sciatic nerve, leading to symptoms resembling sciatica, including buttock pain and radiating pain, numbness, or tingling in the legs.

Please note that these are just potential conditions based on the information provided, and a healthcare professional would be best equipped to evaluate your symptoms, conduct a thorough examination, and order any necessary tests to provide an accurate diagnosis.

**Q4a**

I'm not a doctor, but based on the symptoms you've described, one possible primary diagnosis could be knee osteoarthritis. Knee osteoarthritis is a degenerative joint disease that involves the gradual wearing down of the cartilage in the knee joint.

Here are five potential differential diagnoses to consider:

Rheumatoid Arthritis: Rheumatoid arthritis is an autoimmune disease that can affect multiple joints, including the knees. It causes inflammation, pain, swelling, and stiffness in the affected joints.

Meniscus Tear: A meniscus tear refers to damage or a tear in the rubbery cartilage (meniscus) that cushions the knee joint. It can cause knee pain, swelling, stiffness, and a feeling of the knee "catching" or "locking" during movement.

Bursitis: Bursitis is the inflammation of the fluid-filled sacs (bursae) that cushion the joints. In the knee, bursitis can cause pain, swelling, and stiffness, particularly over the kneecap area.

Ligament Injury: Injuries to the ligaments around the knee, such as an anterior cruciate ligament (ACL) tear or a medial collateral ligament (MCL) sprain, can cause knee pain, swelling, instability, and difficulty walking.

Gout: Gout is a type of arthritis caused by the buildup of uric acid crystals in the joints. It commonly affects the big toe but can also affect the knee, causing sudden, intense pain, swelling, and redness.

It's important to consult with a healthcare professional for a proper evaluation of your symptoms, a physical examination, and potentially imaging tests such as X-rays or MRI to determine the underlying cause and provide an accurate diagnosis.

**Q4c**

I'm not a doctor, but based on the symptoms you've described, one possible condition that could be causing your symptoms is knee osteoarthritis. Knee osteoarthritis is a degenerative joint disease characterized by the gradual breakdown of the cartilage in the knee joint.

Some common symptoms of knee osteoarthritis include:

Knee pain: You may experience pain in the knee joint, which can be aggravated by activities such as walking, running, or climbing stairs.

Swelling: The knee joint may appear swollen or puffy, especially after periods of activity.

Stiffness: You may feel stiffness in the knee joint, particularly after prolonged periods of rest or inactivity.

Crunching or cracking sounds: Some individuals with knee osteoarthritis report hearing crunching or cracking sounds (crepitus) when bending or extending the knee joint.

Limited range of motion: The flexibility and range of motion in your knee joint may be reduced, making it difficult to fully bend or straighten your leg.

It's important to note that knee osteoarthritis is just one possible explanation for your symptoms. Other conditions, such as rheumatoid arthritis, meniscal tears, ligament injuries, or bursitis, can also cause similar symptoms. A healthcare professional would be best suited to evaluate your symptoms, conduct a physical examination, and possibly order additional tests, such as X-rays or MRI, to provide an accurate diagnosis.

If you are experiencing persistent knee pain and swelling, I recommend consulting with a healthcare professional for a proper evaluation and appropriate treatment options.

**Q5a**

I'm not a doctor, but based on the symptoms you've described, one possible primary diagnosis could be hip osteoarthritis. Hip osteoarthritis is a degenerative joint disease that involves the gradual breakdown of the cartilage in the hip joint.

Here are five potential differential diagnoses to consider:

Bursitis: Hip bursitis is the inflammation of the bursae, which are fluid-filled sacs that cushion the hip joint. It can cause hip pain, particularly on the outer side of the hip, and stiffness that may worsen with movement.

Hip Labral Tear: A hip labral tear refers to a tear in the cartilage (labrum) that lines the hip socket. It can cause hip pain, stiffness, and a clicking or locking sensation during movement.

Femoroacetabular Impingement (FAI): FAI is a condition characterized by abnormal contact between the hip joint's ball and socket. It can cause hip pain, stiffness, and a feeling of catching or locking during certain movements.

Hip Tendonitis: Tendonitis is the inflammation of a tendon. In the hip, tendonitis can cause hip pain, stiffness, and difficulty with activities such as walking or climbing stairs.

Hip Fracture: A hip fracture is a break in the upper part of the thigh bone (femur) near the hip joint. It typically causes severe hip pain, difficulty walking or bearing weight, and significant limitations in hip movement.

It's important to consult with a healthcare professional for a proper evaluation of your symptoms, a physical examination, and potentially imaging tests such as X-rays or MRI to determine the underlying cause and provide an accurate diagnosis.

**Q5c**

I'm not a doctor, but based on the symptoms you've described, one possible condition that could be causing your symptoms is hip osteoarthritis. Hip osteoarthritis is a degenerative joint disease that involves the gradual breakdown of the cartilage in the hip joint.

Some common symptoms of hip osteoarthritis include:

Hip pain: You may experience pain in the hip joint, which can be aggravated by weight-bearing activities such as walking or climbing stairs.

Swelling: The hip joint may appear swollen or inflamed.

Stiffness: You may feel stiffness in the hip joint, especially after periods of rest or inactivity.

Cracking or popping sounds: Some individuals with hip osteoarthritis report hearing cracking or popping sounds (crepitus) when moving their hip joint.

Limited range of motion: The flexibility and range of motion in your hip joint may be reduced, making it difficult to perform certain movements or activities.

It's important to note that hip osteoarthritis is just one possible explanation for your symptoms. Other conditions, such as hip bursitis, tendinitis, hip labral tears, or other inflammatory joint conditions, can also cause similar symptoms. A healthcare professional would be best suited to evaluate your symptoms, conduct a physical examination, and possibly order additional tests, such as X-rays or MRI, to provide an accurate diagnosis.

If you are experiencing persistent hip pain, swelling, or difficulty with mobility, I recommend consulting with a healthcare professional for a proper evaluation and appropriate treatment options.

**Day5**

**Q1a**

I'm not a doctor, but I can provide you with some information. The symptoms you described, including tingling, pain in the fingers (especially at night), and difficulty picking up small objects, can be indicative of various conditions affecting the hand and fingers. It's important to consult a healthcare professional for an accurate diagnosis. That being said, here's a primary diagnosis and a list of five potential differential diagnoses to consider:

Primary Diagnosis:

Carpal Tunnel Syndrome (CTS): This is a common condition caused by compression of the median nerve as it passes through the wrist. It often causes symptoms such as tingling, pain, and numbness in the thumb, index, middle, and part of the ring finger.

Potential Differential Diagnoses:

Peripheral Neuropathy: This refers to damage or dysfunction of peripheral nerves, which can result in symptoms like tingling, pain, and weakness in the hands and fingers. Diabetes, vitamin deficiencies, and autoimmune disorders can cause peripheral neuropathy.

Cubital Tunnel Syndrome: Similar to carpal tunnel syndrome, cubital tunnel syndrome involves compression of the ulnar nerve, which runs along the inside of the elbow. Symptoms include tingling, pain, and weakness in the ring and little fingers.

Rheumatoid Arthritis: This chronic autoimmune condition primarily affects the joints but can also cause inflammation and nerve compression, leading to symptoms like pain, tingling, and difficulty with fine motor tasks.

Thoracic Outlet Syndrome: It occurs due to compression of nerves or blood vessels in the upper chest area, resulting in symptoms like pain, tingling, and weakness in the neck, shoulder, arm, and fingers.

Brachial Plexus Injury: Damage to the brachial plexus, a network of nerves that control the shoulder, arm, and hand, can cause symptoms including tingling, pain, and weakness in the affected area.

Remember, these are just potential differential diagnoses, and a medical professional would be able to evaluate your symptoms, perform any necessary tests or examinations, and provide an accurate diagnosis.

**Q1c**

The symptoms you mentioned, including tingling, pain (especially at night), and difficulty picking up small objects, are commonly associated with a condition called carpal tunnel syndrome (CTS). CTS occurs when the median nerve, which runs from the forearm into the hand, becomes compressed or squeezed at the wrist. This compression can be caused by various factors, such as repetitive hand movements, injury, or underlying medical conditions.

In carpal tunnel syndrome, the symptoms often affect the thumb, index, middle, and part of the ring finger. They may include:

Tingling or numbness: You may experience a pins-and-needles sensation or numbness in the affected fingers.

Pain or discomfort: The tingling may be accompanied by pain or discomfort in the hand or wrist.

Weakness or difficulty gripping: You may have difficulty picking up small objects or performing tasks that require fine motor skills.

It's important to note that while carpal tunnel syndrome is a common cause of these symptoms, a proper diagnosis should be made by a healthcare professional. They will consider your medical history, conduct a physical examination, and may order tests such as nerve conduction studies to confirm the diagnosis and rule out other possible causes.

**Q2a**

I'm not a doctor, but I can provide you with some information based on your symptoms. The combination of numbness in the hands, difficulty with fine movements, and an unsteady walk, particularly when going downstairs, can be indicative of several conditions. It's important to consult a healthcare professional for an accurate diagnosis. Here's a primary diagnosis and a list of five potential differential diagnoses to consider:

Primary Diagnosis:

Peripheral Neuropathy: Peripheral neuropathy refers to damage or dysfunction of peripheral nerves, which can result in various symptoms, including numbness, tingling, and difficulty with fine motor skills. It can affect the hands and feet and is commonly caused by conditions such as diabetes, nutritional deficiencies, autoimmune disorders, infections, or certain medications.

Potential Differential Diagnoses:

Multiple Sclerosis (MS): MS is a chronic autoimmune disease that affects the central nervous system. It can cause symptoms such as numbness or tingling in the hands, difficulty with coordination and fine movements, and gait problems.

Guillain-Barré Syndrome (GBS): GBS is a rare neurological disorder characterized by weakness, numbness, and tingling in the limbs. It can lead to difficulties with fine motor skills and an unsteady gait. GBS often starts in the legs and can progress upwards.

Cervical Radiculopathy: This condition occurs when a nerve root in the neck is compressed or irritated, leading to symptoms like numbness, weakness, and difficulty with fine movements in the hands. It can also cause pain that radiates down the arm.

Vitamin B12 Deficiency: Insufficient levels of vitamin B12 can cause peripheral neuropathy, leading to symptoms similar to those you described. Deficiency in this vitamin can result from malnutrition, certain medications, or malabsorption disorders.

Parkinson's Disease: Although primarily known for its motor symptoms, Parkinson's disease can also cause sensory abnormalities, including numbness and tingling in the hands. It can also lead to difficulties with fine motor control and balance, which might contribute to an unsteady walk.

These are just potential differential diagnoses, and a healthcare professional would be best equipped to evaluate your symptoms, conduct any necessary tests or examinations, and provide an accurate diagnosis.

**Q2b**

I'm not a doctor, but the combination of numbness in the hands and difficulty with fine movements could be indicative of several conditions. One potential condition that could cause these symptoms is peripheral neuropathy. Peripheral neuropathy refers to damage or dysfunction of the peripheral nerves, which can result in numbness, tingling, and weakness in the affected areas, including the hands.

There are various possible causes of peripheral neuropathy, including:

Diabetes: High blood sugar levels can cause nerve damage over time, leading to peripheral neuropathy. It often starts in the feet and can gradually progress to the hands.

Nutritional deficiencies: Deficiencies in certain vitamins, such as vitamin B12, can affect nerve health and contribute to peripheral neuropathy.

Alcohol abuse: Chronic excessive alcohol consumption can lead to nutritional deficiencies and nerve damage, resulting in peripheral neuropathy.

Autoimmune disorders: Conditions like rheumatoid arthritis, lupus, or Guillain-Barré syndrome can cause damage to the peripheral nerves and lead to symptoms of numbness and difficulty with fine motor skills.

Nerve compression: Pressure on the nerves, such as in carpal tunnel syndrome or cervical radiculopathy (nerve compression in the neck), can cause numbness and difficulty with fine movements.

It's important to consult a healthcare professional for a proper evaluation and diagnosis. They will consider your medical history, perform a physical examination, and may order additional tests, such as nerve conduction studies or blood tests, to determine the underlying cause of your symptoms.

**Q3b**

I'm not a doctor, but based on the symptoms you described, the primary diagnosis that comes to mind is a condition called lumbar spinal stenosis. Lumbar spinal stenosis refers to a narrowing of the spinal canal in the lower back, which can put pressure on the nerves and cause symptoms such as lower back pain, numbness, and pain in the buttocks and calves.

Here's a list of five potential differential diagnoses to consider:

Herniated Disc: A herniated disc occurs when the soft cushion-like material between the vertebrae in the spine bulges or ruptures, putting pressure on the nerves. This can cause lower back pain, as well as radiating pain, numbness, and tingling in the buttocks and legs.

Sciatica: Sciatica refers to the compression or irritation of the sciatic nerve, which is the largest nerve in the body. It typically causes pain, numbness, and tingling that radiates from the lower back through the buttocks and down the leg.

Spondylolisthesis: Spondylolisthesis is a condition where one vertebra slips forward or backward in relation to the adjacent vertebrae. This can lead to lower back pain, as well as nerve compression and associated symptoms in the buttocks and legs.

Piriformis Syndrome: The piriformis muscle, located in the buttocks, can sometimes become tight or spasmed, causing compression of the sciatic nerve and resulting in symptoms similar to sciatica.

Degenerative Disc Disease: This refers to the gradual deterioration of the discs in the spine over time, leading to chronic lower back pain and potential nerve compression symptoms in the buttocks and legs.

It's important to consult a healthcare professional for an accurate diagnosis. They will consider your medical history, perform a physical examination, and may order imaging tests such as X-rays or MRI to confirm the underlying cause of your symptoms and recommend appropriate treatment.

**Q4a**

I'm not a doctor, but based on the symptoms you described, the primary diagnosis that comes to mind is knee osteoarthritis. Knee osteoarthritis is a degenerative joint disease that involves the breakdown of the cartilage in the knee joint. It can cause symptoms such as swelling, pain with walking, stiffness, and a cracking or popping sensation when bending the knee.

Here's a list of five potential differential diagnoses to consider:

Rheumatoid Arthritis: Rheumatoid arthritis is an autoimmune condition that causes inflammation in the joints, including the knee. It can result in swelling, pain, stiffness, and joint deformities.

Meniscus Tear: The meniscus is a piece of cartilage in the knee joint that can be torn due to injury or degeneration. A meniscus tear can cause knee pain, swelling, and a feeling of instability.

Patellofemoral Pain Syndrome: This condition involves pain and inflammation around the kneecap (patella). It often occurs due to overuse, muscle imbalances, or abnormal tracking of the patella, leading to knee pain and stiffness.

Bursitis: Bursitis is the inflammation of the bursae, which are fluid-filled sacs that provide cushioning around joints. Knee bursitis can cause swelling, pain, and limited range of motion.

Gout: Gout is a form of arthritis that occurs due to the buildup of uric acid crystals in the joints. It commonly affects the big toe, but the knee can also be involved. Gout can cause sudden, intense pain, swelling, and warmth in the affected joint.

Please note that these are potential differential diagnoses, and a healthcare professional would be best suited to evaluate your symptoms, perform a physical examination, and possibly order imaging tests to provide an accurate diagnosis.

**Q4c**

Based on the symptoms you've described, one possible explanation could be Knee Osteoarthritis. Knee osteoarthritis is a degenerative joint disease characterized by the breakdown of cartilage in the knee joint. It commonly causes swelling, pain, stiffness, and a cracking or grinding sensation when moving the knee.

However, there are several other conditions that could also explain your symptoms, including:

Rheumatoid Arthritis: Rheumatoid arthritis is an autoimmune disease that can affect multiple joints, including the knees. It causes joint inflammation, pain, swelling, and stiffness. Unlike osteoarthritis, rheumatoid arthritis is a systemic condition that may involve other symptoms and affect other joints symmetrically.

Meniscal Tear: A meniscal tear is a common injury that can cause knee pain, swelling, and stiffness. It often occurs due to sudden twisting or a direct impact to the knee, and it may be associated with a feeling of instability or catching.

Bursitis: Knee bursitis refers to the inflammation of the bursae, which are small fluid-filled sacs that cushion the joints. It can cause swelling, pain, and limited mobility in the affected knee.

Gout: Gout is a form of arthritis caused by the buildup of uric acid crystals in the joints. It typically affects the big toe, but it can also involve the knee joint, leading to severe pain, swelling, redness, and warmth.

Ligamentous Injury: An injury to the ligaments of the knee, such as an anterior cruciate ligament (ACL) tear or sprain, can cause pain, swelling, instability, and difficulty walking or bearing weight on the affected knee.

Please keep in mind that these are just potential explanations, and an accurate diagnosis can only be provided by a qualified healthcare professional. It's important to consult with a doctor who can evaluate your symptoms, conduct appropriate tests or imaging, and provide the necessary guidance for your specific situation.

**Q5a**

I'm not a doctor, but based on the symptoms you described, the primary diagnosis that comes to mind is hip osteoarthritis. Hip osteoarthritis is a degenerative joint disease that involves the breakdown of the cartilage in the hip joint. It can cause symptoms such as pain with weight-bearing activities like walking, stiffness, and a cracking or grinding sensation when moving the hip.

Here's a list of five potential differential diagnoses to consider:

Hip Bursitis: Hip bursitis is the inflammation of the bursae, which are fluid-filled sacs that provide cushioning around the hip joint. It can cause pain and tenderness in the hip area, worsened by walking or prolonged sitting.

Hip Labral Tear: The labrum is a ring of cartilage that surrounds the hip socket. A tear in the hip labrum can result from injury or repetitive hip movements. It can cause hip pain, stiffness, and a feeling of catching or locking in the joint.

Tendinitis: Tendinitis refers to inflammation of the tendons around the hip joint. It can cause pain, tenderness, and limited mobility in the hip area.

Hip Impingement: Hip impingement, also known as femoroacetabular impingement (FAI), occurs when there is abnormal contact between the hip joint's ball and socket. This can lead to hip pain, stiffness, and a sensation of catching or clicking during movement.

Avascular Necrosis: Avascular necrosis is a condition in which the blood supply to the hip joint is disrupted, leading to the death of bone tissue. It can cause hip pain, stiffness, and limited range of motion.

These are potential differential diagnoses, and a healthcare professional would be best suited to evaluate your symptoms, perform a physical examination, and possibly order imaging tests (such as X-rays or MRI) to provide an accurate diagnosis.

**Q5c**

I'm not a doctor, but based on the symptoms you described, one potential primary diagnosis could be hip osteoarthritis. Hip osteoarthritis is a degenerative joint disease that involves the breakdown of the cartilage in the hip joint. It can lead to symptoms such as hip pain, swelling, stiffness, and a cracking or popping sensation during movement.

Here's a list of five potential differential diagnoses to consider:

Hip Bursitis: Hip bursitis is the inflammation of the bursae, which are fluid-filled sacs that provide cushioning around the hip joint. It can cause hip pain, swelling, and tenderness, especially with walking or direct pressure on the hip.

Hip Labral Tear: A tear in the labrum, which is the cartilage that lines the hip socket, can lead to symptoms such as hip pain, swelling, stiffness, and a feeling of catching or locking in the joint.

Synovitis: Synovitis refers to inflammation of the synovial lining of the hip joint. It can cause hip pain, swelling, and stiffness, often accompanied by a sensation of warmth in the joint.

Rheumatoid Arthritis: Rheumatoid arthritis is an autoimmune condition that causes inflammation in multiple joints, including the hip. It can result in hip pain, swelling, stiffness, and joint deformity.

Avascular Necrosis: Avascular necrosis occurs when the blood supply to the hip joint is disrupted, leading to the death of bone tissue. It can cause hip pain, swelling, stiffness, and limited range of motion.

It's important to consult a healthcare professional for an accurate diagnosis. They will consider your medical history, perform a physical examination, and may order imaging tests such as X-rays or an MRI to confirm the underlying cause of your symptoms.

**Author, EY**

**Day 1**

**Q1a**

Based on your symptoms, it is possible that you are experiencing carpal tunnel syndrome. This condition is caused by compression of the median nerve as it passes through the carpal tunnel in the wrist. Symptoms often include tingling, numbness, and pain in the fingers (especially at night), as well as weakness and difficulty gripping small objects.

However, it's important to keep in mind that these symptoms can be caused by a variety of conditions, and a proper diagnosis should be made by a medical professional. Here are five potential differential diagnoses to consider:

Cubital tunnel syndrome: This condition is caused by compression of the ulnar nerve at the elbow and can cause tingling, numbness, and pain in the fingers, particularly in the pinky and ring fingers.

Peripheral neuropathy: This is a condition that can be caused by a variety of underlying conditions, including diabetes, and can cause tingling and numbness in the extremities.

Thoracic outlet syndrome: This condition is caused by compression of the nerves and blood vessels that pass between the collarbone and first rib, and can cause tingling, numbness, and pain in the arms and hands.

Rheumatoid arthritis: This is a chronic autoimmune disorder that can cause inflammation and damage to the joints, including the joints in the hands, which can result in pain and difficulty with fine motor tasks.

Multiple sclerosis: This is a neurological disorder that can cause a variety of symptoms, including tingling, numbness, and weakness in the hands and arms.

**Q1c**

Based on the symptoms you have described, it is possible that you are experiencing carpal tunnel syndrome. This condition is caused by pressure on the median nerve as it passes through the carpal tunnel in the wrist. This pressure can cause tingling, numbness, and pain in the fingers, particularly at night, as well as difficulty with fine motor tasks such as picking up small objects.

However, it's important to note that other conditions, such as cubital tunnel syndrome, peripheral neuropathy, or even a nerve injury or compression elsewhere in the arm or neck, could also cause similar symptoms. Therefore, it is recommended to consult with a healthcare professional for an accurate diagnosis and appropriate treatment plan.

**Q2a**

Based on the symptoms you have described, it is possible that you are experiencing peripheral neuropathy. This is a condition that affects the nerves that carry messages to and from the brain and spinal cord, causing numbness and tingling in the hands and feet. The difficulty with fine motor movements and unsteady walk could be related to the sensory and motor deficits caused by peripheral neuropathy.

However, there are other conditions that could also cause similar symptoms. Here are five potential differential diagnoses to consider:

Multiple sclerosis: This is a neurological disorder that affects the central nervous system and can cause a wide range of symptoms, including numbness and tingling in the hands and feet, difficulty with fine motor movements, and problems with balance and coordination.

Diabetic neuropathy: This is a type of peripheral neuropathy that is caused by high blood sugar levels and can cause numbness, tingling, and weakness in the hands and feet.

Cervical spondylosis: This is a condition that affects the neck vertebrae and can compress the spinal cord or nerve roots, causing symptoms such as numbness and weakness in the hands and arms, and difficulty with balance and coordination.

Vitamin B12 deficiency: A deficiency in this vitamin can cause nerve damage and lead to symptoms such as numbness and tingling in the hands and feet, and difficulty with fine motor movements.

Parkinson's disease: This is a neurodegenerative disorder that affects movement, balance, and coordination, and can cause symptoms such as tremors, stiffness, and difficulty with fine motor movements.

**Q2b**

Based on the symptoms you have described, it is possible that you are experiencing carpal tunnel syndrome. This condition is caused by compression of the median nerve as it passes through the carpal tunnel in the wrist. Symptoms often include numbness or tingling in the hands, particularly the thumb, index, middle, and ring fingers, as well as difficulty with fine motor movements, such as handling small objects or buttoning a shirt.

However, other conditions such as peripheral neuropathy, cervical radiculopathy, or cubital tunnel syndrome could also cause similar symptoms. Therefore, it is recommended to consult with a healthcare professional for an accurate diagnosis and appropriate treatment plan.

**Q3b**

Based on the symptoms you have described, it is possible that you are experiencing spinal stenosis. This condition occurs when the spaces within the spine narrow, putting pressure on the spinal cord and nerves. Symptoms can include pain in the lower back, buttocks, and legs, as well as numbness, tingling, and weakness in the legs. Symptoms may worsen with standing or walking and improve with leaning forward or sitting down.

However, there are other conditions that could also cause similar symptoms. Here are five potential differential diagnoses to consider:

Herniated disc: This is a condition in which the gel-like material in a spinal disc pushes out through a tear or rupture, putting pressure on the nerves and causing symptoms such as pain and numbness in the buttocks and legs.

Sciatica: This is a condition that occurs when the sciatic nerve, which runs from the lower back down through the legs, is compressed or irritated. Symptoms can include lower back pain, as well as numbness, tingling, and weakness in the buttocks and legs.

Piriformis syndrome: This is a condition in which the piriformis muscle in the buttocks presses on the sciatic nerve, causing pain, numbness, and tingling in the buttocks and legs.

Osteoarthritis: This is a degenerative joint disease that can affect the spine and cause pain and stiffness, particularly in the lower back.

Spinal cord tumor: This is a rare condition in which a tumor grows within the spinal cord or the membranes that surround it, causing symptoms such as pain, numbness, and weakness in the lower back and legs.

**Q4a**

Based on the symptoms you have described, it is possible that you are experiencing knee osteoarthritis. This is a degenerative joint disease that affects the knee joint, causing symptoms such as pain, swelling, stiffness, and cracking sounds when moving the joint.

However, there are other conditions that could also cause similar symptoms. Here are five potential differential diagnoses to consider:

Rheumatoid arthritis: This is an autoimmune disease that can affect the knee joint, causing pain, swelling, stiffness, and limited range of motion. Unlike osteoarthritis, rheumatoid arthritis is a systemic disease that can affect multiple joints.

Meniscus tear: This is a common injury that occurs when the cartilage in the knee joint is torn, causing pain, swelling, and stiffness. Meniscus tears can be caused by sudden twisting movements or gradual wear and tear.

Knee ligament injury: Injuries to the ligaments in the knee, such as the anterior cruciate ligament (ACL) or medial collateral ligament (MCL), can cause pain, swelling, and stiffness. Ligament injuries can be caused by sudden movements or trauma.

Bursitis: This is a condition in which the bursae, small fluid-filled sacs that cushion the joints, become inflamed. Bursitis can cause pain, swelling, and stiffness in the knee joint.

Gout: This is a type of arthritis that occurs when uric acid crystals build up in the joints, causing pain, swelling, and stiffness. Gout typically affects the big toe, but can also affect the knee joint.

**Q4c**

Based on the symptoms you have described, it is possible that you are experiencing knee osteoarthritis. This is a degenerative joint disease that affects the knee joint, causing symptoms such as pain, swelling, stiffness, and cracking sounds when moving the joint.

However, other conditions could also cause similar symptoms. It is recommended to consult with a healthcare professional for an accurate diagnosis and appropriate treatment plan.

-----------------------------------------------------------------------------

**Q5a**

Based on the symptoms you have described, it is possible that you are experiencing hip osteoarthritis. This is a degenerative joint disease that affects the hip joint, causing symptoms such as pain, stiffness, and cracking sounds when moving the joint. Pain may be particularly noticeable during activities such as walking, standing up from a seated position, or climbing stairs.

However, there are other conditions that could also cause similar symptoms. Here are five potential differential diagnoses to consider:

Bursitis: This is a condition in which the bursae, small fluid-filled sacs that cushion the joints, become inflamed. Bursitis can cause pain and stiffness in the hip joint.

Labral tear: This is a tear in the cartilage that lines the hip socket, which can cause pain, stiffness, and a clicking or catching sensation in the hip joint.

Hip impingement: This is a condition in which the bones of the hip joint rub against each other, causing pain and stiffness. Hip impingement can be caused by structural abnormalities in the hip joint.

Avascular necrosis: This is a condition in which the blood supply to the hip joint is disrupted, causing the bone tissue to die. Avascular necrosis can cause pain, stiffness, and limited range of motion in the hip joint.

Hip fracture: A fracture in the hip joint can cause severe pain, swelling, and difficulty moving the leg. This is a medical emergency that requires prompt attention.

**Q5c**

Based on the symptoms you have described, it is possible that you are experiencing hip osteoarthritis. This is a degenerative joint disease that affects the hip joint, causing symptoms such as pain, stiffness, and cracking sounds when moving the joint. Pain may be particularly noticeable during activities such as walking, standing up from a seated position, or climbing stairs.

However, other conditions could also cause similar symptoms. It is recommended to consult with a healthcare professional for an accurate diagnosis and appropriate treatment plan.

**Day 2**

**Q1a**

Based on the symptoms you described, one possible primary diagnosis could be carpal tunnel syndrome. This is a condition that occurs when the median nerve, which runs from the forearm into the hand, becomes compressed or squeezed at the wrist. This can cause tingling, numbness, and pain in the hand and fingers, especially at night.

However, there are other potential conditions that could be causing your symptoms. Here are five possible differential diagnoses to consider:

Peripheral neuropathy: This condition involves damage to the peripheral nerves, which can cause numbness, tingling, and pain in the hands and feet. It can be caused by a variety of factors, including diabetes, alcoholism, and certain medications.

Cubital tunnel syndrome: This is similar to carpal tunnel syndrome, but affects the ulnar nerve at the elbow instead of the median nerve at the wrist. Symptoms can include tingling, numbness, and pain in the ring and little fingers.

Rheumatoid arthritis: This autoimmune disorder can cause joint pain, swelling, and stiffness, as well as difficulty with fine motor tasks such as picking up small objects.

Raynaud's disease: This condition causes blood vessels in the fingers and toes to narrow, which can cause tingling, numbness, and pain.

Multiple sclerosis: This is a neurological condition that can cause a variety of symptoms, including tingling, numbness, and weakness in the limbs. However, it typically presents with more widespread symptoms beyond the hand and fingers.

**Q1c**

Based on the symptoms you described, it is possible that you have carpal tunnel syndrome. This is a condition that occurs when the median nerve, which runs from the forearm into the hand, becomes compressed or squeezed at the wrist. This can cause tingling, numbness, and pain in the hand and fingers, especially at night. Difficulty picking up small objects is also a common symptom of carpal tunnel syndrome.

It is important to note that other conditions can cause similar symptoms, so it is recommended that you consult with a healthcare provider to receive an accurate diagnosis and appropriate treatment plan.

**Q2a**

Based on the symptoms you described, one possible primary diagnosis could be peripheral neuropathy. This is a condition that involves damage to the peripheral nerves, which can cause numbness, tingling, and weakness in the hands and feet. Peripheral neuropathy can also cause difficulty with fine motor tasks and an unsteady gait, especially when going downstairs.

However, there are other potential conditions that could be causing your symptoms. Here are five possible differential diagnoses to consider:

1. Multiple sclerosis: This is a neurological condition that can cause a variety of symptoms, including numbness and tingling in the hands and feet, difficulty with fine motor tasks, and problems with gait and balance.

2. Diabetic neuropathy: This is a type of peripheral neuropathy that can occur in people with diabetes. It can cause numbness, tingling, and weakness in the hands and feet, as well as difficulty with fine motor tasks.

3. Carpal tunnel syndrome: This is a condition that occurs when the median nerve, which runs from the forearm into the hand, becomes compressed or squeezed at the wrist. It can cause numbness and tingling in the hands, as well as difficulty with fine motor tasks.

4. Parkinson's disease: This is a neurological condition that can cause tremors, rigidity, and difficulty with movement and coordination, including an unsteady gait.

5. Cervical spondylosis: This is a condition that involves degeneration of the spinal discs in the neck. It can cause compression of the nerves in the cervical spine, which can lead to numbness, tingling, and weakness in the hands, as well as difficulty with fine motor tasks and an unsteady gait.

**Q2b**

Based on the symptoms you described, one possible disease is carpal tunnel syndrome. This is a condition that occurs when the median nerve, which runs from the forearm into the hand, becomes compressed or squeezed at the wrist. This can cause numbness, tingling, and weakness in the hand and fingers, especially at night, as well as difficulty with fine motor tasks like buttoning a shirt.

However, other conditions can also cause similar symptoms. For instance, peripheral neuropathy, which is damage to the peripheral nerves, can cause numbness, tingling, and weakness in the hands, and it can also affect fine motor skills. It is therefore recommended that you consult with a healthcare provider for a proper diagnosis and treatment plan.

**Q3b**

Based on the symptoms you described, a possible primary diagnosis could be lumbar spinal stenosis. This is a condition that involves the narrowing of the spinal canal in the lower back, which can put pressure on the nerves that run through the lower back and legs. Symptoms can include lower back pain, numbness, tingling, and weakness in the buttocks and legs, and an improvement in symptoms when leaning forward.

However, there are other potential conditions that could be causing your symptoms. Here are five possible differential diagnoses to consider:

1. Herniated disc: This is a condition that occurs when a disc in the spine slips out of place or ruptures, causing pressure on nearby nerves. Symptoms can include lower back pain, numbness, tingling, and weakness in the buttocks and legs.

2. Sciatica: This is a condition that occurs when the sciatic nerve, which runs from the lower back down the legs, becomes compressed or irritated. Symptoms can include lower back pain, numbness, tingling, and weakness in the buttocks and legs.

3. Piriformis syndrome: This is a condition that occurs when the piriformis muscle in the buttocks becomes tight or inflamed, putting pressure on the sciatic nerve. Symptoms can include lower back pain, numbness, tingling, and weakness in the buttocks and legs.

4. Spinal cord injury: This can occur due to trauma to the spinal cord, causing symptoms such as lower back pain, numbness, tingling, and weakness in the lower extremities.

5. Osteoarthritis: This is a condition that involves the breakdown of cartilage in the joints, including those in the lower back. Symptoms can include lower back pain, stiffness, and difficulty with movement.

**Q4a**

Based on the symptoms you described, one possible primary diagnosis could be knee osteoarthritis. This is a degenerative joint disease that involves the breakdown of cartilage in the knee joint. Symptoms can include knee pain, swelling, stiffness, and a cracking or popping sound when moving the knee.

However, there are other potential conditions that could be causing your symptoms. Here are five possible differential diagnoses to consider:

1. Rheumatoid arthritis: This is an autoimmune disease that can cause joint pain, swelling, stiffness, and deformity. It can affect the knee joint, among other joints in the body.

2. Gout: This is a type of arthritis that occurs when there is a buildup of uric acid in the joints, causing pain, swelling, and stiffness. It commonly affects the big toe joint, but it can also affect the knee.

3. Meniscus tear: The meniscus is a cartilage that cushions and stabilizes the knee joint. A tear in the meniscus can cause knee pain, swelling, and stiffness, as well as a feeling of instability in the knee.

4. ACL injury: The anterior cruciate ligament (ACL) is a ligament in the knee that helps stabilize the joint. An injury to the ACL can cause knee pain, swelling, and instability, as well as a feeling of giving way or buckling in the knee.

5. Bursitis: Bursitis is inflammation of a small sac of fluid (bursa) that cushions and lubricates the knee joint. It can cause knee pain, swelling, and stiffness, as well as difficulty with movement.

Modified Q4c

Based on the symptoms you described, one possible disease is knee osteoarthritis. This is a degenerative joint disease that involves the breakdown of cartilage in the knee joint. Symptoms can include knee pain, swelling, stiffness, and a cracking or popping sound when moving the knee.

However, other conditions can also cause similar symptoms. For instance, a meniscus tear, which is a tear in the cartilage that cushions and stabilizes the knee joint, can cause knee swelling and pain when walking, as well as stiffness and a cracking sound when bending the knee. It is therefore recommended that you consult with a healthcare provider for a proper diagnosis and treatment plan.

**Q5a**

Based on the symptoms you described, one possible primary diagnosis could be hip osteoarthritis. This is a degenerative joint disease that involves the breakdown of cartilage in the hip joint. Symptoms can include hip pain, stiffness, and a cracking or popping sound when moving the hip.

However, there are other potential conditions that could be causing your symptoms. Here are five possible differential diagnoses to consider:

1. Bursitis: This is inflammation of a small sac of fluid (bursa) that cushions and lubricates the hip joint. It can cause hip pain and stiffness, as well as difficulty with movement.

2. Hip labral tear: The labrum is a ring of cartilage that lines the hip joint socket. A tear in the labrum can cause hip pain, stiffness, and a clicking or locking sensation in the hip joint.

3. Hip impingement: This occurs when there is abnormal contact between the bones of the hip joint, leading to pain, stiffness, and a limited range of motion.

4. Avascular necrosis: This is a condition in which the blood supply to the hip joint is disrupted, causing the bone tissue to die. It can cause hip pain, stiffness, and a limited range of motion.

5. Hip fracture: A hip fracture is a break in the thigh bone (femur) that connects to the hip joint. It can cause severe hip pain, stiffness, and difficulty with movement.

**Q5c**

Based on the symptoms you described, one possible disease is hip osteoarthritis. This is a degenerative joint disease that involves the breakdown of cartilage in the hip joint. Symptoms can include hip pain, swelling, stiffness, and a cracking or popping sound when moving the hip.

However, there are other potential conditions that could be causing your symptoms. Here are a few possible differential diagnoses to consider:

1. Bursitis: This is inflammation of a small sac of fluid (bursa) that cushions and lubricates the hip joint. It can cause hip pain, swelling, stiffness, and difficulty with movement.

2. Tendinitis: This is inflammation of a tendon in the hip joint. It can cause hip pain, swelling, and stiffness, particularly when walking or running.

3. Labral tear: The labrum is a ring of cartilage that lines the hip joint socket. A tear in the labrum can cause hip pain, swelling, stiffness, and a clicking or locking sensation in the hip joint.

4. Hip impingement: This occurs when there is abnormal contact between the bones of the hip joint, leading to pain, swelling, stiffness, and a limited range of motion.

5. Septic arthritis: This is a bacterial infection of the hip joint that can cause hip pain, swelling, stiffness, and a fever. It is a serious condition that requires immediate medical attention.

**Day 3**

**Q1a**

Based on the symptoms you have described, the primary diagnosis could be carpal tunnel syndrome. Carpal tunnel syndrome occurs when the median nerve, which runs from the forearm into the hand, becomes compressed at the wrist. This can cause tingling, numbness, and pain in the fingers, as well as weakness in the hand.

Five potential differential diagnoses for these symptoms could include:

1. Peripheral neuropathy - damage to the peripheral nerves, which can cause tingling, numbness, and pain in the hands and feet.

2. Raynaud's disease - a condition where the blood vessels in the fingers constrict, causing pain, numbness, and tingling.

3. Cervical radiculopathy - compression of the nerve roots in the neck, which can cause pain, tingling, and weakness in the hands and arms.

4. Cubital tunnel syndrome - compression of the ulnar nerve at the elbow, which can cause pain, numbness, and tingling in the fingers.

5. Rheumatoid arthritis - a chronic autoimmune disorder that can cause joint pain, stiffness, and swelling in the hands and fingers.

It's important to see a doctor to receive a proper diagnosis and treatment plan.

**Q1c**

Based on the symptoms you have described, it is possible that you have carpal tunnel syndrome. This is a condition where the median nerve, which runs through a narrow passageway in the wrist called the carpal tunnel, becomes compressed or pinched, leading to symptoms such as tingling, numbness, and pain in the fingers, especially at night.

Difficulty picking up small objects can also be a symptom of carpal tunnel syndrome, as the condition can cause weakness in the hand and make it harder to grip or grasp objects. Other possible causes of these symptoms include peripheral neuropathy, cervical radiculopathy, and other nerve or musculoskeletal disorders.

It is important to consult a healthcare provider for an accurate diagnosis and appropriate treatment. They may perform a physical exam and/or diagnostic tests such as nerve conduction studies or electromyography to confirm the diagnosis and determine the severity of the condition. Treatment options may include splinting, physical therapy, medication, or in severe cases, surgery.

**Q2a**

Based on the symptoms you have described, the primary diagnosis could be peripheral neuropathy. Peripheral neuropathy is a condition in which the nerves that carry information to and from the brain and spinal cord to the rest of the body are damaged or diseased. This can cause numbness, tingling, weakness, and other symptoms in the hands and feet, as well as difficulty with fine movements and an unsteady gait.

Five potential differential diagnoses for these symptoms could include:

1. Multiple sclerosis - a chronic autoimmune disorder that affects the central nervous system and can cause numbness, tingling, and difficulty with coordination and balance.

2. Amyotrophic lateral sclerosis (ALS) - a progressive neurodegenerative disease that affects the nerve cells responsible for controlling voluntary muscles, leading to weakness, atrophy, and difficulty with movement and coordination.

3. Parkinson's disease - a neurodegenerative disorder that affects the brain and can cause tremors, stiffness, and difficulty with movement and coordination.

4. Cervical spinal stenosis - a narrowing of the spinal canal in the neck that can compress the spinal cord and nerves, leading to numbness, weakness, and difficulty with coordination and balance.

5. Vitamin B12 deficiency - a condition in which the body does not have enough vitamin B12, which can cause nerve damage and lead to symptoms such as numbness, tingling, and difficulty with movement and balance.

It is important to see a healthcare provider for an accurate diagnosis and appropriate treatment. They may perform a physical exam and/or diagnostic tests such as nerve conduction studies or blood tests to confirm the diagnosis and determine the underlying cause of the symptoms. Treatment options may depend on the specific diagnosis and may include medication, physical therapy, or other therapies to manage the symptoms and improve function.

**Q2b**

Based on the symptoms you have described, the disease you may be experiencing is carpal tunnel syndrome. Carpal tunnel syndrome is a condition in which the median nerve, which runs from the forearm to the palm of the hand, becomes compressed or squeezed as it passes through the wrist. This can cause numbness, tingling, and weakness in the hand and fingers, as well as difficulty with fine movements such as handling small objects or buttoning a shirt.

Other potential causes of these symptoms include peripheral neuropathy, cervical radiculopathy, and other nerve or musculoskeletal disorders. It is important to consult a healthcare provider for an accurate diagnosis and appropriate treatment. They may perform a physical exam and/or diagnostic tests such as nerve conduction studies or electromyography to confirm the diagnosis and determine the severity of the condition. Treatment options may include splinting, physical therapy, medication, or in severe cases, surgery.

**Q3b**

Based on the symptoms you have described, the primary diagnosis could be spinal stenosis. Spinal stenosis is a condition in which the spinal canal narrows and puts pressure on the spinal cord and nerves, leading to pain, numbness, and weakness in the lower back, buttocks, and legs. The symptoms often worsen with standing or walking and may improve when bending forward or sitting.

Five potential differential diagnoses for these symptoms could include:

1. Herniated disc - a condition in which the outer portion of a spinal disc ruptures or bulges, putting pressure on the nearby nerves and causing pain, numbness, and weakness in the lower back and legs.

2. Sciatica - a condition in which the sciatic nerve, which runs from the lower back down to the legs, becomes compressed or irritated, causing pain, numbness, and tingling in the buttocks and legs.

3. Arthritis - a condition in which the joints in the spine become inflamed and may lead to spinal stenosis and nerve compression.

4. Osteoporosis - a condition in which the bones become weak and brittle, leading to fractures in the spine and potentially spinal stenosis.

5. Spondylolisthesis - a condition in which one vertebra slips out of position and puts pressure on the spinal cord or nerves, leading to pain and numbness in the lower back and legs.

It is important to see a healthcare provider for an accurate diagnosis and appropriate treatment. They may perform a physical exam and/or diagnostic tests such as X-rays or MRI scans to confirm the diagnosis and determine the severity of the condition. Treatment options may include physical therapy, medication, injections, or in severe cases, surgery.

**Q4a**

Based on the symptoms you have described, the primary diagnosis could be knee osteoarthritis. Knee osteoarthritis is a degenerative joint disease in which the cartilage in the knee joint wears away, leading to pain, swelling, stiffness, and a cracking or popping sensation when moving the knee.

Five potential differential diagnoses for these symptoms could include:

1. Rheumatoid arthritis - an autoimmune disease in which the immune system attacks the joints, leading to pain, swelling, stiffness, and potential joint damage.

2. Meniscus tear - a tear in the rubbery cartilage that cushions the knee joint, leading to pain, swelling, and limited mobility.

3. Bursitis - inflammation of the small, fluid-filled sacs that cushion the knee joint, leading to pain and swelling.

4. Tendinitis - inflammation of the tendons that attach the muscles to the knee joint, leading to pain, swelling, and difficulty moving the knee.

5. Gout - a type of arthritis caused by a buildup of uric acid crystals in the joint, leading to sudden and severe pain, swelling, and redness.

It is important to see a healthcare provider for an accurate diagnosis and appropriate treatment. They may perform a physical exam and/or diagnostic tests such as X-rays or MRI scans to confirm the diagnosis and determine the severity of the condition. Treatment options may include medication, physical therapy, injections, or in severe cases, surgery.

**Q4c**

Based on the symptoms you have described, the disease you may be experiencing is knee osteoarthritis. Knee osteoarthritis is a degenerative joint disease in which the cartilage in the knee joint wears away, leading to pain, swelling, stiffness, and a cracking or popping sensation when moving the knee.

Other potential causes of these symptoms include knee injuries (such as ligament tears or meniscus tears), knee bursitis, tendinitis, and other forms of arthritis (such as rheumatoid arthritis or gout). It is important to consult a healthcare provider for an accurate diagnosis and appropriate treatment. They may perform a physical exam and/or diagnostic tests such as X-rays or MRI scans to confirm the diagnosis and determine the severity of the condition. Treatment options may include medication, physical therapy, injections, or in severe cases, surgery.

**Q5a**

Based on the symptoms you have described, the primary diagnosis could be hip osteoarthritis. Hip osteoarthritis is a degenerative joint disease in which the cartilage in the hip joint wears away, leading to pain, stiffness, and a cracking or popping sensation when moving the hip.

Five potential differential diagnoses for these symptoms could include:

1. Hip bursitis - inflammation of the small, fluid-filled sacs that cushion the hip joint, leading to pain and swelling.

2. Hip labral tear - a tear in the cartilage that lines the hip socket, leading to pain, stiffness, and limited mobility.

3. Hip tendinitis - inflammation of the tendons that attach the muscles to the hip joint, leading to pain, swelling, and difficulty moving the hip.

4. Avascular necrosis - a condition in which the bone tissue in the hip joint dies due to lack of blood supply, leading to pain and stiffness.

5. Hip fracture - a break in the bones that make up the hip joint, leading to severe pain, swelling, and limited mobility.

It is important to see a healthcare provider for an accurate diagnosis and appropriate treatment. They may perform a physical exam and/or diagnostic tests such as X-rays or MRI scans to confirm the diagnosis and determine the severity of the condition. Treatment options may include medication, physical therapy, injections, or in severe cases, surgery.

Modified Q5c

Based on the symptoms you have described, the disease you may be experiencing is hip osteoarthritis. Hip osteoarthritis is a degenerative joint disease in which the cartilage in the hip joint wears away, leading to pain, stiffness, and a cracking or popping sensation when moving the hip.

Other potential causes of these symptoms include hip bursitis, hip tendinitis, hip labral tear, avascular necrosis, and hip fracture. It is important to consult a healthcare provider for an accurate diagnosis and appropriate treatment. They may perform a physical exam and/or diagnostic tests such as X-rays or MRI scans to confirm the diagnosis and determine the severity of the condition. Treatment options may include medication, physical therapy, injections, or in severe cases, surgery.

**Day 4**

**Q1a**

Based on your symptoms of tingling and pain in your fingers, especially at night, along with difficulty picking up small objects, the primary diagnosis could be carpal tunnel syndrome. This is a common condition that occurs when the median nerve, which runs from the forearm to the hand, is compressed or squeezed as it travels through the wrist.

Here are five potential differential diagnoses:

1. Cubital tunnel syndrome: This is another condition where a nerve, the ulnar nerve, is compressed or pinched, leading to tingling, numbness, or pain in the hand, especially in the ring and little fingers.

2. Peripheral neuropathy: This is a disorder that affects the peripheral nerves, leading to pain, tingling, numbness, or weakness in the hands and feet. It can have various causes, such as diabetes, alcohol abuse, infections, or autoimmune disorders.

3. Raynaud's disease: This is a disorder that affects the blood vessels in the fingers, causing them to narrow and restrict blood flow. It can lead to pain, tingling, and numbness, especially in response to cold or stress.

4. Rheumatoid arthritis: This is an autoimmune disorder that can affect the joints in the hands and fingers, leading to pain, stiffness, and difficulty with fine movements such as gripping or grasping.

5. Dupuytren's contracture: This is a condition where the connective tissue in the palm of the hand thickens and tightens, causing the fingers to curl inward and limiting their movement. It can lead to difficulty with fine motor tasks, such as picking up small objects or buttoning clothes.

Modified Q1c

Based on your symptoms of tingling and pain in your fingers, especially at night, along with difficulty picking up small objects, the most likely diagnosis is carpal tunnel syndrome. This is a common condition that occurs when the median nerve, which runs from the forearm to the hand, is compressed or squeezed as it travels through the wrist.

Other possible causes of your symptoms could include cubital tunnel syndrome, which is a condition where a nerve in the elbow is compressed, or peripheral neuropathy, which is a disorder that affects the peripheral nerves and can cause tingling, numbness, or pain in the hands and feet. However, given the specific symptoms you described, carpal tunnel syndrome is the most likely diagnosis. It is recommended that you see a healthcare professional for an accurate diagnosis and appropriate treatment.

**Q2a**

Based on your symptoms of numbness in your hands, difficulty with fine movements, and an unsteady walk, especially when going downstairs, the primary diagnosis could be multiple sclerosis (MS). MS is a chronic autoimmune disorder that affects the central nervous system, including the brain, spinal cord, and optic nerves. It can cause a wide range of symptoms, including numbness or tingling in the hands and feet, difficulty with coordination, and problems with fine motor skills.

Here are five potential differential diagnoses:

1. Carpal tunnel syndrome: This is a common condition that occurs when the median nerve is compressed as it travels through the wrist. It can cause numbness, tingling, and weakness in the hand and can affect fine motor skills.

2. Peripheral neuropathy: This is a condition that affects the peripheral nerves and can cause numbness, tingling, or pain in the hands and feet. It can be caused by a range of conditions, such as diabetes, alcoholism, or exposure to toxins.

3. Parkinson's disease: This is a progressive neurological disorder that can cause tremors, stiffness, and difficulty with coordination and fine motor skills.

4. Guillain-Barre syndrome: This is a rare disorder that affects the nerves outside the brain and spinal cord and can cause weakness, numbness, or tingling in the hands and feet, as well as difficulty with coordination and walking.

5. Stroke: This is a medical emergency that occurs when blood flow to the brain is interrupted. It can cause a range of symptoms, including numbness, weakness, or loss of coordination in the hands or legs.

**Q2b**

Based on your symptoms of numbness in your hands and difficulty with fine movements, such as buttoning a shirt, the most likely diagnosis is carpal tunnel syndrome. This is a common condition that occurs when the median nerve, which runs from the forearm to the hand, is compressed or squeezed as it travels through the wrist.

Other possible causes of your symptoms could include peripheral neuropathy, which is a disorder that affects the peripheral nerves and can cause numbness, tingling, or pain in the hands and feet, or a cervical spine (neck) injury, which can cause nerve compression leading to hand numbness and difficulty with fine movements.

However, given the specific symptoms you described, carpal tunnel syndrome is the most likely diagnosis. It is recommended that you see a healthcare professional for an accurate diagnosis and appropriate treatment.

**Q3b**

Based on your symptoms of lower back pain, numbness and pain in your buttocks and calves that increases with walking but improves when leaning slightly forward, the primary diagnosis could be lumbar spinal stenosis. This is a condition where the spinal canal in the lower back narrows, putting pressure on the spinal cord and nerves, leading to symptoms like pain, numbness, and weakness in the lower back, buttocks, and legs.

Here are five potential differential diagnoses:

1. Herniated disc: A herniated disc is a condition where the inner portion of a spinal disc protrudes through the outer layer and presses on nearby nerves, causing pain, numbness, or weakness in the affected area.

2. Sciatica: Sciatica is a condition where the sciatic nerve, which runs from the lower back down to the legs, becomes compressed or irritated, causing pain, numbness, or tingling in the buttocks and legs.

3. Osteoarthritis: Osteoarthritis is a condition where the cartilage in the joints breaks down over time, causing pain and stiffness in the affected area. It can also cause numbness and tingling if it affects nearby nerves.

4. Spondylolisthesis: Spondylolisthesis is a condition where a vertebra in the spine slips out of place and presses on nearby nerves, causing pain, numbness, or weakness in the affected area.

5. Piriformis syndrome: Piriformis syndrome is a condition where the piriformis muscle in the buttocks presses on the sciatic nerve, causing pain, numbness, or tingling in the buttocks and legs.

**Q4a**

Based on your symptoms of knee swelling, pain while walking, stiffness, and cracking sounds while bending, the primary diagnosis could be osteoarthritis of the knee. This is a common degenerative joint disease that occurs when the protective cartilage that cushions the ends of the bones in the knee joint wears down over time, causing pain, swelling, and stiffness.

Here are five potential differential diagnoses:

1. Rheumatoid arthritis: Rheumatoid arthritis is an autoimmune disorder that causes inflammation and damage to the joints, including the knee joint. It can cause pain, swelling, stiffness, and cracking sounds while moving the joint.

2. Gout: Gout is a type of arthritis caused by the buildup of uric acid crystals in the joints, including the knee joint. It can cause sudden, severe pain, swelling, and redness in the affected joint.

3. Meniscus tear: A meniscus tear is a common injury that can cause knee pain, swelling, stiffness, and a popping or clicking sensation when bending or straightening the knee.

4. Bursitis: Bursitis is an inflammation of the small fluid-filled sacs (bursae) that cushion the bones, tendons, and muscles near the joint. It can cause knee pain, swelling, and stiffness, especially when bending the knee.

5. Patellofemoral pain syndrome: Patellofemoral pain syndrome is a condition that causes pain in the front of the knee, especially when bending, squatting, or going up and down stairs. It is caused by irritation of the soft tissues around the knee cap (patella).

**Q4c**

Based on your symptoms of knee swelling, pain while walking, stiffness, and cracking sounds while bending, the most likely diagnosis is osteoarthritis of the knee. Osteoarthritis is a common degenerative joint disease that occurs when the protective cartilage that cushions the ends of the bones in the knee joint wears down over time, causing pain, swelling, and stiffness.

However, other conditions can cause similar symptoms, and it's essential to get an accurate diagnosis from a healthcare professional. Here are some potential differential diagnoses:

1. Rheumatoid arthritis: Rheumatoid arthritis is an autoimmune disorder that causes inflammation and damage to the joints, including the knee joint. It can cause pain, swelling, stiffness, and cracking sounds while moving the joint.

2. Gout: Gout is a type of arthritis caused by the buildup of uric acid crystals in the joints, including the knee joint. It can cause sudden, severe pain, swelling, and redness in the affected joint.

3. Meniscus tear: A meniscus tear is a common injury that can cause knee pain, swelling, stiffness, and a popping or clicking sensation when bending or straightening the knee.

4. Bursitis: Bursitis is an inflammation of the small fluid-filled sacs (bursae) that cushion the bones, tendons, and muscles near the joint. It can cause knee pain, swelling, and stiffness, especially when bending the knee.

5. Patellofemoral pain syndrome: Patellofemoral pain syndrome is a condition that causes pain in the front of the knee, especially when bending, squatting, or going up and down stairs. It is caused by irritation of the soft tissues around the kneecap (patella).

**Q5a**

Based on your symptoms of hip pain while walking, stiffness, and cracking sounds when moving, the primary diagnosis could be osteoarthritis of the hip. Osteoarthritis is a common degenerative joint disease that occurs when the protective cartilage that cushions the ends of the bones in the hip joint wears down over time, causing pain, swelling, and stiffness.

Here are five potential differential diagnoses:

1. Rheumatoid arthritis: Rheumatoid arthritis is an autoimmune disorder that causes inflammation and damage to the joints, including the hip joint. It can cause pain, swelling, stiffness, and cracking sounds while moving the joint.

2. Bursitis: Bursitis is an inflammation of the small fluid-filled sacs (bursae) that cushion the bones, tendons, and muscles near the hip joint. It can cause pain, swelling, and stiffness, especially when moving the hip.

3. Hip labral tear: A hip labral tear is an injury to the cartilage lining the hip joint. It can cause pain, stiffness, and clicking or catching sensations when moving the hip.

4. Avascular necrosis: Avascular necrosis is a condition that occurs when there is a loss of blood flow to the hip joint, causing the bone tissue to die. It can cause hip pain, stiffness, and limited range of motion.

5. Hip impingement: Hip impingement is a condition where the bones in the hip joint rub against each other, causing pain, stiffness, and limited range of motion. It is also known as femoroacetabular impingement (FAI).

**Q5c**

Based on your symptoms of hip swelling, pain while walking, stiffness, and cracking sounds when moving, the most likely diagnosis is hip osteoarthritis. Osteoarthritis is a common degenerative joint disease that occurs when the protective cartilage that cushions the ends of the bones in the hip joint wears down over time, causing pain, swelling, and stiffness.

However, other conditions can cause similar symptoms, and it's essential to get an accurate diagnosis from a healthcare professional. Here are some potential differential diagnoses:

1. Rheumatoid arthritis: Rheumatoid arthritis is an autoimmune disorder that causes inflammation and damage to the joints, including the hip joint. It can cause pain, swelling, stiffness, and cracking sounds while moving the joint.

2. Bursitis: Bursitis is an inflammation of the small fluid-filled sacs (bursae) that cushion the bones, tendons, and muscles near the hip joint. It can cause hip pain, swelling, and stiffness, especially when moving the hip.

3. Avascular necrosis: Avascular necrosis is a condition that occurs when there is a loss of blood flow to the hip joint, causing the bone tissue to die. It can cause hip pain, stiffness, and limited range of motion.

4. Hip labral tear: A hip labral tear is an injury to the cartilage lining the hip joint. It can cause hip pain, stiffness, and clicking or catching sensations when moving the hip.

5. Hip impingement: Hip impingement is a condition where the bones in the hip joint rub against each other, causing pain, stiffness, and limited range of motion. It is also known as femoroacetabular impingement (FAI).

**Day 5**

**Q1a**

Based on your symptoms, one possible primary diagnosis is carpal tunnel syndrome (CTS). This is a condition in which the median nerve, which runs through the wrist, becomes compressed, causing tingling, numbness, and pain in the fingers, especially at night.

Five potential differential diagnoses are:

1. Peripheral neuropathy: This is a condition that affects the nerves outside of the brain and spinal cord, causing tingling, numbness, and pain, usually in the hands and feet.

2. Raynaud's disease: This is a condition in which the blood vessels in the fingers and toes narrow, causing numbness, tingling, and pain, especially in response to cold temperatures or stress.

3. Arthritis: This is a group of conditions that cause inflammation in the joints, which can lead to pain, stiffness, and difficulty with movement. Arthritis can affect the hands and fingers, causing difficulty picking up small objects.

4. Thoracic outlet syndrome: This is a condition in which the nerves and blood vessels that pass through the thoracic outlet (an area between the neck and the shoulder) become compressed, causing pain, numbness, and tingling in the arm and hand.

5. Multiple sclerosis: This is a neurological condition that can cause a wide range of symptoms, including tingling, numbness, and weakness in the hands and fingers.

**Q1c**

Based on your symptoms, it is possible that you may be experiencing carpal tunnel syndrome. This condition occurs when the median nerve, which runs through a narrow passage in the wrist called the carpal tunnel, becomes compressed or pinched. This can cause tingling, pain, and numbness in the fingers, particularly the thumb, index, and middle fingers, as well as difficulty gripping or picking up small objects.

However, it is important to note that other conditions, such as peripheral neuropathy, rheumatoid arthritis, or even a pinched nerve in the neck or shoulder, can cause similar symptoms. It is recommended that you see a doctor for a proper diagnosis and treatment plan.

**Q2a**

Based on your symptoms, a possible primary diagnosis is multiple sclerosis (MS). MS is a chronic autoimmune disease that affects the central nervous system, causing damage to the protective sheath that covers nerve fibers. This can result in a wide range of symptoms, including numbness or tingling in the hands, difficulty with fine motor movements, and problems with coordination and balance.

Five potential differential diagnoses are:

1. Carpal tunnel syndrome: This is a condition in which the median nerve, which runs through the wrist, becomes compressed, causing numbness, tingling, and weakness in the hands.

2. Diabetic neuropathy: This is a condition that can develop in people with diabetes, causing damage to the nerves in the hands and feet, resulting in numbness, tingling, and weakness.

3. Parkinson's disease: This is a progressive neurological disorder that affects movement, causing tremors, stiffness, and difficulty with balance and coordination.

4. Cervical radiculopathy: This is a condition in which a nerve root in the neck becomes compressed or inflamed, causing symptoms such as pain, numbness, and weakness in the hands.

5. Vitamin B12 deficiency: This is a condition in which the body does not have enough vitamin B12, which is important for nerve function. Symptoms can include numbness, tingling, and weakness in the hands, as well as problems with balance and coordination.

**Q2b**

Based on your symptoms, a possible disease you may be experiencing is carpal tunnel syndrome (CTS). CTS occurs when the median nerve, which runs through a narrow passage in the wrist called the carpal tunnel, becomes compressed or pinched. This can cause numbness, tingling, or weakness in the hand, particularly in the thumb, index, and middle fingers, as well as difficulty with fine motor movements such as handling small objects or buttoning a shirt.

However, it is important to note that other conditions, such as peripheral neuropathy, arthritis, or a pinched nerve in the neck or shoulder, can also cause similar symptoms. It is recommended that you see a doctor for a proper diagnosis and treatment plan.

**Q3b**

Based on your symptoms, a possible primary diagnosis is lumbar spinal stenosis. This condition occurs when the spinal canal in the lower back narrows, putting pressure on the spinal cord and nerves. This can cause pain in the lower back, as well as numbness, tingling, or weakness in the buttocks, legs, and feet. The symptoms may worsen when standing or walking but improve when bending forward.

Five potential differential diagnoses are:

1. Sciatica: This is a condition that occurs when the sciatic nerve, which runs from the lower back down to the legs, becomes compressed or irritated, causing pain, numbness, or tingling in the buttocks, legs, and feet.

2. Herniated disc: This is a condition in which the soft material inside a spinal disc pushes out through a tear in the disc's outer covering, causing pressure on the nerves in the lower back and legs.

3. Osteoarthritis: This is a degenerative joint disease that can affect the spine, causing pain, stiffness, and reduced mobility.

4. Spondylolisthesis: This is a condition in which one vertebra in the spine slips forward over the one below it, causing pressure on the nerves and leading to pain, numbness, or weakness in the lower back and legs.

5. Piriformis syndrome: This is a condition in which the piriformis muscle in the buttocks compresses the sciatic nerve, causing pain, numbness, or tingling in the buttocks and legs.

**Q4a**

Based on your symptoms, a possible primary diagnosis is knee osteoarthritis. This is a degenerative joint disease that occurs when the cartilage in the knee joint wears down over time, leading to pain, stiffness, and swelling.

Five potential differential diagnoses are:

1. Knee ligament injury: This is an injury to one of the ligaments in the knee, such as the anterior cruciate ligament (ACL) or the medial collateral ligament (MCL). Symptoms may include pain, swelling, and instability.

2. Meniscus tear: This is an injury to the cartilage in the knee, which can occur due to a sudden twisting or bending motion. Symptoms may include pain, swelling, stiffness, and a clicking or popping sensation.

3. Bursitis: This is inflammation of a bursa, a small fluid-filled sac that cushions the knee joint. Symptoms may include pain, swelling, and stiffness, particularly when bending or extending the knee.

4. Gout: This is a type of arthritis caused by the buildup of uric acid crystals in the joint, which can cause sudden and severe pain, redness, and swelling in the knee.

5. Rheumatoid arthritis: This is an autoimmune disease that can affect the joints, causing pain, swelling, and stiffness. In the knee, rheumatoid arthritis can cause inflammation of the synovial membrane, which lines the joint, leading to joint damage and deformity.

**Q4c**

Based on your symptoms, a possible disease you may be experiencing is knee osteoarthritis. This is a degenerative joint disease that occurs when the cartilage in the knee joint wears down over time, leading to pain, stiffness, and swelling.

However, other conditions, such as knee ligament injury, meniscus tear, bursitis, gout, or rheumatoid arthritis, can also cause similar symptoms. It is recommended that you see a doctor for a proper diagnosis and treatment plan.

**Q5a**

Based on your symptoms, a possible primary diagnosis is hip osteoarthritis. This is a degenerative joint disease that occurs when the cartilage in the hip joint wears down over time, leading to pain, stiffness, and cracking sounds during movement.

Five potential differential diagnoses are:

1. Bursitis: This is inflammation of the bursa, a small fluid-filled sac that cushions the hip joint. Symptoms may include pain and tenderness on the outside of the hip, particularly when lying on the affected side.

2. Tendinitis: This is inflammation of the tendons that attach muscles to bones in the hip joint. Symptoms may include pain and tenderness in the front of the hip or groin area, particularly when walking or climbing stairs.

3. Labral tear: This is an injury to the ring of cartilage (labrum) that surrounds the hip joint. Symptoms may include pain in the hip or groin area, stiffness, and a clicking or locking sensation.

4. Avascular necrosis: This is a condition that occurs when the blood supply to the hip joint is interrupted, causing the bone to die and leading to pain and stiffness. It is often associated with risk factors such as steroid use, alcoholism, or a history of hip fractures.

5. Hip impingement: This is a condition that occurs when there is abnormal contact between the bones in the hip joint, leading to pain and stiffness, particularly during activities that involve bending or twisting the hip.

**Q5c**

Based on your symptoms, a possible disease you may be experiencing is hip osteoarthritis. This is a degenerative joint disease that occurs when the cartilage in the hip joint wears down over time, leading to pain, stiffness, and cracking sounds during movement.

However, other conditions such as bursitis, tendinitis, labral tear, avascular necrosis, or hip impingement, can also cause similar symptoms. It is recommended that you see a doctor for a proper diagnosis and treatment plan.

**Author, AY**

**Day 1**

**Q1a**

Based on the symptoms you described, it is possible that you have carpal tunnel syndrome. This is a condition that occurs when the median nerve, which runs from the forearm to the hand, becomes compressed at the wrist. Common symptoms include tingling, numbness, and pain in the hand, particularly in the thumb, index, and middle fingers. The symptoms are often worse at night and can make it difficult to grip small objects.

Here are five potential differential diagnoses:

1. Cubital tunnel syndrome: This condition occurs when the ulnar nerve becomes compressed at the elbow, causing similar symptoms to carpal tunnel syndrome, but affecting different fingers (usually the ring and little finger).

2. Peripheral neuropathy: This is a disorder of the peripheral nerves that can cause numbness, tingling, and pain in various parts of the body, including the hands and fingers.

3. Thoracic outlet syndrome: This is a group of disorders that occur when there is compression of nerves and blood vessels that pass through the narrow space between the collarbone and the first rib. Symptoms can include pain, tingling, and weakness in the arm, hand, and fingers.

4. Rheumatoid arthritis: This is an autoimmune disorder that can cause joint pain, stiffness, and swelling in the hands and other parts of the body.

5. Multiple sclerosis: This is a neurological disorder that can cause a wide range of symptoms, including numbness, tingling, and weakness in various parts of the body, including the hands and fingers.

Please note that this is not a definitive diagnosis, and only a qualified healthcare professional can provide an accurate diagnosis. If you are experiencing symptoms, it is important to consult with a healthcare professional as soon as possible.

**Q1c**

Based on your symptoms, it is possible that you have carpal tunnel syndrome. This is a condition that occurs when the median nerve, which runs from the forearm to the hand, becomes compressed at the wrist. Common symptoms include tingling, numbness, and pain in the hand, particularly in the thumb, index, and middle fingers. The symptoms are often worse at night and can make it difficult to grip small objects.

However, please note that this is not a definitive diagnosis, and only a qualified healthcare professional can provide an accurate diagnosis. If you are experiencing symptoms, it is important to consult with a healthcare professional as soon as possible. They can perform a physical examination, review your medical history, and conduct any necessary tests to determine the underlying cause of your symptoms and develop an appropriate treatment plan.

**Q2a**

Based on your symptoms, it is possible that you have peripheral neuropathy. This is a condition that occurs when the peripheral nerves that transmit signals between the central nervous system and the rest of the body are damaged. Common symptoms include numbness, tingling, and weakness in the hands and feet, as well as difficulty with fine motor movements.

Here are five potential differential diagnoses:

1. Multiple sclerosis: This is a neurological disorder that can cause a wide range of symptoms, including numbness, tingling, and weakness in various parts of the body, including the hands and feet.

2. Diabetic neuropathy: This is a type of peripheral neuropathy that can occur as a complication of diabetes. It is caused by damage to the nerves that control sensation and movement in the feet and legs.

3. Guillain-Barre syndrome: This is a rare disorder that occurs when the body's immune system attacks the peripheral nerves, causing numbness, tingling, and weakness in the hands and feet.

4. Charcot-Marie-Tooth disease: This is a genetic disorder that affects the peripheral nerves, causing weakness and numbness in the hands and feet, as well as difficulty with fine motor movements.

5. Cervical spondylosis: This is a degenerative condition that affects the cervical spine, causing compression of the nerves that run through the neck and into the hands and arms. Symptoms can include numbness, tingling, and weakness in the hands and arms, as well as difficulty with fine motor movements.

Please note that this is not a definitive diagnosis, and only a qualified healthcare professional can provide an accurate diagnosis. If you are experiencing symptoms, it is important to consult with a healthcare professional as soon as possible. They can perform a physical examination, review your medical history, and conduct any necessary tests to determine the underlying cause of your symptoms and develop an appropriate treatment plan.

**Q2b**

Based on your symptoms, it is possible that you have carpal tunnel syndrome. This is a condition that occurs when the median nerve, which runs from the forearm to the hand, becomes compressed at the wrist. Common symptoms include numbness, tingling, and weakness in the hand, particularly in the thumb, index, and middle fingers, as well as difficulty with fine motor movements such as buttoning a shirt.

However, please note that this is not a definitive diagnosis, and only a qualified healthcare professional can provide an accurate diagnosis. If you are experiencing symptoms, it is important to consult with a healthcare professional as soon as possible. They can perform a physical examination, review your medical history, and conduct any necessary tests to determine the underlying cause of your symptoms and develop an appropriate treatment plan.

**Q3b**

Based on your symptoms, it is possible that you have sciatica. Sciatica is a common condition that occurs when the sciatic nerve, which runs from the lower back down to the feet, becomes compressed or irritated. Common symptoms include pain in the lower back, buttocks, and legs, as well as numbness, tingling, and weakness in the legs. The pain may increase with prolonged standing or walking and can often be relieved by leaning forward or lying down.

Here are five potential differential diagnoses:

1. Herniated disc: This is a condition in which the soft, gel-like material inside a spinal disc protrudes through a tear in the disc's outer layer, putting pressure on nearby nerves and causing pain and other symptoms.

2. Spinal stenosis: This is a narrowing of the spinal canal that can put pressure on the nerves that run through it, causing pain, numbness, and weakness in the legs.

3. Spondylolisthesis: This is a condition in which one vertebra in the spine slips forward over the vertebra below it, putting pressure on the nerves that run through the affected area and causing pain, numbness, and weakness in the legs.

4. Piriformis syndrome: This is a condition in which the piriformis muscle, which runs from the base of the spine to the thigh bone, becomes tight or spasms, putting pressure on the sciatic nerve and causing pain, numbness, and tingling in the buttocks and legs.

5. Ankylosing spondylitis: This is a type of arthritis that primarily affects the spine, causing inflammation and stiffness that can lead to pain and other symptoms.

Please note that this is not a definitive diagnosis, and only a qualified healthcare professional can provide an accurate diagnosis. If you are experiencing symptoms, it is important to consult with a healthcare professional as soon as possible. They can perform a physical examination, review your medical history, and conduct any necessary tests to determine the underlying cause of your symptoms and develop an appropriate treatment plan.

**Q4a**

Based on your symptoms, it is possible that you have knee osteoarthritis. This is a degenerative joint disease that occurs when the protective cartilage on the ends of bones wears down over time, leading to pain, swelling, and stiffness in the joint. Cracking or popping sounds when bending the knee can also be a symptom of knee osteoarthritis.

Here are five potential differential diagnoses:

1. Rheumatoid arthritis: This is an autoimmune disorder that can affect the joints, causing pain, swelling, and stiffness. Unlike osteoarthritis, rheumatoid arthritis can affect multiple joints and is often associated with systemic symptoms such as fatigue and fever.

2. Meniscus tear: The meniscus is a piece of cartilage in the knee joint that can tear due to injury or wear and tear. Symptoms include pain, swelling, and stiffness in the knee, as well as difficulty straightening or bending the knee.

3. Gout: This is a type of arthritis caused by a buildup of uric acid crystals in the joints. Symptoms include sudden and severe pain, redness, and swelling in the affected joint, which may be accompanied by fever and chills.

4. Bursitis: This is a condition that occurs when the small fluid-filled sacs (bursae) that cushion the joints become inflamed. Symptoms include pain, swelling, and tenderness in the affected joint.

5. Tendinitis: This is a condition that occurs when a tendon becomes inflamed, usually due to overuse or injury. Symptoms include pain, swelling, and tenderness in the affected area, which may be accompanied by stiffness and difficulty moving the joint.

Please note that this is not a definitive diagnosis, and only a qualified healthcare professional can provide an accurate diagnosis. If you are experiencing symptoms, it is important to consult with a healthcare professional as soon as possible. They can perform a physical examination, review your medical history, and conduct any necessary tests to determine the underlying cause of your symptoms and develop an appropriate treatment plan.

**Q4c**

Based on your symptoms, it is possible that you have knee osteoarthritis. Knee osteoarthritis is a degenerative joint disease that occurs when the protective cartilage on the ends of bones wears down over time, leading to pain, swelling, and stiffness in the joint. Cracking or popping sounds when bending the knee can also be a symptom of knee osteoarthritis.

However, other conditions can cause similar symptoms, such as:

1. Meniscal tear: This occurs when there is a tear in the meniscus, which is a piece of cartilage in the knee joint. This can cause pain, swelling, and stiffness in the knee, along with a clicking or locking sensation.

2. Rheumatoid arthritis: This is an autoimmune disorder that can cause joint pain, swelling, and stiffness. Unlike osteoarthritis, rheumatoid arthritis can affect multiple joints and is often associated with systemic symptoms such as fatigue and fever.

3. Tendinitis: This occurs when there is inflammation or irritation of a tendon, usually due to overuse or injury. Symptoms include pain, swelling, and stiffness in the affected area.

4. Bursitis: This occurs when there is inflammation or irritation of the bursae, which are small sacs of fluid that cushion the joints. Symptoms include pain, swelling, and stiffness in the affected area.

5. Osteonecrosis: This is a condition in which there is a loss of blood supply to the bone, leading to bone death. It can cause pain, swelling, and stiffness in the affected joint, along with a limited range of motion.

Please note that this is not a definitive diagnosis, and only a qualified healthcare professional can provide an accurate diagnosis. If you are experiencing symptoms, it is important to consult with a healthcare professional as soon as possible. They can perform a physical examination, review your medical history, and conduct any necessary tests to determine the underlying cause of your symptoms and develop an appropriate treatment plan.

**Q5a**

Based on your symptoms, it is possible that you have hip osteoarthritis. Hip osteoarthritis is a degenerative joint disease that occurs when the protective cartilage on the ends of bones wears down over time, leading to pain, stiffness, and a limited range of motion in the joint. Cracking or popping sounds when moving the hip can also be a symptom of hip osteoarthritis.

However, other conditions can cause similar symptoms, such as:

1. Bursitis: This occurs when there is inflammation or irritation of the bursae, which are small sacs of fluid that cushion the joints. Symptoms include pain, stiffness, and tenderness in the hip, along with difficulty walking or climbing stairs.

2. Tendinitis: This occurs when there is inflammation or irritation of a tendon, usually due to overuse or injury. Symptoms include pain and tenderness in the affected area, along with difficulty moving the hip.

3. Labral tear: This is a tear in the ring of cartilage that surrounds the hip joint. It can cause pain, stiffness, and a clicking or locking sensation in the hip.

4. Osteonecrosis: This is a condition in which there is a loss of blood supply to the bone, leading to bone death. It can cause pain, stiffness, and a limited range of motion in the hip joint.

5. Hip impingement: This occurs when there is abnormal contact between the bones in the hip joint, leading to pain, stiffness, and a limited range of motion.

Please note that this is not a definitive diagnosis, and only a qualified healthcare professional can provide an accurate diagnosis. If you are experiencing symptoms, it is important to consult with a healthcare professional as soon as possible. They can perform a physical examination, review your medical history, and conduct any necessary tests to determine the underlying cause of your symptoms and develop an appropriate treatment plan.

**Q5c**

Hip swelling and pain when walking, along with stiffness and cracking sounds when moving the hip, may be indicative of hip osteoarthritis. This condition is a degenerative joint disease that occurs when the protective cartilage on the ends of bones wears down over time, leading to pain, stiffness, and a limited range of motion in the joint.

However, there are other conditions that may also cause similar symptoms, including bursitis, tendinitis, labral tear, osteonecrosis, and hip impingement. It is important to consult with a healthcare professional to receive an accurate diagnosis and appropriate treatment plan.

**Day 2**

**Q1a**

Based on your symptoms of tingling, pain, and difficulty picking up small objects, it is possible that you are experiencing carpal tunnel syndrome. However, a definitive diagnosis can only be made by a medical professional after a thorough physical examination and diagnostic tests.

Here are five potential differential diagnoses that your doctor may consider:

1. Cubital tunnel syndrome: This is a condition that occurs when the ulnar nerve in the elbow is compressed, causing pain, tingling, and weakness in the hand and fingers.

2. Raynaud's disease: This is a condition that causes the blood vessels in the fingers to narrow, resulting in coldness, numbness, and pain in the fingers.

3. Peripheral neuropathy: This is a condition that occurs when there is damage to the peripheral nerves, causing pain, tingling, and weakness in the hands and feet.

4. Rheumatoid arthritis: This is an autoimmune disorder that can cause joint pain, stiffness, and swelling, as well as numbness and tingling in the hands.

5. Multiple sclerosis: This is a neurological disorder that can cause a wide range of symptoms, including numbness, tingling, and weakness in the hands and other parts of the body.

It's important to see a doctor for a proper diagnosis and treatment plan.

**Q1c**

Based on the symptoms you described, it's possible that you are experiencing carpal tunnel syndrome. This condition occurs when the median nerve, which runs through the wrist, becomes compressed or pinched, resulting in tingling, numbness, and pain in the hand and fingers. This may also lead to weakness and difficulty with fine motor tasks like picking up small objects.

Carpal tunnel syndrome is a common condition and can be caused by a variety of factors, including repetitive motions, injury, pregnancy, and certain medical conditions like diabetes and arthritis. Treatment options can include wrist splints, medication, physical therapy, or in some cases, surgery.

However, a definitive diagnosis can only be made by a medical professional after a thorough physical examination and diagnostic tests. It's important to see a doctor for a proper diagnosis and treatment plan.

**Q2a**

Based on the symptoms you described, it is possible that you are experiencing a neurological disorder such as multiple sclerosis (MS). MS is a condition that affects the central nervous system and can cause a wide range of symptoms, including numbness, tingling, and weakness in the hands, as well as difficulty with fine motor tasks and an unsteady gait.

However, a definitive diagnosis can only be made by a medical professional after a thorough physical examination and diagnostic tests.

Here are five potential differential diagnoses that your doctor may consider:

1. Peripheral neuropathy: This is a condition that occurs when there is damage to the peripheral nerves, causing numbness, tingling, and weakness in the hands and feet.

2. Parkinson's disease: This is a progressive neurological disorder that can cause tremors, stiffness, and difficulty with movement and coordination.

3. Carpal tunnel syndrome: This is a condition that occurs when the median nerve in the wrist is compressed, causing numbness, tingling, and weakness in the hand and fingers.

4. Amyotrophic lateral sclerosis (ALS): This is a progressive neurological disorder that affects the nerve cells responsible for controlling voluntary muscle movement, leading to muscle weakness, difficulty with fine motor tasks, and an unsteady gait.

5. Cervical spondylosis: This is a condition that occurs when the spinal discs in the neck degenerate, leading to pressure on the spinal cord and nerves, which can cause numbness, weakness, and difficulty with fine motor tasks.

It's important to see a doctor for a proper diagnosis and treatment plan.

Modified Q2b

Based on the symptoms you described, it's possible that you are experiencing carpal tunnel syndrome. This condition occurs when the median nerve, which runs through the wrist, becomes compressed or pinched, resulting in numbness, tingling, and pain in the hand and fingers. This may also lead to weakness and difficulty with fine motor tasks like buttoning a shirt.

Carpal tunnel syndrome is a common condition and can be caused by a variety of factors, including repetitive motions, injury, pregnancy, and certain medical conditions like diabetes and arthritis. Treatment options can include wrist splints, medication, physical therapy, or in some cases, surgery.

However, a definitive diagnosis can only be made by a medical professional after a thorough physical examination and diagnostic tests. It's important to see a doctor for a proper diagnosis and treatment plan.

**Q3b**

Based on the symptoms you described, it's possible that you are experiencing a condition called spinal stenosis. This is a narrowing of the spinal canal that puts pressure on the nerves in the lower back, which can cause pain, numbness, and weakness in the buttocks and legs. The pain often worsens with prolonged standing or walking but may improve when bending forward or sitting.

However, a definitive diagnosis can only be made by a medical professional after a thorough physical examination and diagnostic tests.

Here are five potential differential diagnoses that your doctor may consider:

1. Herniated disc: This is a condition that occurs when the soft tissue inside a spinal disc protrudes through the tougher outer layer, causing pressure on the surrounding nerves and resulting in pain and numbness in the lower back and legs.

2. Sciatica: This is a condition that occurs when the sciatic nerve, which runs from the lower back down through the legs, becomes compressed or pinched, resulting in pain, numbness, and tingling in the lower back and legs.

3. Osteoarthritis: This is a condition that occurs when the protective cartilage between bones wears down, causing pain, stiffness, and inflammation in the joints, including the lower back.

4. Piriformis syndrome: This is a condition that occurs when the piriformis muscle in the buttocks becomes tight or spasms, causing pressure on the sciatic nerve and resulting in pain, numbness, and tingling in the buttocks and legs.

5. Ankylosing spondylitis: This is a type of arthritis that primarily affects the spine, causing pain and stiffness in the lower back, as well as numbness and weakness in the legs.

It's important to see a doctor for a proper diagnosis and treatment plan.

**Q4a**

Based on the symptoms you described, it's possible that you are experiencing a knee injury or condition such as osteoarthritis or a meniscal tear.

Osteoarthritis is a common condition that occurs when the cartilage that cushions the joints breaks down, causing pain, stiffness, and swelling in the knee. Meniscal tears are injuries that occur when the cartilage in the knee is torn, often causing pain, swelling, and stiffness, as well as a cracking or popping sensation when bending the knee.

However, a definitive diagnosis can only be made by a medical professional after a thorough physical examination and diagnostic tests.

Here are five potential differential diagnoses that your doctor may consider:

1. Ligament injury: This is a type of knee injury that can occur when one of the ligaments that connect the bones in the knee is stretched or torn, often causing pain, swelling, and instability in the knee.

2. Bursitis: This is a condition that occurs when the bursae, small fluid-filled sacs that cushion the knee joint, become inflamed, causing pain, swelling, and stiffness in the knee.

3. Gout: This is a type of arthritis that occurs when uric acid crystals build up in the joints, causing sudden, severe pain, swelling, and redness in the knee.

4. Rheumatoid arthritis: This is an autoimmune condition that can affect the knee joint, causing pain, stiffness, and swelling, as well as deformity and loss of function over time.

5. Patellar tendinitis: This is a condition that occurs when the tendon that connects the kneecap to the shinbone becomes inflamed, causing pain and swelling in the knee.

It's important to see a doctor for a proper diagnosis and treatment plan.

**Q4c**

Based on the symptoms you described, it is possible that you have a knee condition called osteoarthritis. Osteoarthritis is a degenerative joint disease that occurs when the cartilage that cushions the ends of the bones in the knee joint wears away over time, causing pain, stiffness, and swelling.

Other potential causes of knee swelling and pain when walking, along with stiffness and cracking, include:

1. Meniscal tear: This is an injury to the cartilage in the knee joint that can cause pain, swelling, and stiffness, especially when bending the knee or twisting the leg.

2. Bursitis: This is a condition that occurs when the bursae, small fluid-filled sacs that cushion the knee joint, become inflamed, causing pain, swelling, and stiffness in the knee.

3. Ligament injury: This is a type of knee injury that can occur when one of the ligaments that connect the bones in the knee is stretched or torn, often causing pain, swelling, and instability in the knee.

4. Rheumatoid arthritis: This is an autoimmune condition that can affect the knee joint, causing pain, stiffness, and swelling, as well as deformity and loss of function over time.

5. Gout: This is a type of arthritis that occurs when uric acid crystals build up in the joints, causing sudden, severe pain, swelling, and redness in the knee.

It's important to see a doctor for a proper diagnosis and treatment plan.

**Q5a**

Based on the symptoms you described, it is possible that you have a hip condition such as osteoarthritis or a labral tear.

Osteoarthritis is a degenerative joint disease that occurs when the cartilage that cushions the ends of the bones in the hip joint wears away over time, causing pain, stiffness, and sometimes a cracking sound.

A labral tear is an injury to the cartilage that surrounds the hip joint, which can cause pain, stiffness, and a clicking or catching sensation when moving the hip.

Here are five potential differential diagnoses that your doctor may consider:

1. Hip bursitis: This is a condition that occurs when the bursae, small fluid-filled sacs that cushion the hip joint, become inflamed, causing pain and stiffness in the hip.

2. Hip impingement: This occurs when the bones in the hip joint rub against each other, causing pain, stiffness, and decreased range of motion in the hip.

3. Avascular necrosis: This is a condition that occurs when the blood supply to the bone in the hip joint is disrupted, causing the bone to die and leading to pain and stiffness in the hip.

4. Hip tendinitis: This is a condition that occurs when the tendons in the hip joint become inflamed, causing pain and stiffness in the hip.

5. Hip fracture: This is a break in the bone of the hip joint, usually caused by a fall or injury, which can cause pain, stiffness, and difficulty walking.

It's important to see a doctor for a proper diagnosis and treatment plan, which may include imaging tests such as X-rays or MRI to help determine the cause of your hip pain.

**Q5c**

Based on the symptoms you described, it is possible that you have a hip condition such as osteoarthritis, rheumatoid arthritis, or hip bursitis.

Osteoarthritis is a degenerative joint disease that occurs when the cartilage that cushions the ends of the bones in the hip joint wears away over time, causing pain, stiffness, and sometimes a cracking sound.

Rheumatoid arthritis is an autoimmune condition that can affect the hip joint, causing pain, stiffness, and swelling, as well as deformity and loss of function over time.

Hip bursitis is a condition that occurs when the bursae, small fluid-filled sacs that cushion the hip joint, become inflamed, causing pain and swelling in the hip.

Other potential causes of hip swelling and pain when walking, along with stiffness and cracking, include:

1. Hip labral tear: This is an injury to the cartilage that surrounds the hip joint, which can cause pain, stiffness, and a clicking or catching sensation when moving the hip.

2. Hip impingement: This occurs when the bones in the hip joint rub against each other, causing pain, stiffness, and decreased range of motion in the hip.

It's important to see a doctor for a proper diagnosis and treatment plan, which may include imaging tests such as X-rays or MRI to help determine the cause of your hip pain.

**Day 3**

**Q1a**

Based on your symptoms, the primary diagnosis that comes to mind is carpal tunnel syndrome (CTS), which is a common condition that affects the hand and wrist. It occurs when the median nerve, which runs from the forearm into the hand, becomes compressed at the wrist, causing pain, tingling, and numbness in the hand and fingers.

Here are five potential differential diagnoses that your doctor may consider:

1. Cubital tunnel syndrome: This is a condition that occurs when the ulnar nerve, which runs from the neck to the hand, becomes compressed at the elbow. It can cause similar symptoms to CTS, but may also affect the pinky and ring fingers.

2. Raynaud's disease: This is a condition that causes the blood vessels in the fingers and toes to narrow in response to cold temperatures or stress. It can cause pain, tingling, and numbness in the fingers.

3. Rheumatoid arthritis: This is a chronic inflammatory disorder that affects the joints, often causing pain, swelling, and stiffness. It can also affect the nerves and cause similar symptoms to CTS.

4. Thoracic outlet syndrome: This is a condition that occurs when the nerves and blood vessels that run from the neck to the arm become compressed as they pass through the thoracic outlet (the space between the collarbone and first rib). It can cause pain, tingling, and numbness in the arm and hand.

5. Diabetes: Diabetes can cause nerve damage (neuropathy) in the hands and feet, which can cause tingling, pain, and numbness.

It's important to consult with your healthcare provider for a proper diagnosis and treatment plan.

**Q1c**

Based on your symptoms, it is possible that you are experiencing carpal tunnel syndrome (CTS). CTS is a common condition that occurs when the median nerve, which runs from the forearm into the hand, becomes compressed at the wrist. This can cause pain, numbness, tingling, and weakness in the hand and fingers, particularly in the thumb, index, and middle fingers. Symptoms often worsen at night, and can make it difficult to grip or pick up small objects.

If you suspect you have CTS, it is important to see your healthcare provider for a proper diagnosis and treatment plan. They may recommend non-surgical options such as wrist splinting or physical therapy, or in more severe cases, surgery may be necessary.

**Q2a**

Based on your symptoms, the primary diagnosis that comes to mind is peripheral neuropathy. Peripheral neuropathy refers to damage or dysfunction of the nerves that carry information to and from the brain and spinal cord to the rest of the body, including the hands and feet. This can cause numbness, tingling, and weakness in the affected areas.

Here are five potential differential diagnoses that your healthcare provider may consider:

1. Multiple sclerosis (MS): MS is a chronic autoimmune disease that affects the central nervous system. It can cause a variety of symptoms, including numbness or tingling in the hands and feet, difficulty with coordination and balance, and weakness.

2. Diabetic neuropathy: This is a type of peripheral neuropathy that occurs as a complication of diabetes. It can cause numbness, tingling, and pain in the hands and feet, as well as difficulty with fine motor movements.

3. Carpal tunnel syndrome: CTS is a condition that occurs when the median nerve becomes compressed at the wrist, causing numbness, tingling, and weakness in the hand and fingers.

4. Parkinson's disease: Parkinson's disease is a neurodegenerative disorder that affects movement. It can cause tremors, rigidity, and difficulty with balance and coordination.

5. Cervical radiculopathy: This is a condition that occurs when a nerve root in the neck is compressed or inflamed, causing pain, numbness, and weakness in the arms and hands.

It's important to consult with your healthcare provider for a proper diagnosis and treatment plan. They may recommend diagnostic tests or imaging studies to determine the underlying cause of your symptoms, and develop an appropriate treatment plan.

**Q2b**

Based on your symptoms, one possible diagnosis is carpal tunnel syndrome (CTS). CTS is a common condition that occurs when the median nerve, which runs from the forearm into the hand, becomes compressed at the wrist. This can cause numbness, tingling, and weakness in the hand and fingers, particularly in the thumb, index, and middle fingers. Difficulty with fine movements and handling small objects is also a common symptom of CTS.

Other possible causes of numbness and difficulty with fine movements in the hands include:

1. Peripheral neuropathy: This refers to damage or dysfunction of the nerves that carry information to and from the brain and spinal cord to the rest of the body, including the hands and feet. This can cause numbness, tingling, and weakness in the affected areas.

2. Raynaud's disease: This is a condition that causes the blood vessels in the fingers and toes to narrow in response to cold temperatures or stress. It can cause numbness, tingling, and pain in the fingers.

3. Arthritis: Arthritis can cause joint pain, stiffness, and swelling, which can make fine movements and handling small objects difficult.

4. Multiple sclerosis (MS): MS is a chronic autoimmune disease that affects the central nervous system. It can cause a variety of symptoms, including numbness or tingling in the hands and feet, difficulty with coordination and balance, and weakness.

It's important to consult with your healthcare provider for a proper diagnosis and treatment plan. They may recommend diagnostic tests or imaging studies to determine the underlying cause of your symptoms, and develop an appropriate treatment plan.

**Q3b**

Based on your symptoms, the primary diagnosis that comes to mind is lumbar spinal stenosis. Lumbar spinal stenosis is a condition that occurs when the spinal canal in the lower back narrows and puts pressure on the spinal cord and nerves. This can cause pain, numbness, and weakness in the buttocks, legs, and feet, particularly during physical activity.

Here are five potential differential diagnoses that your healthcare provider may consider:

1. Herniated disc: A herniated disc occurs when the soft inner material of a disc in the spine pushes out through a crack in the outer layer, putting pressure on nearby nerves. This can cause pain and numbness in the buttocks, legs, and feet.

2. Sciatica: Sciatica refers to pain that radiates from the lower back down through the buttocks and legs, caused by compression or irritation of the sciatic nerve.

3. Osteoarthritis: Osteoarthritis is a degenerative joint disease that can affect the spine, causing pain, stiffness, and loss of mobility.

4. Spondylolisthesis: Spondylolisthesis is a condition in which one vertebra in the spine slips forward over the one below it, putting pressure on nearby nerves and causing pain, numbness, and weakness in the legs.

5. Piriformis syndrome: Piriformis syndrome occurs when the piriformis muscle, located in the buttocks, spasms or tightens, putting pressure on the sciatic nerve and causing pain and numbness in the buttocks and legs.

It's important to consult with your healthcare provider for a proper diagnosis and treatment plan. They may recommend diagnostic tests or imaging studies to determine the underlying cause of your symptoms, and develop an appropriate treatment plan.

**Q4a**

Based on your symptoms, the primary diagnosis that comes to mind is osteoarthritis of the knee. Osteoarthritis is a degenerative joint disease that can affect any joint in the body, including the knee. It occurs when the protective cartilage that cushions the joints wears down over time, leading to pain, stiffness, and swelling.

Here are five potential differential diagnoses that your healthcare provider may consider:

1. Rheumatoid arthritis: Rheumatoid arthritis is an autoimmune disorder that can cause inflammation and damage to the joints, including the knee. It can cause pain, stiffness, and swelling, and may also lead to deformity of the joint over time.

2. Gout: Gout is a type of arthritis caused by a buildup of uric acid crystals in the joints, which can cause pain, swelling, and stiffness. It often affects the big toe, but can also affect the knee.

3. Meniscus tear: The meniscus is a piece of cartilage in the knee that helps cushion and stabilize the joint. A tear in the meniscus can cause pain, swelling, and stiffness in the knee, particularly when walking or bending the knee.

4. Bursitis: Bursitis is inflammation of the bursae, small fluid-filled sacs that cushion and lubricate joints. It can cause pain, swelling, and stiffness in the knee, particularly when walking or kneeling.

5. Ligament injury: An injury to the ligaments in the knee, such as the anterior cruciate ligament (ACL), can cause pain, swelling, and instability in the knee, particularly during physical activity.

It's important to consult with your healthcare provider for a proper diagnosis and treatment plan. They may recommend diagnostic tests or imaging studies to determine the underlying cause of your symptoms, and develop an appropriate treatment plan.

**Q4c**

Based on your symptoms, the most likely diagnosis is osteoarthritis of the knee. Osteoarthritis is a degenerative joint disease that can cause the cartilage in the knee joint to wear down over time, leading to pain, stiffness, and swelling. The cracking sound you hear when bending your knee is called crepitus, which is common in people with osteoarthritis.

However, other conditions can also cause knee swelling and pain. Here are five potential differential diagnoses that your healthcare provider may consider:

1. Rheumatoid arthritis: Rheumatoid arthritis is an autoimmune disorder that can cause inflammation and damage to the joints, including the knee. It can cause pain, stiffness, and swelling, and may also lead to deformity of the joint over time.

2. Meniscal tear: A meniscal tear is a tear in the cartilage that cushions the knee joint. It can cause pain, swelling, and stiffness, particularly when walking or bending the knee.

3. Bursitis: Bursitis is inflammation of the bursae, small fluid-filled sacs that cushion and lubricate joints. It can cause pain, swelling, and stiffness in the knee, particularly when walking or kneeling.

4. Ligament injury: An injury to the ligaments in the knee, such as the anterior cruciate ligament (ACL), can cause pain, swelling, and instability in the knee, particularly during physical activity.

5. Tendinitis: Tendinitis is inflammation of a tendon, which is the tissue that connects muscle to bone. It can cause pain, swelling, and stiffness in the knee, particularly around the knee cap.

It's important to consult with your healthcare provider for a proper diagnosis and treatment plan. They may recommend diagnostic tests or imaging studies to determine the underlying cause of your symptoms, and develop an appropriate treatment plan.

**Q5a**

Based on your symptoms, the primary diagnosis that comes to mind is osteoarthritis of the hip. Osteoarthritis is a degenerative joint disease that can cause the cartilage in the hip joint to wear down over time, leading to pain, stiffness, and cracking sounds. It's a common condition in older adults, but it can occur at any age.

Here are five potential differential diagnoses that your healthcare provider may consider:

1. Bursitis: Bursitis is inflammation of the bursae, small fluid-filled sacs that cushion and lubricate joints. It can cause pain and stiffness in the hip, particularly when walking or lying on the affected side.

2. Tendinitis: Tendinitis is inflammation of a tendon, which is the tissue that connects muscle to bone. It can cause pain and stiffness in the hip, particularly in the outer part of the hip.

3. Labral tear: The labrum is a piece of cartilage that lines the hip joint. A tear in the labrum can cause pain and stiffness in the hip, particularly during physical activity.

4. Hip impingement: Hip impingement occurs when the bones in the hip joint rub against each other, causing pain and stiffness. It's more common in younger adults and athletes.

5. Avascular necrosis: Avascular necrosis is a condition where the blood supply to the bone in the hip joint is disrupted, causing the bone to die. It can cause pain and stiffness in the hip, particularly during physical activity.

It's important to consult with your healthcare provider for a proper diagnosis and treatment plan. They may recommend diagnostic tests or imaging studies to determine the underlying cause of your symptoms, and develop an appropriate treatment plan.

**Q5c**

Based on your symptoms, the most likely diagnosis is osteoarthritis of the hip. Osteoarthritis is a degenerative joint disease that can cause the cartilage in the hip joint to wear down over time, leading to pain, stiffness, and swelling. The cracking sound you hear when moving your hip is called crepitus, which is common in people with osteoarthritis.

However, other conditions can also cause hip swelling and pain. Here are five potential differential diagnoses that your healthcare provider may consider:

1. Rheumatoid arthritis: Rheumatoid arthritis is an autoimmune disorder that can cause inflammation and damage to the joints, including the hip. It can cause pain, stiffness, and swelling, and may also lead to deformity of the joint over time.

2. Bursitis: Bursitis is inflammation of the bursae, small fluid-filled sacs that cushion and lubricate joints. It can cause pain, swelling, and stiffness in the hip, particularly when walking or lying on the affected side.

3. Avascular necrosis: Avascular necrosis is a condition where the blood supply to the bone in the hip joint is disrupted, causing the bone to die. It can cause pain and stiffness in the hip, particularly during physical activity.

4. Labral tear: The labrum is a piece of cartilage that lines the hip joint. A tear in the labrum can cause pain, stiffness, and clicking or locking of the hip joint.

5. Hip impingement: Hip impingement occurs when the bones in the hip joint rub against each other, causing pain and stiffness. It's more common in younger adults and athletes.

It's important to consult with your healthcare provider for a proper diagnosis and treatment plan. They may recommend diagnostic tests or imaging studies to determine the underlying cause of your symptoms, and develop an appropriate treatment plan.

**Day 4**

**Q1a**

Based on your symptoms, one possible primary diagnosis could be carpal tunnel syndrome, which is a condition caused by pressure on the median nerve as it passes through the carpal tunnel in the wrist.

Five potential differential diagnoses could be:

1. Cubital tunnel syndrome: This is another type of nerve compression syndrome, where the ulnar nerve becomes compressed or irritated at the elbow, leading to similar symptoms to carpal tunnel syndrome, including tingling and pain in the fingers.

2. Peripheral neuropathy: This is a disorder that affects the nerves outside of the brain and spinal cord, which can cause tingling, numbness, or weakness in the hands and feet.

3. Rheumatoid arthritis: This is a chronic autoimmune disorder that causes inflammation in the joints, leading to pain, stiffness, and swelling in the hands and wrists.

4. Dupuytren's contracture: This is a condition where the tissues in the palm of the hand become thick and tight, leading to the fingers being pulled towards the palm, making it difficult to pick up objects.

5. Trigger finger: This is a condition where the tendons in the finger become inflamed or swollen, causing the finger to get stuck in a bent position, and leading to pain and difficulty with fine motor tasks like grasping small objects.

**Q1c**

Based on your symptoms, it is possible that you are experiencing carpal tunnel syndrome. This is a condition caused by compression of the median nerve as it passes through the carpal tunnel in the wrist. Symptoms may include tingling, numbness, or pain in the fingers and hand, particularly at night or during activities that involve the wrist. Difficulty with fine motor tasks like picking up small objects can also be a symptom of carpal tunnel syndrome.

It is important to note that a proper diagnosis requires a medical evaluation by a healthcare professional. Therefore, it is recommended that you see your doctor for an evaluation and appropriate treatment.

**Q2a**

Based on your symptoms, a possible primary diagnosis could be peripheral neuropathy. Peripheral neuropathy refers to damage or dysfunction of the peripheral nerves that transmit signals between the central nervous system (brain and spinal cord) and the rest of the body. This can result in numbness, tingling, or weakness in the hands and feet, difficulty with fine motor movements, and an unsteady gait.

Five potential differential diagnoses could be:

1. Multiple sclerosis: This is a chronic autoimmune disorder that affects the central nervous system and can cause a variety of symptoms, including numbness or tingling in the hands or feet, difficulty with fine motor movements, and an unsteady gait.

2. Parkinson's disease: This is a progressive neurological disorder that affects movement and can cause tremors, stiffness, and difficulty with fine motor movements, including handling small objects.

3. Guillain-Barré syndrome: This is a rare autoimmune disorder that affects the peripheral nervous system, causing weakness, numbness, or tingling in the limbs, difficulty with fine motor movements, and an unsteady gait.

4. Carpal tunnel syndrome: This is a condition caused by compression of the median nerve as it passes through the carpal tunnel in the wrist, which can cause numbness or tingling in the hands and difficulty with fine motor movements.

5. Vitamin B12 deficiency: This is a condition that can result in nerve damage, leading to numbness or tingling in the hands and feet, difficulty with fine motor movements, and an unsteady gait.

**Q2b**

Based on your symptoms, one possible disease that could be causing your symptoms is carpal tunnel syndrome. This is a condition caused by compression of the median nerve as it passes through the carpal tunnel in the wrist. Symptoms can include numbness, tingling, or pain in the hand and fingers, particularly at night or during activities that involve the wrist. Difficulty with fine motor movements, such as handling small objects or buttoning a shirt, can also be a symptom of carpal tunnel syndrome.

However, it is important to note that there can be other potential causes of these symptoms, and a proper diagnosis would require a medical evaluation by a healthcare professional. Therefore, it is recommended that you see your doctor for an evaluation and appropriate treatment.

**Q3b**

Based on your symptoms, a possible primary diagnosis could be spinal stenosis. Spinal stenosis is a condition where the spinal canal narrows, which can put pressure on the spinal cord and nerves. Symptoms can include pain in the lower back, numbness and pain in the buttocks and calves, and increased pain with standing or walking that improves with leaning forward.

Five potential differential diagnoses could be:

1. Herniated disc: This is a condition where the soft tissue between the vertebrae in the spine bulges or ruptures, putting pressure on the nerves and causing pain, numbness, or weakness in the buttocks, legs, or feet.

2. Sciatica: This is a condition caused by irritation or compression of the sciatic nerve, which runs from the lower back down to the legs. Symptoms can include pain, numbness, or tingling in the lower back, buttocks, and legs.

3. Spondylolisthesis: This is a condition where a vertebra in the spine slips out of place, which can put pressure on the nerves and cause pain, numbness, or weakness in the lower back, buttocks, and legs.

4. Piriformis syndrome: This is a condition where the piriformis muscle in the buttocks becomes tight or inflamed, putting pressure on the sciatic nerve and causing pain, numbness, or tingling in the buttocks and legs.

5. Osteoarthritis: This is a condition where the cartilage in the joints breaks down, leading to pain, stiffness, and inflammation. In the lower back, this can lead to pain, stiffness, and decreased mobility, which can contribute to or exacerbate spinal stenosis.

**Q4a**

Based on your symptoms, a possible primary diagnosis could be knee osteoarthritis. Knee osteoarthritis is a degenerative joint disease where the cartilage in the knee joint breaks down, causing pain, stiffness, and swelling.

Five potential differential diagnoses could be:

1. Knee ligament injury: This is a type of injury to the ligaments that support the knee joint, which can cause pain, swelling, and instability in the knee.

2. Meniscus tear: This is a tear in the cartilage that cushions the knee joint, which can cause pain, swelling, and stiffness, as well as a popping or clicking sensation when bending the knee.

3. Bursitis: This is inflammation of the bursae, which are small fluid-filled sacs that cushion the joints. In the knee, bursitis can cause pain, swelling, and stiffness.

4. Rheumatoid arthritis: This is an autoimmune disorder that can affect the joints, including the knee. Symptoms can include pain, swelling, and stiffness in the knee joint, as well as fatigue and fever.

5. Gout: This is a type of arthritis caused by a buildup of uric acid crystals in the joints. In the knee, gout can cause sudden and severe pain, swelling, and redness.

**Q4c**

Based on your symptoms, a possible disease that could be causing your knee problems is knee osteoarthritis. Knee osteoarthritis is a degenerative joint disease where the cartilage in the knee joint breaks down, causing pain, stiffness, and swelling. The cracking sound you hear when bending your knee is known as crepitus, which can also be a symptom of osteoarthritis.

However, other potential causes of knee swelling and pain include ligament injuries, meniscus tears, bursitis, rheumatoid arthritis, and gout. It is important to see a healthcare professional for an evaluation to determine the underlying cause of your symptoms and to develop an appropriate treatment plan.

**Q5a**

Based on your symptoms, a possible primary diagnosis could be hip osteoarthritis. Hip osteoarthritis is a degenerative joint disease where the cartilage in the hip joint breaks down, causing pain, stiffness, and swelling. The cracking sound you hear when moving your hip is known as crepitus, which can also be a symptom of osteoarthritis.

Five potential differential diagnoses could be:

1. Hip labral tear: This is a tear in the ring of cartilage that surrounds the hip joint, which can cause pain, stiffness, and clicking or locking sensations in the hip.

2. Hip bursitis: This is inflammation of the bursae, which are small fluid-filled sacs that cushion the joints. In the hip, bursitis can cause pain and stiffness, especially when walking or standing.

3. Hip fracture: This is a break in the bones of the hip, which can cause sudden and severe pain, swelling, and difficulty bearing weight on the affected leg.

4. Avascular necrosis: This is a condition where the bone tissue in the hip joint dies due to a lack of blood supply. Symptoms can include pain, stiffness, and difficulty walking.

5. Piriformis syndrome: This is a condition where the piriformis muscle in the buttocks becomes tight or inflamed, putting pressure on the sciatic nerve and causing pain, numbness, or tingling in the hip and/or leg.

**Q5c**

Based on your symptoms, a possible disease that could be causing your hip problems is hip osteoarthritis. Hip osteoarthritis is a degenerative joint disease where the cartilage in the hip joint breaks down, causing pain, stiffness, and swelling. The cracking sound you hear when moving your hip is known as crepitus, which can also be a symptom of osteoarthritis.

However, other potential causes of hip swelling and pain include hip labral tear, hip bursitis, hip fracture, avascular necrosis, and piriformis syndrome. It is important to see a healthcare professional for an evaluation to determine the underlying cause of your symptoms and to develop an appropriate treatment plan.

**Day 5**

**Q1a**

Based on your symptoms, it is possible that you may have carpal tunnel syndrome. This condition occurs when the median nerve, which runs from your forearm to your hand, is compressed or squeezed as it travels through the wrist at the carpal tunnel. The symptoms of carpal tunnel syndrome often include tingling, numbness, pain, and weakness in the affected hand and fingers, especially at night.

However, it is important to note that there can be other causes for these symptoms, so a proper diagnosis would require a medical evaluation by a healthcare professional. Some potential differential diagnoses for your symptoms include:

1. Cubital tunnel syndrome: This is a condition where the ulnar nerve, which runs from your elbow to your hand, is compressed or irritated, leading to similar symptoms as carpal tunnel syndrome. However, the symptoms usually affect the pinky and ring fingers instead of the thumb and index finger.

2. Peripheral neuropathy: This is a condition that results from damage to the peripheral nerves, which can cause tingling, pain, and numbness in the hands and feet.

3. Thoracic outlet syndrome: This is a condition that occurs when nerves and blood vessels in the thoracic outlet (the space between your collarbone and first rib) are compressed, leading to symptoms such as pain and numbness in the arm, hand, and fingers.

4. Rheumatoid arthritis: This is a chronic autoimmune disorder that can cause joint pain and inflammation, and may affect the hands and fingers.

5. Multiple sclerosis: This is a chronic neurological condition that can cause a range of symptoms, including tingling, numbness, and weakness in the hands and fingers. However, this condition would typically present with a wider range of symptoms beyond the hands and fingers.

Please note that this list is not exhaustive, and a proper diagnosis would require a medical evaluation.

Modified Q1c

Based on your symptoms, it is possible that you may have carpal tunnel syndrome. This condition occurs when the median nerve, which runs from your forearm to your hand, is compressed or squeezed as it travels through the wrist at the carpal tunnel. The symptoms of carpal tunnel syndrome often include tingling, numbness, pain, and weakness in the affected hand and fingers, especially at night. Difficulty picking up small objects can also be a symptom of carpal tunnel syndrome. However, a proper diagnosis would require a medical evaluation by a healthcare professional.

**Q2a**

Based on your symptoms, it is possible that you may have peripheral neuropathy. This is a condition that results from damage to the peripheral nerves, which can cause numbness, tingling, and weakness in the hands and feet. Difficulty with fine movements and an unsteady walk can also be symptoms of peripheral neuropathy. However, a proper diagnosis would require a medical evaluation by a healthcare professional.

Some potential differential diagnoses for your symptoms include:

1. Multiple sclerosis: This is a chronic neurological condition that can cause a wide range of symptoms, including numbness, tingling, and weakness in the hands and feet. An unsteady walk can also be a symptom of this condition.

2. Charcot-Marie-Tooth disease: This is an inherited disorder that affects the peripheral nerves and can cause muscle weakness, numbness, and difficulty with fine motor skills.

3. Diabetic neuropathy: This is a type of peripheral neuropathy that can occur in people with diabetes. It can cause numbness, tingling, and weakness in the hands and feet, and can also affect fine motor skills.

4. Vitamin B12 deficiency: A deficiency in vitamin B12 can lead to nerve damage, which can cause numbness, tingling, and weakness in the hands and feet.

5. Guillain-Barré syndrome: This is a rare disorder in which the body's immune system attacks the peripheral nerves, leading to muscle weakness, numbness, and tingling. An unsteady walk can also be a symptom of this condition.

Please note that this list is not exhaustive, and a proper diagnosis would require a medical evaluation.

**Q2b**

Based on your symptoms, it is possible that you may have carpal tunnel syndrome. This condition occurs when the median nerve, which runs from your forearm to your hand, is compressed or squeezed as it travels through the wrist at the carpal tunnel. The symptoms of carpal tunnel syndrome often include numbness, tingling, weakness, and difficulty with fine motor skills in the affected hand, especially in the thumb, index finger, middle finger, and half of the ring finger. Difficulty with fine movements to handle small objects, such as buttoning a shirt, is also a common symptom of carpal tunnel syndrome. However, a proper diagnosis would require a medical evaluation by a healthcare professional.

**Q3b**

Based on your symptoms, it is possible that you may have lumbar spinal stenosis. This condition occurs when the spinal canal in the lower back narrows and puts pressure on the spinal cord and nerves, which can cause pain, numbness, and weakness in the buttocks, legs, and feet. The pain may increase when walking or standing for a prolonged period of time, but can improve when you lean forward to relieve the pressure on the nerves. However, a proper diagnosis would require a medical evaluation by a healthcare professional.

Some potential differential diagnoses for your symptoms include:

1. Herniated disc: This is a condition where the soft tissue inside a spinal disc pushes out and irritates nearby nerves, causing pain, numbness, and weakness in the affected area.

2. Sciatica: This is a condition that occurs when the sciatic nerve, which runs from the lower back down through the legs, becomes compressed or irritated. It can cause pain, numbness, and tingling in the buttocks and legs.

3. Peripheral artery disease: This is a condition that occurs when the blood vessels in the legs become narrowed or blocked, leading to decreased blood flow to the legs and feet. It can cause pain, numbness, and weakness in the legs, especially during physical activity.

4. Piriformis syndrome: This is a condition that occurs when the piriformis muscle in the buttocks becomes tight or spasms and irritates the nearby sciatic nerve, causing pain and numbness in the buttocks and legs.

5. Osteoarthritis: This is a degenerative joint disease that can affect the spine and cause pain and stiffness in the lower back. In some cases, it can also cause nerve compression and lead to symptoms such as numbness and weakness in the legs.

Please note that this list is not exhaustive, and a proper diagnosis would require a medical evaluation.

**Q4a**

Based on your symptoms, it is possible that you may have knee osteoarthritis. This is a degenerative joint disease that can occur when the cartilage in the knee joint wears down over time, leading to pain, swelling, stiffness, and cracking sounds when moving the knee. However, a proper diagnosis would require a medical evaluation by a healthcare professional.

Some potential differential diagnoses for your symptoms include:

1. Rheumatoid arthritis: This is a chronic autoimmune disease that can affect the joints, including the knee, and cause pain, swelling, stiffness, and deformities. It often affects multiple joints symmetrically and can cause systemic symptoms such as fatigue and fever.

2. Meniscus tear: This is a common knee injury that occurs when the cartilage between the thigh bone and the shin bone is torn. It can cause pain, swelling, and stiffness in the knee joint, especially when moving or twisting the knee.

3. Bursitis: This is a condition that occurs when the bursae, small fluid-filled sacs that cushion the joints, become inflamed. It can cause pain, swelling, and tenderness in the affected area, including the knee.

4. Gout: This is a type of arthritis that occurs when uric acid crystals build up in the joints, causing pain, swelling, and redness. It often affects the big toe joint but can also affect the knee joint.

5. Patellofemoral pain syndrome: This is a condition that occurs when the patella (kneecap) does not track properly in the femoral groove, causing pain and stiffness in the knee joint, especially when walking or climbing stairs.

Please note that this list is not exhaustive, and a proper diagnosis would require a medical evaluation.

**Q4c**

Based on your symptoms, it is possible that you may have knee osteoarthritis. This is a degenerative joint disease that can occur when the cartilage in the knee joint wears down over time, leading to pain, swelling, stiffness, and cracking sounds when moving the knee. However, a proper diagnosis would require a medical evaluation by a healthcare professional.

It is important to note that knee swelling and pain can also be caused by other conditions, such as ligament or meniscus injuries, bursitis, tendonitis, or rheumatoid arthritis. Therefore, if you are experiencing knee swelling and pain, it is recommended that you see a healthcare professional for a proper evaluation and diagnosis.

**Q5a**

Based on your symptoms, it is possible that you may have hip osteoarthritis. This is a degenerative joint disease that can occur when the cartilage in the hip joint wears down over time, leading to pain, stiffness, and cracking sounds when moving the hip. However, a proper diagnosis would require a medical evaluation by a healthcare professional.

Some potential differential diagnoses for your symptoms include:

1. Hip bursitis: This is a condition that occurs when the bursae, small fluid-filled sacs that cushion the joints, become inflamed. It can cause pain and stiffness in the hip joint, especially when walking or climbing stairs.

2. Tendinitis: This is a condition that occurs when the tendons, which connect muscles to bones, become inflamed. It can cause pain and stiffness in the hip joint, especially when moving or lifting the leg.

3. Hip labral tear: This is a tear in the cartilage that surrounds the hip joint. It can cause pain and stiffness in the hip joint, especially when moving or twisting the hip.

4. Avascular necrosis: This is a condition that occurs when the blood supply to the hip joint is disrupted, leading to bone tissue death. It can cause pain and stiffness in the hip joint, especially when walking or bearing weight on the affected hip.

5. Hip fracture: This is a break in the bone of the hip joint, usually caused by a fall or injury. It can cause severe pain and difficulty moving the affected leg.

Please note that this list is not exhaustive, and a proper diagnosis would require a medical evaluation.

**Q5c**

Based on your symptoms, it is possible that you may have hip osteoarthritis. This is a degenerative joint disease that can occur when the cartilage in the hip joint wears down over time, leading to pain, swelling, stiffness, and cracking sounds when moving the hip. However, a proper diagnosis would require a medical evaluation by a healthcare professional.

It is important to note that hip swelling and pain can also be caused by other conditions, such as hip bursitis, tendinitis, hip labral tear, avascular necrosis, or hip fracture. Therefore, if you are experiencing hip swelling and pain, it is recommended that you see a healthcare professional for a proper evaluation and diagnosis.
